# Global Burden, Trends, and Inequalities of Gallbladder and Biliary Tract Cancer, 1990–2021: A Decomposition and Age–Period–Cohort Analysis

**DOI:** 10.1111/liv.16199

**Published:** 2025-01-01

**Authors:** Sen Lei, Guizhong Huang, Xiaohui Li, Pu Xi, Zehui Yao, Xiaojun Lin

**Affiliations:** ^1^ Department of Pancreatobiliary Surgery, State Key Laboratory of Oncology in South China, Guangdong Key Laboratory of Nasopharyngeal Carcinoma Diagnosis and Therapy, Guangdong Provincial Clinical Research Center for Cancer Sun Yat‐Sen University Cancer Center Guangzhou P. R. China; ^2^ Department of Hepatobiliary Surgery The Third Affiliated Hospital of Sun Yat‐Sen University, Sun Yat‐Sen University Guangzhou China

**Keywords:** age–period–cohort analysis, age‐standardised rate, decomposition analysis, gallbladder and biliary tract cancer, global burden of disease

## Abstract

**Background:**

Gallbladder and biliary tract cancer (GBTC) increasingly aggravates the global malignancy burden. This study aimed to evaluate the updated condition of GBTC temporal burden trends and inequalities from 1990 to 2021.

**Methods:**

Data on GBTC were extracted from the Global Burden of Disease (GBD) 2021 study. Incidence, deaths, and disability‐adjusted life years (DALYs) and their age‐standardised rates (ASR) were quantified from 1990 to 2021, stratified by sex, age and sociodemographic index (SDI). The age–period–cohort (APC) model was used to elucidate the effects of age, period, and cohort. Decomposition analysis and cross‐country inequality evaluation were performed to assess the contributing factors and disease imbalance, respectively. Bayesian APC analysis was used to estimate the future burden.

**Results:**

In 2021, the global incident cases of GBTC were 216 768, with 171 961 deaths and 3 732 121 DALYs lost. From 1990 to 2021, the ASR of incidence, mortality, and DALYs decreased slightly. Males showed a slight increase in ASR of incidence, while females experienced a significant decrease. High‐income regions, particularly in Asia Pacific and Latin America, exhibited a higher burden, while Western Sub‐Saharan Africa had the lowest. Low and low‐middle SDI regions showed a gradual rise in all metrics despite lower absolute numbers. The APC analysis indicated that the global incidence of GBTC tended to rise with age, but gender differences existed. Besides, a deteriorating cohort effect was detected amongst individuals born between 1907 and 1917. Decomposition analysis revealed that population growth was the primary driver of the increased GBTC burden globally. Significant disparities in GBTC burden by SDI were observed, with a notable decline in inequality over time. Projections indicated a slow decline in the global ASR through 2040, with a more pronounced decrease in females.

**Conclusions:**

There are significant regional and gender differences in the global burden of GBTC. Population growth remains a major contributor to the burden. Despite the overall decline, the increasing incidence in low and lower‐middle SDI regions and the persistent male burden highlight the need for targeted interventions. Future efforts should focus on addressing socio‐economic inequalities and reducing risk factors, particularly in vulnerable populations.

**Summary**

- The burden of gallbladder and biliary tract cancer showed a slight decline in age‐standardised rates of incidence, mortality, and disability‐adjusted life years from 1990 to 2021, despite overall global increases in case numbers driven largely by population growth.
- Gender‐specific trends in age‐standardised incidence rates were observed, with males showing a slight increase, whereas females experienced a notable decline.
- Projections indicate a gradual decrease in age‐standardised rates of incidence, mortality, and disability‐adjusted life years through 2040, with more pronounced declines anticipated for females.

**Abbreviations**
AAPC: average annual percent change
APC: age–period–cohort
ASDR: age‐standardised disability‐adjusted life years rate
ASIR: age‐standardised incidence rate
ASMR: age‐standardised mortality rate
ASR: age‐standardised rate
BAPC: Bayesian age–period–cohort analysis
BMI: body mass index
CI: confidence interval
CODEm: cause of death ensemble modelling
DALYs: disability‐adjusted life years
GBD: global burden of disease
GBTC: gallbladder and biliary duct cancer
ICD: International Classification of Diseases and Injuries
IHME: Institute for Health Metrics and Evaluation
INLA: integrated nested Laplace approximation
MASLD: metabolic dysfunction‐associated steatotic liver disease
SDI: socio‐demographic index
SII: slope index of inequality
UI: uncertainty interval
YLD: years lived with disability
YLL: years of life lost

## 1 Introduction

Gallbladder and biliary duct cancer (GBTC) is a rare but extremely lethal cancer originating from the gallbladder and intrahepatic and extrahepatic bile ducts, accounting for about 4% of all gastrointestinal cancers, with a 5‐year overall survival rate of 5%–15% [1, 2, 3]. It is an undeniable fact that, even with a relatively low incidence rate, overall morbidity, deaths, and burden of GBTC are aggravating [3, 4]. Recently, GBTC ranks 21st in terms of cancer incidence rates and is the 17th leading cause of cancer‐related deaths in men and the 14th in women [3]. However, according to current projections, there will be over 50 000 new cases of hepatobiliary and gallbladder tumours diagnosed in the United States in 2024, with approximately 35 000 annual deaths [5]. Thus, it is profitable for government agencies and academic research institutes to do timely work on disease prevention and control when the worldwide burden of GBTC is timely evaluated.

Several factors, including but not limited to race, ethnicity, eating habits, lifestyle, and religious beliefs, affect the incidence, mortality, and burden of GBTC differently across nations and locations [1, 6, 7, 8]. For instance, it has been proven that Native American populations in the Americas, including Mapuche Indians, Alaska Natives, and Hispanics, have some of the highest incidence rates of gallbladder cancer globally [9]. Cholelithiasis is proven as the risk factor for GBTC. According to epidemiological studies, regional differences in its prevalence may contribute to the global variances in the prevalence patterns of biliary tract tumours [8]. Specifically, the highest incidence rates of GBTC have been observed in South America, South and Central Asia, and South Africa [10]. Kim et al. reported that non‐Hispanic Asians in the US had higher death rates for intrahepatic and extrahepatic cholangiocarcinoma compared to Hispanic and non‐Hispanic Black individuals [1].

Although GBTC is relatively rare globally, their high mortality rates and poor prognosis underscore the need for effective therapeutic strategies. Historically, treatment options for GBTC were limited, with gemcitabine‐based chemotherapy being the standard approach [11]. However, recent advancements in systemic therapies—such as targeted therapies for molecular alterations (e.g., IDH1 mutations, FGFR2 fusions) and immune checkpoint inhibitors—are beginning to reshape the treatment landscape, offering hope for improved outcomes, particularly in advanced stages [11]. Despite these advancements, GBTC remains challenging to treat, with high recurrence rates and limited efficacy of current therapies. Consequently, a deeper understanding of GBTC's unique biology and further research into combination strategies, biomarkers, and resistance mechanisms is crucial to improving outcomes [12, 13]. Addressing healthcare inequalities, alongside continued innovation in GBTC treatment, is crucial for reducing the global burden and improving survival outcomes across diverse populations.

Given the need for making appropriate preventative and therapeutic plans, a comprehensive and updated evaluation of the health burden of GBTC is necessary. Such an evaluation aids in the efficient reallocation of medical resources and the formulation of policies. The Global Burden of Disease (GBD) study systematically pictures the incidence, prevalence, mortality rates, and health effects resulting from various illnesses and injuries globally, thus promoting a comprehensive picture of global health challenges [14]. In this study, we conducted a comprehensive evaluation of the up‐to‐date data from the GBD study to update the incidence, mortality, and disability‐adjusted life years (DALYs) for GBTC at the national, regional, and global levels. The Socio‐Demographic Index (SDI), age‐specific variations, gender inequality, and regional disparities were all taken into consideration, which helped to provide insightful information and strategic direction for making public health policies.

## 2 Methods and Materials

### 2.1 Data Source

The Institute for Health Metrics and Evaluation (IHME) carried out the GBD 2021 study to evaluate the burden of 371 diseases and injuries across 204 nations and territories [14]. Compared with the GBD 2019, the 2021 study made important methodological changes, added 12 additional causes, and included 19 189 new data sources for DALYs [14]. They took standardised approaches to obtain estimates for many health parameters, such as prevalence, incidence, mortality, years of life lost (YLL), years lived with disability (YLD), and DALYs, which contributed to ensuring consistency and comparability across regions and different periods.

In this study, we used data on GBTC from 1990 to 2021 by the GBD Results Tool (https://vizhub.healthdata.org/gbd‐results/). The data were stratified by sex, age, and SDI. The detailed information of the GBD study on incidence, deaths, and DALYs contributed to a precise evaluation of the GBTC burden and trend over time. All analytical processes followed the Guidelines for Accurate and Transparent Health Estimates Reporting [15].

### 2.2 Estimation Framework

The GBD study also used scientific modelling methods to measure the burden of GBTC. Firstly, the DisMod‐MR 2.1 tool was selected to estimate the GBTC incidence [14, 16]. This software comprehensively evaluated the effect of disease parameters, epidemiological associations, and geospatial data to get reasonable estimates. We used the Cause of Death Ensemble modelling (CODEm) framework to perform mortality assessment [14, 16]. The framework synthesises multiple registrations and verbal autopsy data. Moreover, the extracted data were rigorously adjusted for accuracy before analysis. By applying these models to the 2021 GBD database, the study generated an extensive evaluation of the global GBTC burden, considering the variations in study design and methodology across different data sources. The assessment of GBTC‐related DALYs involved aggregating YLD, which quantified the burden of living with the condition, and the YLL, which accounted for the impact of premature mortality.

### 2.3 Case Definition

GBTC is characterised by the following codes: C23, C24–C24.9 in the 10th revision of the International Classification of Diseases and Injuries (ICD‐10), and 156–156.9 in ICD‐9.

### 2.4 Socio‐Demographic Index

SDI was calculated to evaluate the socio‐economic level across regions. This composite index takes country‐level per capita income, average educational attainment, and total fertility rate into consideration, with a score ranging from 0 to 1 [14]. An elevated SDI value indicates richer socio‐economic development. Based on the calculated SDI values, the global regions were divided into five groups: low, low‐middle, middle, high‐middle, and high SDI regions.

### 2.5 Age–Period–Cohort Analysis

The age–period–cohort (APC) model is a widely accepted statistical analysis in demographic, sociological, and epidemiological research [17]. Specifically, the model takes full consideration of the potential impact of age (diagnosis age), period (diagnosis year), and cohort (birth date) on the risk of the objective. In this study, we first drew longitudinal age cures to present the longitudinal incidence rate after the adjustment period and cohort effects. Then, the period/cohort rate ratio was calculated to draw line charts. The ratio signified the GBTC occurrence risk after excluding the impact from the non‐linear confounder. The whole group involved 17 5‐year age cohorts (15–19 to 95+) and 5 5‐year periods (1992–2021), and the median period (2007–2011) and cohort (1952) were used as the reference set. Thirdly, net and local drifts were summarised as annual percentage changes in ASR and age‐specific rates over time, respectively.

### 2.6 Decomposition Analysis

Decomposition analysis is a valuable tool for disentangling complex factors that contribute to observed changes in disease incidence, mortality, and other metrics over time. As proposed by Das Gupta et al., the decomposition analysis helps to assess the potential contributors to disease burden dynamics [18]. This method dissects the whole variation into three components: epidemiological changes, population growth, and ageing. It quantifies the contribution of the above three factors to provide comprehensive information on the dynamics of GBTC burden over the study period. This method allows us to identify whether the increase (or decrease) in cancer burden is mainly due to demographic changes or shifts in the actual risk of disease. This is particularly useful for informing targeted interventions, as it clarifies whether public health efforts should focus on modifying risk factors, addressing demographic transitions, or both.

### 2.7 Cross‐Country Inequality Analysis

To reveal the inequality of GBTC burden across countries, two standard parameters [Slope Index of Inequality (SII) and the Concentration Index] were employed [19]. The SII quantifies burden imbalance by correlating national DALY rates with an SDI‐based scale. An elevated SII value tends to be deeply unbalanced. Additionally, as a relative statistical method, the Concentration Index helps to evaluate the health indicators that were concentrated amongst socio‐economic entities. It is derived from the Lorenz concentration curve. A negative value indicates increased disease burden amongst vulnerable groups, while a positive score means concentration amongst more advantaged groups.

### 2.8 Predictive Analysis

The global GBTC burden was projected through 2040 using the Bayesian APC analysis (BAPC) model with integrated nested Laplace approximation (INLA). Furthermore, the model was considered to perform better than other projection approaches [20]. The R packages ‘INLA’ (version 23.09.09) and ‘BAPC’ (version 0.0.36) were utilised to fit the data. The GBD Population Forecasts 2017–2100 were used for the projection population from 2022 to 2040.

### 2.9 Statistical Analysis

The Joinpoint regression method was used to assess the temporal trends in GBTC burden [21]. The analysis provides information on significant changes in trend segments and estimates the annual percentage change. The average annual percent change (AAPC) was also computed to provide an overall trend estimate from 1990 to 2021. An annual percentage change, or AAPC, with a 95% confidence interval (CI) lower bound > 0 indicates an upward trend, while a CI upper bound < 0 indicates a downward trend. To evaluate correlations between the SDI and the ASR, the Spearman correlation was employed. All statistical processes were performed by R software (version 4.2.3), STATA software (version 18.0), and Joinpoint Statistical Software (version 4.8.0.1). A *p*‐value of < 0.05 was used as statistical significance.

## 3 Results

### 3.1 Global, Regional, and National Burden and Trends of GBTC From 1990 to 2021

**FIGURE 1:**
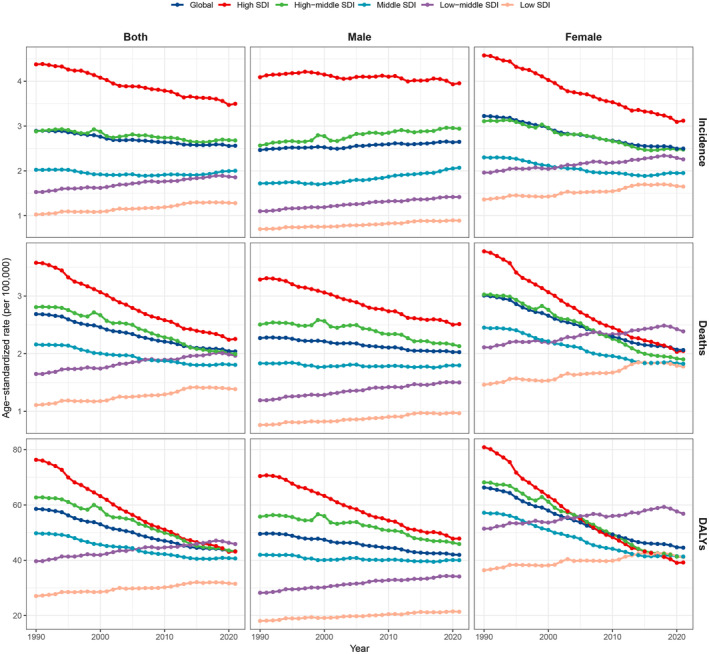
Trends of age‐standardised rates of incidence, mortality, and DALYs of GBTC by gender across different SDI regions. DALYs, disability‐adjusted life years; GBTC, gallbladder, and biliary tract cancer; SDI, socio‐demographic index.

In 2021, the global incidence of GBTC was 216 768 cases (95% uncertainty interval (UI): 181888‐245 238), with 171 961 deaths (95% UI: 142352‐194 238), and 3 732 121 DALYs lost (95% UI: 3102893‐4 316 986) (Table 1). From 1990 to 2021, the global age‐standardised incidence rate (ASIR) decreased slightly (AAPC: −0.39%, 95% CI: −0.50% to −0.28%), along with age‐standardised mortality rate (ASMR) (AAPC: −0.97%, 95% CI: −1.07% to −0.88%), and age‐standardised DALYs rate (ASDR) (AAPC: −0.88%, 95% CI: −0.96% to −0.80%) (Figure 1, Table S1). In terms of sex, it is worth noting that the male ASIR increased slowly (AAPC: 0.229% 95% CI: 0.078% to 0.381%), while the female ASIR significantly declined (AAPC: −0.817% 95% CI: −0.94% to −0.695%).

**TABLE 1:** The global and regional absolute number of GBTC incidence, deaths, and DALYs in 1990 and 2021.

	Incidence		Deaths		DALYs	
	1990 number (95% UI)	2021 number (95% UI)	1990 number (95% UI)	2021 number (95% UI)	1990 number (95% UI)	2021 number (95% UI)
Location						
Global	107 797.8 (96 890.2–117 511.8)	216 768.3 (181 888–245 237.6)	98 683.1 (88 940.5–109 189.4)	171 961.2 (142 351.8–194 238.4)	2 326 089.1 (2 054 497.2–2 582 958.7)	3 732 121.3 (3 102 893.2–431 6985.6)
Sex						
Male	40 915.3 (34 823.8–46 844.7)	100 783.6 (77 371.7–116 395.6)	36 711.4 (30 577.5–43 205.3)	75 947.1 (57 121.9–88 998.8)	911 133 (740 608.1–1 080 564.5)	1 684 804.6 (1 229 526.9–1 996 599.7)
Female	66 882.5 (59 470.7–75 642.1)	115 984.8 (94 592.8–132 850.2)	61 971.7 (54 706.6–71 113)	96 014.1 (77 016–111 372.4)	1 414 956 (1 232 933–1 649 431.4)	2 047 316.7 (1 624 444.9–2 400 736.8)
Region						
Central Asia	488.9 (434.8–566.8)	651.6 (576.1–735.6)	504.2 (447.7–584.8)	661.8 (584.9–748.2)	13 019.4 (11 533.3–15 009.7)	16 881.4 (14 848.6–19 118)
Central Europe	6542.2 (6149.4–6817.1)	6407.2 (5829.7–6991.4)	6729.7 (6351.5–7005.8)	5980.3 (5462.9–6514.4)	150 655.4 (141 792.4–156 408.9)	120 977.8 (111 327.2–131 747.7)
Eastern Europe	4610.7 (4314–4945.3)	6529.9 (6045.3–7030.6)	4183.5 (3907.7–4495.2)	4716.1 (4345.1–5100.3)	100 948.9 (94 310.3–108 581.8)	103 828.7 (95 543–112 484.7)
Australasia	793.6 (746–834.4)	1839.1 (1612.4–1980.9)	412 (387.6–431.3)	627.8 (550.8–675)	8769.7 (8355–9133.7)	11 915.1 (10 805.7–12 669.9)
High‐income Asia Pacific	18 528.2 (17 051.1–19 642.8)	36 719.6 (30 696.6–41 221.2)	16 276.7 (14 939.8–17 289.1)	27 837.4 (23 021.5–31 233.9)	348 590.4 (319 166.4–370 725.3)	422 749 (365 402.3–468 091.5)
High‐income North America	9069.2 (8412.7–9440.2)	14 132.4 (12 808.9–14 899.1)	5047 (4659.4–5257.8)	5981.4 (5372.8–6322.9)	102 978.5 (97 536.6–106 419)	122 252.1 (113 814.8–127 915.3)
Southern Latin America	3940.8 (3758.4–4107)	4439.9 (4080.1–4705.6)	4028.4 (3835.3–4199.5)	3992.8 (3672.5–4230.5)	94 083.1 (90 391.1–97 743)	87 893.3 (82 463.2–92 630.6)
Western Europe	21 787.9 (20 288–22 707.1)	25 453.6 (22 479.8–27 379.5)	18 469.5 (17 094.5–19 266.8)	16 112 (14 089.1–17 438.8)	364 377.3 (343 527.7–377 328.5)	283 997.7 (257 157–302 792.3)
Andean Latin America	978.3 (750.4–1146.1)	2327.9 (1771.8–3038.1)	1038.3 (800.1–1220.2)	2328.6 (1773.2–3009.9)	25 935.9 (19 621.9–30 356.2)	53 934.4 (41 069.9–70 389.4)
Caribbean	418.3 (366.8–460.7)	540.3 (470.9–620.5)	425.7 (371.4–469.5)	527.4 (458.2–609)	10 241 (8792.2–11 448.8)	12 428.4 (10 616–14 665.1)
Central Latin America	3406.4 (3298.4–3483.4)	5362.9 (4777.2–5957)	3561.5 (3439.2–3644)	5318.3 (4742.1–5887.7)	89 553 (87 214.4–91 360.4)	127 353.3 (113 502.6–142 341.7)
Tropical Latin America	2732.3 (2591–2830.8)	5834.2 (5381.8–6124.6)	2844.3 (2687.9–2950.6)	5866 (5375.2–6168.7)	72 286.1 (69 330.4–74 609.6)	139 553.8 (131 318.8–145 404.3)
North Africa and Middle East	2205.6 (1783.6–2887.3)	5941.6 (4364.3–7299.9)	2273.5 (1852.5–2988.9)	5586 (4101.3–6845.8)	59 856.9 (47 193.2–77 228.5)	139 588.4 (10 0801.9–168 471.3)
South Asia	9355.2 (7565–13 437.8)	32 822.9 (22 750.2–38 788.4)	9690.9 (7833.2–13 941.9)	33 697.8 (23 308.4–39 892.2)	272 670.8 (216 272.3–389 257.7)	865 381.8 (591 762.4–1 024 304.3)
East Asia	17 801 (13 689.8–22 560.1)	53 483.3 (37 557.4–68 788.7)	17 920.8 (13 851.3–22 907.3)	39 221 (28 021.2–51 034.3)	470 770.1 (358 405.9–604 066.3)	889 305.9 (634 707.7–1 160 128)
Oceania	17.6 (10.3–23.6)	37.2 (24.6–48.6)	17.9 (10.6–23.8)	37.7 (25.1–48.9)	520.3 (297.5–702.7)	1071.9 (701.8–1417.6)
Southeast Asia	4093.1 (2956.2–5132.5)	12 035.1 (8763–15 157.1)	4182.6 (3055.5–5270.9)	11 163.5 (8220.2–14 176.4)	111 514.4 (80 436.6–139 488.5)	272 265.7 (200 969.9–343 849.8)
Central Sub‐Saharan Africa	63.9 (43.7–92.1)	165.1 (109.9–235.1)	66.4 (45.5–96.4)	170.7 (114.1–244)	1857.2 (1249.4–2750.4)	4741.8 (3117.4–6742)
Eastern Sub‐Saharan Africa	747.6 (500.1–1013.7)	1482.5 (1042–1969.8)	783.2 (526.2–1061.4)	1555.3 (1091.8–2067.4)	21 452.8 (14 043.4–29 307.3)	40 937.7 (28 411.3–55 140.9)
Southern Sub‐Saharan Africa	178.7 (132.9–237)	464.5 (325.8–546.9)	186.8 (138.8–247.3)	476.6 (336.1–559.6)	4926.8 (3731.4–6532.5)	12 409.2 (8739.3–14 683.3)
Western Sub‐Saharan Africa	38.4 (30.3–50.5)	97.4 (62.8–119.4)	40.4 (32.4–53.9)	102.6 (66.1–125.4)	1081.4 (818–1337.3)	2654.1 (1667.9–3319)
Socio‐demographic index						
High SDI	49 139.5 (45 627.5–51 242.9)	78 916.7 (69 102.3–86 388.9)	40 256.4 (37 220.7–42 084.5)	52 694.9 (45 511.5–58 047.5)	840 674.2 (781 767.5–877 665.7)	910 136.9 (801 570.4–991 393.6)
High‐middle SDI	27 573.3 (23 969.3–29 651.9)	52 989.4 (40 578.6–61 161.2)	26 375.4 (22 867.7–28 416.7)	39 462.7 (30 763.5–45 342.8)	627 565 (533 882–680 182.5)	855 457.8 (651 942.2–983 362.1)
Middle SDI	19 716.1 (17 100.5–25 134.1)	52 742.2 (42 748.2–68 345.9)	20 216.8 (17 589.4–25 854.9)	466 25.1 (37 882.5–59 063.9)	533 713.7 (457 797.4–673 936.5)	1 116 081.8 (907 042.2–1 399 227.2)
Low‐middle SDI	8973.4 (7687.7–12 693.2)	25 879.1 (20 359.2–32 117.2)	9345.8 (8005.6–13 346.3)	26 697.9 (20 967–33 263.1)	255 585.4 (215 067–361 906.6)	681 874.3 (529 871.5–840 521.1)
Low SDI	2249.6 (1774.7–3121.8)	6081.3 (4206.1–7565.7)	2342.3 (1854.1–3262)	6337.3 (4386.3–7847.2)	65 245.8 (50 716.8–90 203.8)	165 538.5 (115 083.3–207 610.5)

Abbreviations: DALYs, disability‐adjusted life years; GBTC, gallbladder and biliary tract cancer; SDI, socio‐demographic index; UI, uncertainty interval.

**FIGURE 2:**
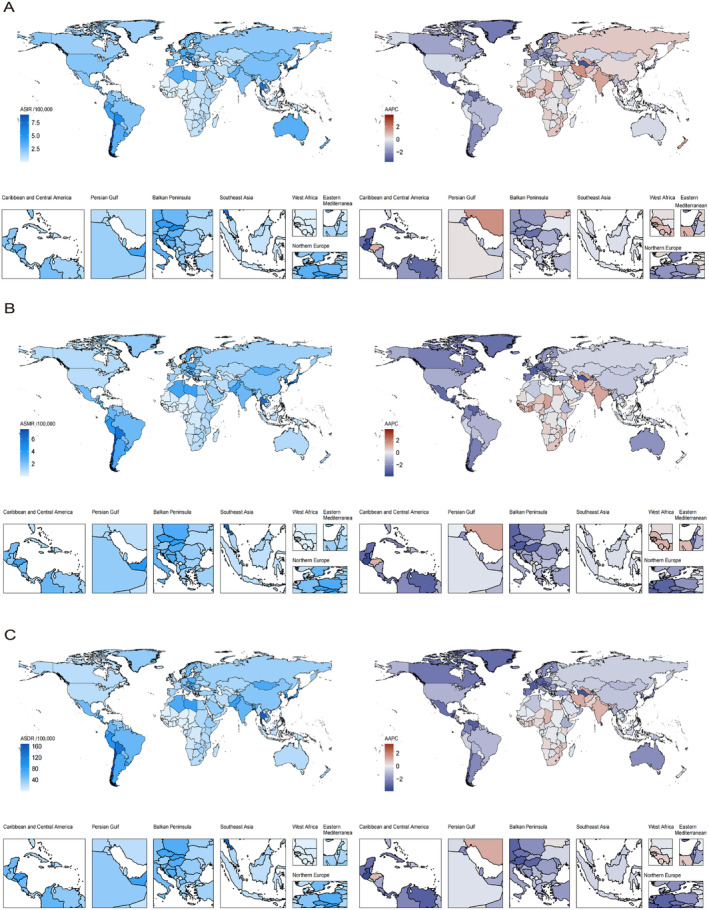
The geographical distribution of ASR and AAPC of GBTC burden in 204 countries and territories in 2021. (A) The ASIR (left) in 2021 and its trend (right) from 1990 to 2021. (B) The ASMR (left) in 2021 and its trend (right) from 1990 to 2021. (C) The ASDR (left) in 2021 and its trend (right) from 1990 to 2021. AAPC, average annual percentage change; AAPC, average annual percent change; ASR, age‐standardised rate; ASDR, age‐standardised disability‐adjusted life years rate; ASIR, age‐standardised incidence rate; ASMR, age‐standardised mortality rate; GBTC, gallbladder and biliary tract cancer.

Regionally, the high‐income Asia Pacific and southern and Andean Latin America exhibited higher ASIR, ASMR, and ASDR (Table 2, Figure 2, Figure S1A–C). In contrast, Western Sub‐Saharan Africa had the lowest values for incidence, deaths, and DALYs. Notably, South Asia and Southern Sub‐Saharan Africa have seen continuous increases in ASIR, ASMR, and ASDR over the past 32 years.

**TABLE 2:** Global and regional age‐standardised rate of GBTC incidence, death, and DALYs in 1990 and 2021.

	Incidence		Death		DALYs	
	1990 rate (95% UI)	2021 rate (95% UI)	1990 rate (95% UI)	2021 rate (95% UI)	1990 rate (95% UI)	2021 rate (95% UI)
Location						
Global	2.892 (2.585–3.147)	2.56 (2.158–2.892)	2.687 (2.389–2.971)	2.04 (1.696–2.294)	58.581 (52.214–64.885)	43.205 (36.014–49.882)
Sex						
Male	2.465 (2.134–2.804)	2.648 (2.058–3.045)	2.271 (1.925–2.648)	2.027 (1.54–2.362)	49.551 (40.88–58.547)	41.955 (30.945–49.562)
Female	3.224 (2.851–3.639)	2.497 (2.033–2.86)	3.009 (2.661–3.445)	2.063 (1.654–2.393)	66.308 (57.94–77.19)	44.555 (35.347–52.311)
Region						
Central Asia	1.064 (0.944–1.237)	0.828 (0.736–0.936)	1.113 (0.987–1.294)	0.861 (0.763–0.975)	27.201 (24.091–31.429)	19.977 (17.634–22.616)
Central Europe	4.426 (4.167–4.619)	2.798 (2.545–3.05)	4.616 (4.358–4.812)	2.582 (2.36–2.814)	99.88 (94.07–103.748)	55.325 (50.976–60.111)
Eastern Europe	1.648 (1.538–1.766)	1.84 (1.704–1.98)	1.507 (1.405–1.619)	1.319 (1.215–1.425)	35.602 (33.199–38.302)	29.816 (27.371–32.28)
Australasia	3.362 (3.155–3.533)	3.352 (2.981–3.596)	1.758 (1.65–1.845)	1.095 (0.974–1.173)	37.483 (35.715–38.974)	22.803 (20.957–24.134)
High‐income Asia Pacific	9.485 (8.672–10.081)	6.569 (5.625–7.327)	8.408 (7.675–8.943)	4.799 (4.134–5.339)	172.424 (158.073–183.498)	86.557 (75.722–96.266)
High‐income North America	2.525 (2.351–2.625)	2.142 (1.957–2.25)	1.391 (1.288–1.447)	0.88 (0.798–0.927)	29.608 (28.14–30.554)	19.258 (18.063–20.096)
Southern Latin America	8.625 (8.202–8.989)	5.059 (4.669–5.357)	8.914 (8.444–9.306)	4.509 (4.155–4.779)	202.484 (194.441–210.255)	102.435 (96.349–107.955)
Western Europe	3.651 (3.412–3.797)	2.566 (2.315–2.733)	3.071 (2.848–3.2)	1.549 (1.386–1.663)	63.36 (59.946–65.553)	31.055 (28.613–32.811)
Andean Latin America	4.941 (3.816–5.789)	3.993 (3.04–5.198)	5.343 (4.144–6.281)	4.026 (3.068–5.188)	123.791 (93.981–144.881)	90.54 (68.886–118.022)
Caribbean	1.636 (1.437–1.799)	1.002 (0.874–1.153)	1.686 (1.478–1.859)	0.977 (0.848–1.128)	38.966 (33.54–43.535)	23.114 (19.732–27.286)
Central Latin America	4.273 (4.119–4.378)	2.16 (1.927–2.396)	4.583 (4.405–4.703)	2.163 (1.931–2.392)	104.658 (101.675–106.931)	50.013 (44.62–55.869)
Tropical Latin America	3.142 (2.936–3.265)	2.28 (2.096–2.397)	3.376 (3.147–3.517)	2.31 (2.109–2.431)	76.797 (73.265–79.504)	53.584 (50.274–55.875)
North Africa and Middle East	1.4 (1.143–1.847)	1.398 (1.034–1.735)	1.501 (1.226–1.991)	1.365 (1.01–1.693)	34.262 (27.541–44.695)	29.816 (21.756–36.224)
South Asia	1.672 (1.364–2.403)	2.276 (1.586–2.708)	1.799 (1.467–2.615)	2.398 (1.674–2.852)	44.104 (35.446–63.437)	56.519 (38.801–66.92)
East Asia	2.196 (1.695–2.786)	2.488 (1.737–3.186)	2.314 (1.788–2.957)	1.851 (1.311–2.398)	52.648 (40.406–66.947)	40.45 (28.785–52.444)
Oceania	0.636 (0.389–0.824)	0.522 (0.352–0.66)	0.683 (0.423–0.873)	0.556 (0.376–0.704)	16.386 (9.682–21.699)	13.2 (8.772–17.193)
Southeast Asia	1.683 (1.223–2.115)	1.933 (1.407–2.429)	1.785 (1.304–2.261)	1.853 (1.365–2.347)	41.941 (30.481–52.647)	40.727 (30.049–51.364)
Central Sub‐Saharan Africa	0.304 (0.216–0.425)	0.326 (0.217–0.459)	0.333 (0.236–0.467)	0.356 (0.236–0.505)	7.832 (5.386–11.291)	8.169 (5.458–11.663)
Eastern Sub‐Saharan Africa	1.042 (0.711–1.402)	0.953 (0.67–1.261)	1.132 (0.776–1.518)	1.044 (0.734–1.386)	27.109 (18.09–36.781)	23.327 (16.368–31.014)
Southern Sub‐Saharan Africa	0.685 (0.505–0.905)	0.839 (0.586–0.985)	0.74 (0.544–0.967)	0.892 (0.619–1.04)	17.304 (12.956–23.068)	20.588 (14.508–24.264)
Western Sub‐Saharan Africa	0.045 (0.037–0.062)	0.054 (0.035–0.066)	0.049 (0.04–0.068)	0.06 (0.038–0.072)	1.164 (0.922–1.51)	1.3 (0.839–1.595)
Socio‐demographic index						
High SDI	4.377 (4.059–4.567)	3.497 (3.11–3.804)	3.577 (3.308–3.742)	2.256 (1.972–2.477)	76.338 (70.956–79.757)	43.161 (38.506–46.766)
High‐middle SDI	2.881 (2.504–3.093)	2.679 (2.049–3.091)	2.808 (2.434–3.02)	1.995 (1.552–2.293)	62.714 (53.603–67.857)	43.273 (32.904–49.755)
Middle SDI	2.024 (1.767–2.571)	2.002 (1.629–2.596)	2.16 (1.893–2.753)	1.807 (1.468–2.293)	49.774 (43.055–63.313)	40.657 (32.877–51.042)
Low‐middle SDI	1.527 (1.312–2.179)	1.856 (1.473–2.311)	1.649 (1.413–2.356)	1.966 (1.557–2.458)	39.664 (33.709–56.295)	45.867 (35.744–56.908)
Low SDI	1.026 (0.82–1.435)	1.28 (0.878–1.565)	1.109 (0.89–1.556)	1.386 (0.951–1.688)	27.057 (21.297–37.664)	31.487 (21.788–39.139)

Abbreviations: ASR, age‐standardised rate (per 10^5^ population); DALYs, disability‐adjusted life years; GBTC, gallbladder and biliary tract cancer; SDI, socio‐demographic index; UI, uncertainty interval.

Nationally, Chile suffered the highest ASIR (9.01 per 10^5^ population, 95% UI: 8.22–9.77), ASMR (7.57 per 10^5^ population; 95% UI: 6.86–8.21), and ASDR (168.03 per 10^5^ population; 95% UI: 155.22–180.70) (Tables S2–S4, Figure S1D–F). Cabo Verde showed the fastest rise in ASIR (AAPC: 3.88%, 95% CI: 3.62 to 4.13%), ASMR (AAPC: 3.81%, 95% CI: 3.56 to 4.07%), and ASDR (AAPC: 3.42%, 95% CI: 3.17 to 3.68%). Conversely, Sri Lanka had a notable annual decline in ASIR (AAPC: −3.43%, 95% CI: −4.33 to −2.53%), ASMR (AAPC: −3.87%, 95% CI: −4.77 to −2.97%), and ASDR (AAPC: −3.87%, 95% CI: −4.71 to −3.04%).

### 3.2 GBTC Burden and Trend Stratified by SDI Quantiles From 1990 to 2021

Amongst the five SDI regions, the high SDI region revealed the highest ASIR (3.50 per 10^5^ population; 95% UI: 3.11–3.80) and ASMR (2.26 per 10^5^ population; 95% UI: 1.97–2.48), while the highest ASDR [45.87 per 10^5^ population (95% UI, 35.74–56.91)] was identified in the low‐middle SDI region (Table 2). Unexpectedly, the low SDI regions displayed a gradual rise in ASIR (AAPC: 0.75%, 95% CI: 0.56%–0.95%), ASMR (AAPC: 0.75%, 95% CI: 0.55%–0.96%), and ASDR (AAPC: 0.52%, 95% CI: 0.35%–0.68%), even though the absolute number of GBTC incidence, mortality, and DALYs in the region was relatively low (Table S1).

### 3.3 Age–Period–Cohort Effects on GBTC Incidence

**FIGURE 3:**
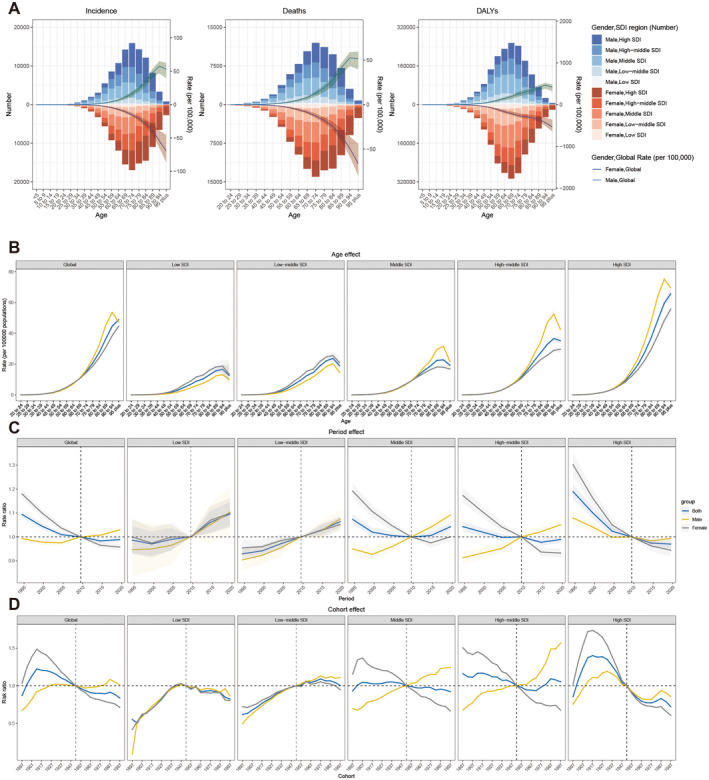
(A) The number and age‐specific rates for GBTC incidence, deaths, and DALYs stratified by gender across different SDI regions in 2021.(B) The age effects are shown by the fitted longitudinal age curves on GBTC incidence rate across different SDI regions. (C) The period effects on GBTC incidence rate from 1992 to 2021 across different SDI regions; (D) The cohort effects on GBTC incidence rate from 1992 to 2021 across different SDI regions. DALYs, disability‐adjusted life years; GBTC, gallbladder and biliary tract cancer; SDI, socio‐demographic index.

The age‐specific rates of incidence, deaths, and DALYs of GBTC increased with age, peaking approximately at 90–94 years old (Figure 3A,B). The peak absolute number of DALYs happened around 5 years earlier than that of incidence and death. In addition, the incidences of worldwide GBTC cases tended to rise with age, demonstrating the same pattern across all SDI regions, according to the findings of longitudinal age curves (Figure 3B, Table S5). Except for low SDI and low‐middle SDI regions, the ASIR of males in other regions was higher than females (Figure 3B).

Regarding the period effect, the past 30 years have seen a negative trend globally, except for low and low‐middle SDI regions. Notably, the overall period curve tended to climb after 2015. Unlike high SDI regions, the period trend in males has been steadily rising in all other SDI regions since 2000. Before 2015, the period trend of females in the middle, middle‐high, and high SDI regions showed a declining pattern. However, in the middle SDI area, this trend began to rise from 2015 (Figure 3C, Table S6).

There existed a notable regional unbalance of cohort effect on GBTC incidence in the past 30 years (Figure 3D, Table S7). Globally, the cohort effect typically increased and then decreased, peaking between 1907 and 1917. There was a subsequent peak noted in the birth period of 1982–1991. The differences in cohort effects between genders were less noticeable in the low and low‐middle SDI regions. Besides, there was a similar trend of early rise followed by a decrease in all regions. In middle and high‐middle SDI regions, the birth cohort effect on GBTC incidence in males gradually increased. Differently, in the high SDI region, the birth cohort effect amongst males fluctuated, initially surging between 1897 and 1927, followed by a precipitous drop between 1937 and 1977, and culminating in a minor peak thereafter.

**FIGURE 4:**
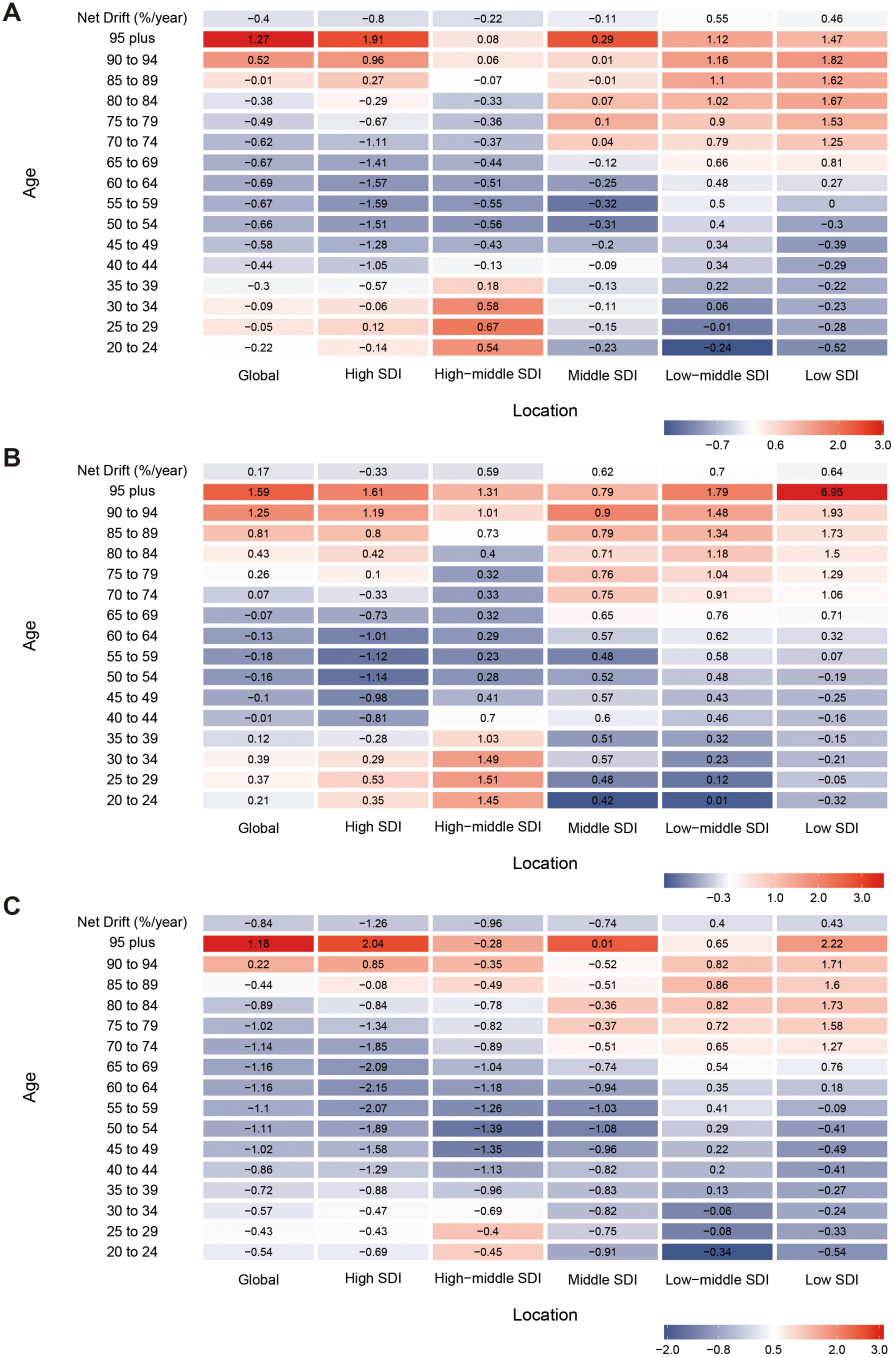
Net and Local drifts for GBTC incidence in global and five SDI regions by (A) both, (B) male, and (C) female. DALYs, disability‐adjusted life years; GBTC, gallbladder, and biliary tract cancer; SDI, socio‐demographic index.

The results of net drifts emphasised the reduction trend of global ASIR in the total and females (Figure 4). The local drifts revealed a shift of global ASIR exiting around 90 years old (Figure 4A,C). Among the five SDI regions, low‐middle and low SDI regions showed an increased GBTC incidence, with an overall net drift > 0. Interestingly, all age subgroups of males in high‐middle, middle, and low‐middle SDI regions indicated an increasing trend (Figure 4B). Notably, the local drifts in high‐middle SDI regions reported an elevated early‐onset GBTC incidence amongst males.

### 3.4 Decomposition Analysis of GBTC Burden Across SDI Regions

**FIGURE 5:**
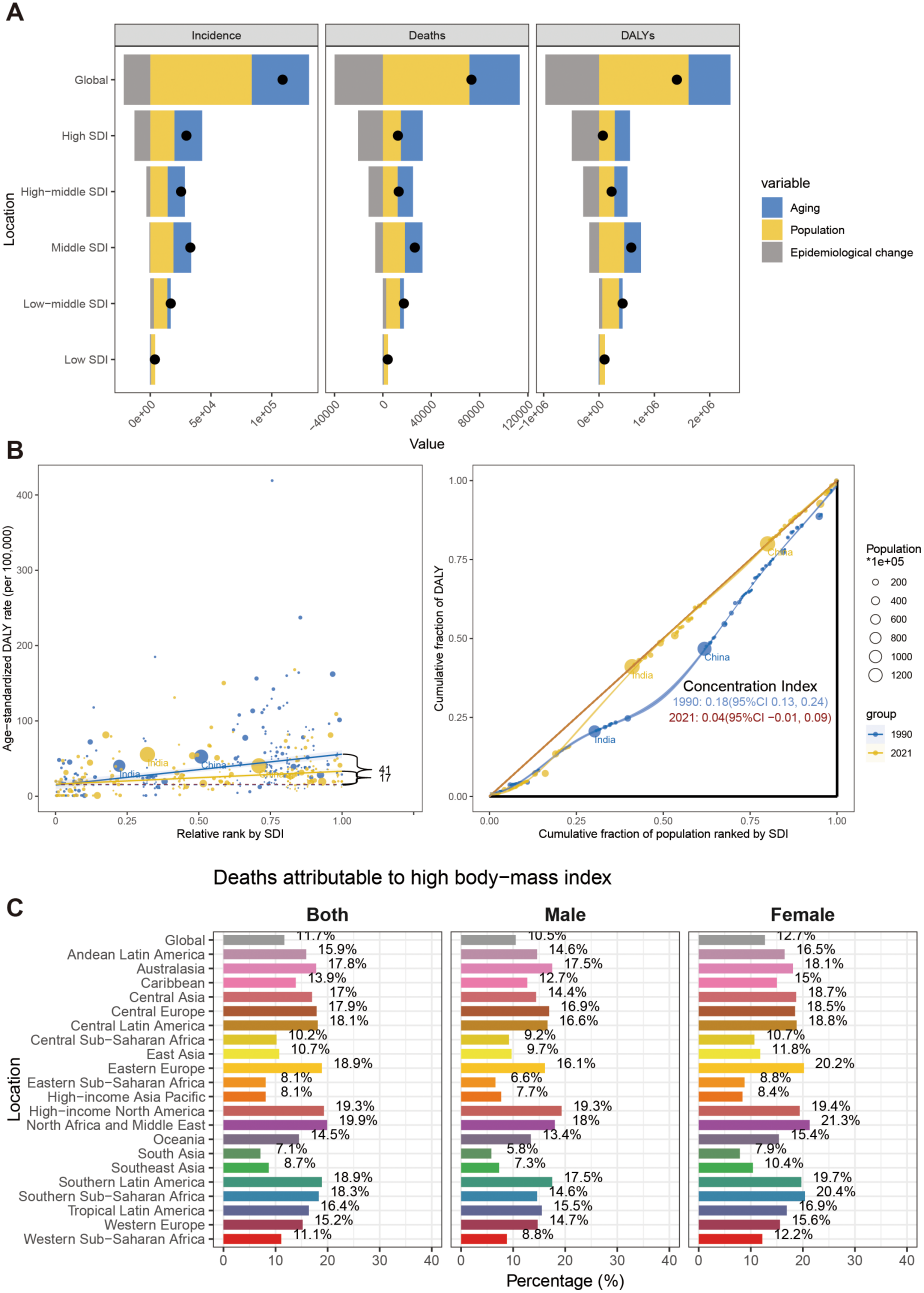
(A) Decomposition analysis for GBTC incidence, deaths, and DALYs. (B) health inequality assessment of GBTC‐related DALYs across SDI regions. (C) GBTC‐related DALYs risk factor attributable to high BMI. BMI, body mass index; DALYs, disability‐adjusted life years; GBTC, gallbladder and biliary tract cancer; SDI, socio‐demographic index.

Decomposition analysis decomposes the burden changes of GBTC into three parts: epidemiological changes, population growth, and ageing (Figure 5A, Table S8). Between 1990 and 2021, population growth was the primary contributor to the increase in GBTC incidence (76.7%), deaths (97.6%), and DALYs (115.1%) globally. Across all SDI regions, population growth played a more significant role in the GBTC burden than ageing and epidemiological changes. Besides, the epidemiological change mainly exerted a negative impact on the GBTC burden in most SDI regions. However, in the low SDI region, population ageing was negatively associated with the GBTC burden, while the epidemiological shift showed a positive association.

### 3.5 Inequality Analysis of GBTC DALYs

Significant absolute and relative disparities in the SDI‐related burden of GBTC were found in the analysis of 204 countries and territories, with an apparent decrease in these disparities from 1990 to 2021 (Figure 5B, Table S9). The SII for GBTC‐associated ASDR observed an obvious drop, decreasing from 41.01 (95% CI: 30.61–51.42) to 17.15 (95% CI: 10.53–23.77), and the concentration index declined from 0.18 (95% CI: 0.13–0.24) to 0.04 (95% CI: −0.01 to 0.09). This implies that over this time, there was a reduction in the disparity in the burden of GBTC between high‐ and low‐income nations.

### 3.6 Risk Factor for GBTC

An elevated body mass index (BMI) contributed to GBTC‐related death and DALYs in GBD 2021. There were 12.1% of DALYs and 11.7% of deaths due to GBTC associated with an elevated BMI in 2021 globally (Figure 5C, Figure S3). The impact of high BMI on GBTC varied notably across regions. The greatest effect of high BMI for DALYs was observed in high−income North America (total: 20.1%; male: 20%; female: 20.1%) and North Africa and the Middle East (total: 20.5%; male: 18.6%; female: 21.9%). In contrast, the lowest impact was seen in South Asia (total: 7.6%; male: 6.1%; female: 8.3%). A similar pattern could be observed in GBTC‐related death. Besides, more than half of the regions reported a higher proportion of GBTC‐related deaths and DALYs attributable to high BMI compared to the global average.

### 3.7 Projection of GBTC Burden up to 2040

**FIGURE 6:**
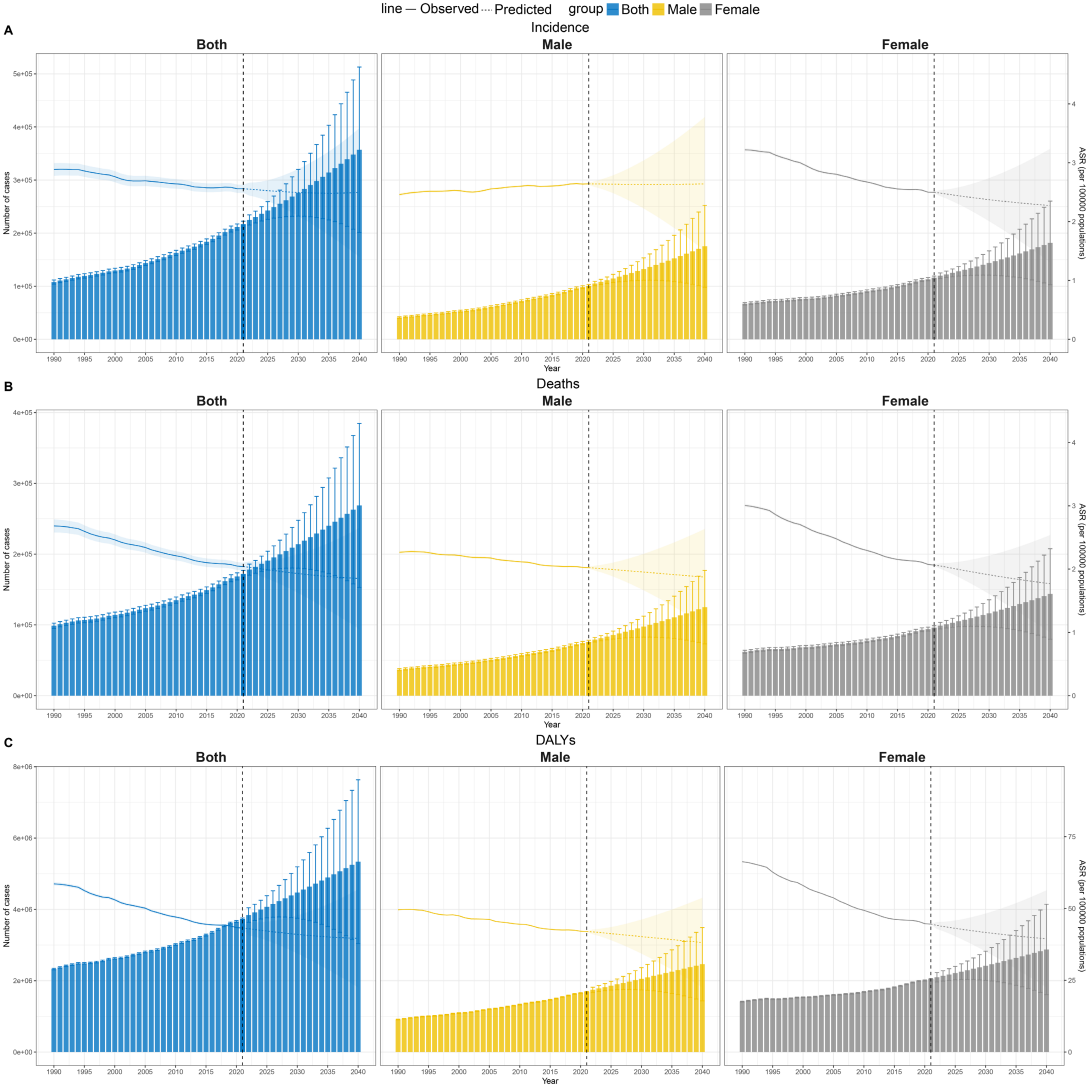
Projections for GBTC burden up to 2040 stratified by gender; (A) incidence, (B) mortality, and (C) DALYs. DALYs, disability‐adjusted life years; GBTC, gallbladder and biliary tract caner.

It is expected that the global ASR of GBTC will slowly decrease from 2021 to 2040, with different trends amongst males and females (Figure 6). The ASIR of global GBTC will decline slowly, from 2.56 (2.158–2.892) in 2021 to 2.495 (1.404–3.587) in 2040. The ASIR of males is expected to remain relatively stable, from 2.648 (2.058–3.045) in 2021 to 2.64 (1.504–3.775) in 2040, while females will experience a more obvious decline, from 2.497 (2.033–2.86) in 2021 to 2.274 (1.311–3.237) in 2040 (Table S10). Both ASMR and ASDR are slowly declining, with both males and females maintaining a downward trend. It is expected that ASMR will decrease from 2.04 (1.696–2.294) in 2021 to 1.851 (1.052–2.651) in 2040 (Table S11), and ASDR will decrease from 43.205 (36.014–49.882) in 2021 to 39.574 (22.527–56.622) in 2040 (Table S12). This indicates that the ASR of GBTC will decrease in the future, but it is still necessary to pay attention to the male incidence rate.

## 4 Discussion

Applying decomposition, inequality, and APC analysis to the latest publicly accessible data from the GBD 2021 study, we assessed the global, regional, and national burden of GBTC and the temporal trends from 1990 to 2021. Over the past 32 years, we observed a substantial rise (> 60%) in the total number of GBTC incident cases, deaths, and DALYs. This increasing number of GBTC cases corresponded with changes in the population composition, such as ageing and population growth, and the global geographical redistribution of risk factors for GBTC [3, 15, 21, 22]. Fortunately, the ASIR, ASMR, and ASDR of GBTC decreased at a slow rate globally. However, disparities in disease burden stratified by regions and gender were detected. Therefore, the declining trend of ASR that has been seen does not indicate that it could cause us to relax our efforts to further prevent or minimise it. The climbing incidence in low and low‐middle SDI regions and the persistent burden amongst males highlight the urgent need for targeted interventions.

Across different SDI regions, there was a consistent downward trend of ASIR, ASMR, and ASDR of GBTC in high and middle‐high SDI regions, but in the low‐middle and low SDI regions, there was an opposite trend. These findings highlighted the confounding impact caused by socio‐demographic changes on the burden of particular diseases across countries [23, 24, 25]. Individuals living in economically developed areas are more likely to have received better education, to prioritise their health, and to have easier access to healthcare [26, 27]. For instance, cholecystectomy and choledochoscopy procedures are more common in middle‐income to high‐income countries for patients with gallstones, effectively minimising the risk of developing carcinoma in the biliary epithelium [28]. Notably, the emergence and progression of tumours are multifaceted outcomes of various potential carcinogenic factors [29]. Despite significant progress in medical preventative and treatment methods, the overall incident cases of GBTC keep rising, mainly due to factors including obesity, smoking, heavy drinking, and hereditary susceptibility [30, 31]. While numerous epidemiological studies have indicated that obesity may be linked to an increased risk of GBTC, this correlation could be confined to Western nations [32, 33, 34]. Similarly, a clear geographical pattern of GBTC disease burden was detected. Although the GBTC‐related incidences, mortality, and DALYs rates of the southern African maintained a comparatively low level after age adjustment, the accelerated rise of ASR was what complicated matters. In the ensuing decades, this would exacerbate the financial and medical costs due to GBTC. Algeria and Libya, with ASIR of 3.23 and 3.60 per 10^5^ population, respectively, experienced the greatest latent burden of GBTC amongst all African nations.

Several malignancies show distinct gender predispositions due to different sex‐preferred carcinogenic mechanisms [5]. It is reported that females display a strong association with the formation of gallstones, especially during the reproductive period [35]. They are nearly twice as susceptible to gallstones compared to males [35]. Due to the high risk of cholelithiasis in women, the greatest frequency of gallbladder cancer was reported amongst females aged over 65 years old [31, 36]. Besides, especially those individuals in developed countries and regions, often suffer from obesity caused by high‐calorie diets, less physical activity, and physical stress [37]. The rise in obesity rate undoubtedly worsens the heavy medical and economic burden of all countries [38]. Worldwide, the prevalence of obesity in females was higher than in males due to numerous gender‐specific mechanisms, including hormones, gut microbiota, and genetic susceptibility [39, 40, 41]. The higher prevalence of obesity in females further promotes an increase in the number of GBTC occurrences and aggravates the burden. Based on the latest 2021 GBD data, we noted that the total number of GBTC incidences in females persistently exceeded that of males. However, more importantly, we found that the ASIR of GBTC in males surpassed females in post‐70 years of age worldwide and then peaked at 90–94 years. Across different SDI regions, we found that when ageing, the ASIR of males in middle, middle‐high, and high SDI regions markedly surpassed that of the females. The potential burden of GBTC in men seems to deteriorate with the development of the economy. In contrast, the ASIR of GBTC kept traditional sexual patterns in low‐middle and low SDI regions. Overweight and obesity often come along with metabolic disorders in multiple organs. Metabolic dysfunction‐associated steatotic liver disease (MASLD) is the main feature of obesity‐related metabolic syndrome [37]. Lee et al. reported that the prevalence of MASLD varied by gender [42]. Males tend to be more susceptible to MASLD than females, especially in the obese population. Numerous epidemiological studies and meta‐analyses revealed the causal association between MASLD and GBTC [43, 44]. This, to some extent, explained why the increase in ASIR amongst males in the developed SDI regions was higher than that amongst females. In addition, future predictions revealed that the ASIR of men is expected to remain relatively stable while females will experience a more significant decline. These findings emphasise the need for forward‐thinking cancer prevention efforts specifically targeting males.

Biological events, demographic transitions, and social instability, generations born in different eras could exert distinct birth cohort effects [45]. In our study, we detected that those born before the 1917s suffered an increasing cohort effect of GBTC worldwide. With the 1917 year as the watershed, the birth cohort effect exhibits a decreasing trend for individuals born in the following periods. In addition to population ageing, early birth subsets often faced a shortage of medical equipment and socio‐economic turbulence that prevented them from getting timely intervention measures for hazardous factors of GBTC such as gallstones and MASLD. The decline in the birth cohort effect post‐1917s endorsed the success of malignant prevention and the improvement of medical conditions. Specifically, the predisposition of gallbladder cancer by cholelithiasis has been identified since 1861 [46]. However, only with the development of the laparoscopic technique did the standard method of cholecystectomy for gallstones come to widely popularise. In 1970, the incidence of gallstones in Chile was 3 to 4 times that of the prevalence of cholecystectomy (4.8 per 10^3^ population) [47, 48]. Fortunately, with the acceptability of cholecystectomy and the development of preventive medicine globally, the ASIR, ASMR, and ASDR of GBTC in Chile declined by more than 2% annually compared to 1990.

We further discussed the underlying roles of demographic shifts in GBTC burden by performing a decomposition analysis. The results revealed an unavoidable fact that the demographic changes (population ageing and expansion) could largely offset the potential effect of epidemiological evolution. The exceeding pace of population growth in developing countries was attributable to the aggravation of the global GBTC burden. Especially in low SDI regions, the positive effect of epidemiological change increased the disease burden to some extent, while the population expansion slowed down the pace of population ageing which exerted the opposite impact. Hence, the above fact emphasised the challenges of the dynamics of social population composition and epidemiology. Demographic evidence supported that most countries were encountering the puzzle of accelerated population ageing [49]. Correspondingly, we found that the effect of ageing increased with socio‐economic development level on the disease burden. Notably, the results also reported a positive ageing trend on GBTC burden in middle‐low SDI regions, highlighting the need for attention to population ageing in underdeveloped areas. Additionally, the potential impacts of population expansion would continue to persist and have long‐term effects across all SDI regions. Moreover, the global slope index of healthy inequality decreased from 41.01 in 1990 to 17.15 in 2021, which meant an alleviation of inequality of GBTC burden. However, the problem of GBTC burden inequality remained particularly prominent in regions such as southern Latin America and South Asia. Benefiting from the national policy ‘Healthy China 2030’, the inequality of this disease burden in China was eased partly [50]. In contrast, the obstacle of disease inequality was more pronounced in India during the same period. The faster population growth, severe environmental pollution, imbalance in medical resources, and urban development orientation were attributable to the increased disease burden and imbalance in India [51, 52]. Therefore, it is essential and urgent to develop appropriate disease prevention and intervention measures based on regional demographic and epidemiological changes to effectively reduce the GBTC burden.

Several limitations exist in this study. Firstly, the quality of the used data in our study largely depended on the collective process of primary data. Although scientific and reasonable statistical methods were employed, the accuracy of the conclusion in this study would be unavoidably confounded by misdiagnosis or other possible human errors in some areas. Secondly, the occurrence and progression of GBTC are multifactorial; limited by the available data from the GBD database, we did not further evaluate the potential impact of other possible carcinogenic variables on the burden of GBTC. The potential confounding effects of various metabolic risk factors (e.g., smoking and alcohol consumption) affect the attribution of high BMI factors [53, 54]. Thirdly, due to the nature of this epidemiological research method, the conclusion we made from the APC analysis was not suitable for individuals. Lastly, this study did not assess the burden of intrahepatic cholangiocarcinoma due to a lack of pertinent data. Furthermore, GBD estimation lacks stratification by anatomical location, making it impossible to further analyse the burden of GBTC based on anatomical location.

The potential of this research lies in its ability to inform targeted public health strategies by identifying high‐risk populations, particularly in low‐ and low‐middle SDI regions with rising disease burden. However, significant knowledge gaps remain, especially regarding the environmental, genetic, and lifestyle factors that contribute to these geographic variations. Addressing these gaps will require collaborative efforts to enhance data accuracy, expand cancer registries, and integrate advanced analytics. Looking ahead, advancements in data science and increased investment in cancer screening programs, particularly in under‐resourced areas, could enable earlier detection and better outcomes, ultimately reducing disparities of the burden GBTC in the future.

## 5 Conclusions

In general, the global ASR of GBTC incidence, mortality, and DALYs decreased slightly from 1990 to 2021, with substantial regional, gender, and age disparities. Given the ongoing demographic transitions, the inequitable distribution of the burden of GBTC would remain significant for a long time. Future efforts should focus on reducing socio‐economic inequalities and mitigating risk factors, especially amongst vulnerable populations.

## Author Contributions

Conception and design: Xiaojun Lin, Sen Lei, and Guizhong Huang. Collection and assembly of data: Sen Lei. Data analysis and interpretation: Sen Lei and Guizhong Huang. Manuscript writing: Sen Lei, Guizhong Huang, Xiaohui Li, Pu Xi, and Zehui Yao. Final approval of manuscript: All authors.

## Ethics Statement

The data analysed in this study is publicly available from existing, published Global Burden of Disease (GBD) study from 1990 to 2021. Therefore, informed consent was waived.

## Consent

The authors have nothing to report.

## Conflicts of Interest

The authors declare no conflicts of interest.

## Supporting information


Data S1.

## Data Availability

Data are available on the Global Health Data Exchange GBD website (https://vizhub.healthdata.org/gbd‐results/).
