# Supplementary material for: Global Burden, Trends, and Inequalities of Gallbladder and Biliary Tract Cancer, 1990–2021: A Decomposition and Age–Period–Cohort Analysis
Source: Liver Int. 2025 Jan 1;45(2):e16199. doi: 10.1111/liv.16199 (PMC11688657; doi:10.1111/liv.16199)
Supplement: Supplementary file 1 — Data S1. [file LIV-45-0-s001.docx]

**Global Burden, Trends and Inequality of Gallbladder and Biliary Tract Cancer, 1990-2021: A Decomposition and Age-Period-Cohort Analysis**

Contents

[eMethods 2](#_Toc182315192)

[Supplementary Table 1. Global and regional AAPC for ASR of GBTC incidence, deaths, and DALYs from 1990 to 2021 74](#_Toc182315193)

[Supplementary Table 2. National ASIR of GBTC in 2021, and its temporal trend from 1990 to 2021 77](#_Toc182315194)

[Supplementary Table 3. National ASMR of GBTC in 2021, and its temporal trend from 1990 to 2021 85](#_Toc182315195)

[Supplementary Table 4. National ASDR of GBTC in 2021, and its temporal trend from 1990 to 2021 93](#_Toc182315196)

[Supplementary Table 5. Fitted longitudinal age-specific incidence rates of GBTC in global and five SDI regions from 1992 to 2021. 101](#_Toc182315197)

[Supplementary Table 6. Period rate ratio of GBTC in global and five SDI regions from 1992 to 2021 108](#_Toc182315198)

[Supplementary Table 7. Cohort rate ratio of GBTC in global and five SDI regions from 1992 to 2021 111](#_Toc182315199)

[Supplementary Table 8. Decomposition analysis for global and region GBTC burden from 1990 to 2021 121](#_Toc182315200)

[Supplementary Table 9. Slope index for global and region GBTC-related DALYs in 1990 and 2021 122](#_Toc182315201)

[Supplementary Table 10. Projections of GBTC incidence up to 2040 stratified by sex 125](#_Toc182315202)

[Supplementary Table 11. Projections of GBTC deaths up to 2040 stratified by sex 126](#_Toc182315203)

[Supplementary Table 12. Projections of GBTC DALYs up to 2040 stratified by sex 127](#_Toc182315204)

[Supplementary Figure 1. The trend of ASIR, ASMR, and ASDR by SDI from 1990 to 2021 at the global, regional and national levels. 129](#_Toc182315205)

[Supplementary Figure 2. The GBTC incidence rate between genders across different SDI regions. 131](#_Toc182315206)

[Supplementary Figure 3. Deaths risk factor attributable to high BMI. 132](#_Toc182315207)

# eMethods

**GBD overview**

The GBD 2021 study uses the most recent epidemiological data and improved standard operating procedures to provide an extensive assessment of health loss associated with 369 diseases, injuries, and impairments as well as 88 risk variables, spanning 204 countries and territories[1]. The GBD study estimated the burden of GBTC using advanced modeling approaches. The disease-model-Bayesian meta-regression program, DisMod-MR 2.1, was used to compute incidence and prevalence. To produce reliable estimates, this Bayesian geospatial program combines geospatial data, epidemiological correlations, and a variety of disease factors. The Cause of Death Ensemble modeling (CODEm) framework was used to estimate mortality[1–3]. Vital registration and verbal autopsy data, including those without explicit codes, are incorporated into this technique. Before analysis, the data underwent extensive correction to guarantee accuracy. CODEm improves the accuracy of mortality rate estimates by combining many models. By using these models on the 2021 database, thorough estimates of the burden of GBTC were generated. The methodology takes into consideration variations in research design and methodology among various data sources, guaranteeing precise and consistent estimates of the incidence, prevalence, and mortality of GBTC. DALY estimates for GBD 2021 were derived from 100,983 data sources, 19 of which were used for the first time in 2021. These data sources included 75,459 data sources on non-fatal causes, of which 36,916 contained information on incidence, 22,236 contained information on prevalence, and 45 included information on other epidemiological indicators (such as remission)[1].

**Age-standardized rates**

The age-standardized rates (ASRs) and estimated annual percentage change were used to quantify the GBTC burden trends. It is necessary to standardize the data when comparing several population groups with different age classes or for the same population over time in which the age profiles change. We calculated the ASRs and their associated 95% confidence intervals (95% CI) for DALYs, mortality, and incidence according to the GBD 2021 standard populations, using the following equation:

$ASR=\frac{\sum_{i=1}^{A} a_{i}n_{i}}{\sum_{i=1}^{A} n_{i}}\times100000$,

where $a_{i}$ is the rate in the $i_{th}$ age group and $n_{i}$ is the GBD standard population number in the $i_{th}$ age group.

**Risk Factors**

Six steps are involved in estimating the risk factors for GBD. This process is based on a comparative risk assessment approach[2, 3]. The first step is to identify risk-outcome pairs. Only risk-outcomes that meet the World Cancer Research Fund's criterion of persuasive or credible evidence are included in the estimate of GBD risk factors. The second involves calculating each risk-outcome pair's relative risk (RR) as a function of exposure. The third shows how each risk factor's exposure is distributed by year, location, sex, and age. Finding the theoretical minimum risk exposure level (TMREL) is the fourth step. Estimating the attributable burden and population attributable fraction (PAF) is the fifth step. The PAF is modeled using exposure levels, TMREL, and the RR for each risk-outcome pair. The sixth is estimating the PAF and burden attributable to the combination of risk factors.

**Uncertainty**

We captured and propagated uncertainty through all calculations by sampling 500 values (called draws) for each prevalence, death, YLL, YLD, or DALY estimate and summing draws across age, cause, and location for all intermediate calculations. 95% uncertainty intervals (UIs) were defined by the ordinal 25th and 97.5th draw values[1].

**Socio-demographic Index**

The Socio-demographic Index (SDI) is a composite indicator of background social and economic conditions that influence health outcomes in each location. In short, it is the geometric mean of 0 to 1 indices of total fertility rate (TFR) for those younger than 25 years old (TFU25), mean education for those 15 years old and older (EDU15+), and lag-distributed income (LDI) per capita[1]. According to SDI quintiles, 204 countries and territories were categorized into five groups: low-SDI, low-middle-SDI, middle-SDI, high-middle-SDI, and high-SDI quintiles. The detailed SDI values of five groups as follows:

| Location name | Lower bound | Upper bound |
| --- | --- | --- |
| Low SDI | 0 | 0.466 |
| Low-middle SDI | 0.466 | 0.619 |
| Middle SDI | 0.619 | 0.712 |
| High-middle SDI | 0.712 | 0.810 |
| High SDI | 0.810 | 1 |

The SDI regions and the countries within each SDI regions in 2021 as follows:

| SDI regions | Country | SDI-value in 2021 |
| --- | --- | --- |
| High SDI region |  |  |
|  | Switzerland | 0.934 |
|  | Norway | 0.917 |
|  | Monaco | 0.910 |
|  | Germany | 0.904 |
|  | Denmark | 0.897 |
|  | Netherlands | 0.888 |
|  | San Marino | 0.888 |
|  | Sweden | 0.887 |
|  | Republic of Korea | 0.887 |
|  | Luxembourg | 0.885 |
|  | Taiwan (Province of China) | 0.875 |
|  | Iceland | 0.875 |
|  | Ireland | 0.874 |
|  | Canada | 0.873 |
|  | Japan | 0.871 |
|  | Andorra | 0.870 |
|  | United States of America | 0.863 |
|  | Finland | 0.860 |
|  | United Kingdom | 0.858 |
|  | Lithuania | 0.858 |
|  | Singapore | 0.856 |
|  | Austria | 0.855 |
|  | Belgium | 0.854 |
|  | New Zealand | 0.850 |
|  | United Arab Emirates | 0.850 |
|  | Kuwait | 0.847 |
|  | Qatar | 0.847 |
|  | Estonia | 0.846 |
|  | Australia | 0.844 |
|  | Slovenia | 0.843 |
|  | France | 0.838 |
|  | Cyprus | 0.836 |
|  | Greenland | 0.836 |
|  | Latvia | 0.831 |
|  | Czechia | 0.829 |
|  | Puerto Rico | 0.825 |
|  | United States Virgin Islands | 0.823 |
|  | Bermuda | 0.821 |
|  | Saudi Arabia | 0.815 |
|  | Poland | 0.812 |
| High-middle SDl region |  |  |
|  | Brunei Darussalam | 0.810 |
|  | Russian Federation | 0.809 |
|  | Israel | 0.809 |
|  | Slovakia | 0.808 |
|  | Italy | 0.806 |
|  | Bahamas | 0.805 |
|  | Guam | 0.802 |
|  | Malta | 0.802 |
|  | Croatia | 0.799 |
|  | Montenegro | 0.797 |
|  | Serbia | 0.792 |
|  | Greece | 0.792 |
|  | Hungary | 0.791 |
|  | Belarus | 0.784 |
|  | Cook Islands | 0.778 |
|  | Northern Mariana Islands | 0.778 |
|  | Oman | 0.774 |
|  | Chile | 0.770 |
|  | Spain | 0.769 |
|  | Trinidad and Tobago | 0.769 |
|  | Romania | 0.766 |
|  | Bulgaria | 0.765 |
|  | Ukraine | 0.761 |
|  | Saint Kitts and Nevis | 0.756 |
|  | Palau | 0.755 |
|  | Bahrain | 0.752 |
|  | North Macedonia | 0.751 |
|  | Antigua and Barbuda | 0.750 |
|  | Dominica | 0.747 |
|  | Barbados | 0.747 |
|  | Portugal | 0.745 |
|  | Malaysia | 0.743 |
|  | Lebanon | 0.741 |
|  | Libya | 0.735 |
|  | Argentina | 0.734 |
|  | Georgia | 0.733 |
|  | Republic of Moldova | 0.732 |
|  | Seychelles | 0.728 |
|  | American Samoa | 0.726 |
|  | Niue | 0.726 |
|  | Jordan | 0.725 |
|  | Bosnia and Herzegovina | 0.723 |
|  | Uruguay | 0.722 |
|  | China | 0.719 |
|  | Kazakhstan | 0.718 |
|  | Mauritius | 0.718 |
|  | Turkey | 0.713 |
| Middle SDI region |  |  |
|  | Albania | 0.707 |
|  | Panama | 0.707 |
|  | Costa Rica | 0.704 |
|  | Armenia | 0.702 |
|  | Sri Lanka | 0.701 |
|  | Iran (Islamic Republic of) | 0.697 |
|  | Azerbaijan | 0.695 |
|  | Tokelau | 0.687 |
|  | Jamaica | 0.683 |
|  | Turkmenistan | 0.683 |
|  | Thailand | 0.683 |
|  | Tunisia | 0.682 |
|  | South Africa | 0.681 |
|  | Saint Lucia | 0.673 |
|  | Grenada | 0.669 |
|  | Cuba | 0.669 |
|  | Fiji | 0.669 |
|  | Ecuador | 0.666 |
|  | Mexico | 0.665 |
|  | Uzbekistan | 0.665 |
|  | Equatorial Guinea | 0.664 |
|  | Iraq | 0.663 |
|  | Peru | 0.662 |
|  | Algeria | 0.660 |
|  | Indonesia | 0.658 |
|  | Maldives | 0.658 |
|  | Colombia | 0.657 |
|  | Philippines | 0.652 |
|  | Guyana | 0.651 |
|  | Paraguay | 0.650 |
|  | Brazil | 0.649 |
|  | Botswana | 0.643 |
|  | Suriname | 0.641 |
|  | Saint Vincent and the Grenadines | 0.641 |
|  | Gabon | 0.639 |
|  | Palestine | 0.629 |
|  | Tonga | 0.629 |
|  | Nauru | 0.628 |
|  | Syrian Arab Republic | 0.623 |
|  | Viet Nam | 0.622 |
|  | Dominican Republic | 0.619 |
| Low-middle SDI region |  |  |
|  | Mongolia | 0.619 |
|  | Namibia | 0.618 |
|  | Belize | 0.611 |
|  | Kyrgyzstan | 0.609 |
|  | Bolivia (Plurinational State of) | 0.604 |
|  | Egypt | 0.604 |
|  | Venezuela (Bolivarian Republic of) | 0.597 |
|  | Samoa | 0.592 |
|  | Micronesia (Federated States of) | 0.588 |
|  | Congo | 0.587 |
|  | Eswatini | 0.586 |
|  | Tuvalu | 0.579 |
|  | India | 0.578 |
|  | Marshall Islands | 0.574 |
|  | Democratic People's Republic of Korea | 0.569 |
|  | El Salvador | 0.566 |
|  | Ghana | 0.563 |
|  | Morocco | 0.562 |
|  | Sudan | 0.543 |
|  | Guatemala | 0.540 |
|  | Tajikistan | 0.537 |
|  | Cabo Verde | 0.534 |
|  | Myanmar | 0.528 |
|  | Kiribati | 0.526 |
|  | Kenya | 0.525 |
|  | Nicaragua | 0.524 |
|  | Honduras | 0.514 |
|  | Lesotho | 0.512 |
|  | Zambia | 0.510 |
|  | Pakistan | 0.504 |
|  | Nigeria | 0.504 |
|  | Sao Tome and Principe | 0.503 |
|  | Mauritania | 0.495 |
|  | Bangladesh | 0.493 |
|  | Lao People's Democratic Republic | 0.489 |
|  | Djibouti | 0.489 |
|  | Angola | 0.483 |
|  | Cameroon | 0.480 |
|  | Comoros | 0.477 |
|  | Bhutan | 0.477 |
|  | Zimbabwe | 0.476 |
|  | Cambodia | 0.474 |
|  | Vanuatu | 0.473 |
| Low SDI region |  |  |
|  | Yemen | 0.454 |
|  | Timor-Leste | 0.451 |
|  | Haiti | 0.449 |
|  | United Republic of Tanzania | 0.449 |
|  | Rwanda | 0.436 |
|  | Nepal | 0.434 |
|  | Solomon Islands | 0.430 |
|  | Uganda | 0.427 |
|  | Côte d'Ivoire | 0.425 |
|  | Papua New Guinea | 0.418 |
|  | Gambia | 0.410 |
|  | Togo | 0.410 |
|  | Senegal | 0.409 |
|  | Eritrea | 0.405 |
|  | Madagascar | 0.401 |
|  | Democratic Republic of the Congo | 0.390 |
|  | Malawi | 0.382 |
|  | Benin | 0.375 |
|  | Ethiopia | 0.361 |
|  | Sierra Leone | 0.359 |
|  | Guinea-Bissau | 0.353 |
|  | Liberia | 0.353 |
|  | Guinea | 0.337 |
|  | Afghanistan | 0.335 |
|  | Mozambique | 0.327 |
|  | Central African Republic | 0.311 |
|  | Burundi | 0.291 |
|  | Burkina Faso | 0.284 |
|  | South Sudan | 0.278 |
|  | Mali | 0.271 |
|  | Chad | 0.244 |
|  | Niger | 0.170 |
|  | Somalia | 0.077 |

**Age-period-cohort analysis**

The age-period-cohort model (APC model) is a process that analyzes the degree of influence of age, period, and cohort on disease morbidity and mortality, controlling for interactions[4]. It can be expressed as follows:

$$Ln(R_{ijk})=\mu+\alpha_{i}+\beta_{j}+\gamma_{k}+\varepsilon$$

where denotes the ASR of CRC in the ith age group at the jth period for the kth birth cohort, $\alpha_{i}$ refers to age effects, $\beta_{j}$ represents period effects, $\gamma_{k}$ is cohort effects, $\varepsilon$ refers to the random error[5].

The NCI's APC Web tool (https://analysistools.nci.nih.gov/apc/) was used to fit the APC models[6]. Typically, age effects, period effects, cohort effects, and random variations account for the temporal pattern of illnesses. In this instance, characterizing the APC results of illness incidence under conditions of GBTC rate is crucial when discussing epidemiology. The term "period effect" in the APC model describes alterations in human factors that impact the incidence rate of GBTC in the general population. These factors include advancements in technology for disease diagnosis, screening and early detection, modifications to disease definition and registration, and enhancements to treatment. Period impacts could result from these artificial causes influencing the rate of illness occurrence at different times. One of the most important factors influencing the development of disease is age. The term "cohort effect" describes the variations in disease rates among generations brought about by exposure to varying degrees of risk factors. Using SDI quintiles, the APC model calculates the APC effect. The fitted time trend displays the period effect, period risk ratio, and period deviation; the age effect represents the age-related prevalence of GBTC using longitudinal and cross-sectional age curves; Cohort RR, cohort bias, and local drift are used to display cohort effects. Within the APC model, the trend within a particular time period or age group is represented by the local drift, whereas the net drift shows the overall trend in prevalence or incidence rate over time. The age effect quantifies the shift in the incidence or prevalence rate associated with aging. A change that impacts people of all ages at a certain point in time is referred to as a periodic effect. The change in the incidence or prevalence of people born in the same year is reflected in the cohort effect. The recognizability constraint that requires the total of the APC coefficients to equal zero solves the covariance problem between age, period, and cohort in the model. However, estimating specific APC effects could be challenging due to this restriction. An estimable function approach, which combines linear constraints of APC coefficients to estimate these impacts, was utilized to tackle this problem. In particular, each effect's net drift (long-term trend) and local drift (deviation from long-term trend) are estimated using the sum to zero constraint. Local drift is the variation from the general change in outcomes over time, whereas net drift is the overall change in results over time. As a result, APC models can provide light on the particular effects of APC on the incidence and prevalence of GBTC across time.

**Decomposition analysis**

We employ Das Gupta's decomposition method to decompose the incidence, death, and DALY rates of GBTC based on aging, population growth, and epidemiological changes[7]. In 2020, Cheng and his colleagues focused on the sensitivity of decomposition analysis methods in determining the decomposition order of three factors: population growth, population aging, and particular age mortality rate, as well as the selection of reference populations[8, 9]. We thoroughly examined the above two techniques and achieved consistent findings since they share the same basic decomposition, which can be briefly expressed as follows (take DALYs for example).

The number of DALYs at each location was obtained from the following formula:

DALY _ay, py, ey_ = $\sum_{i=1}^{17} ($a _i, y_ * p _y_ * e _i, y_)

Where DALY _ay, py, ey_ represented DALYs based on the factors of age structure, population, and DALYs rate for specific year y; a _i_ _y_ represents the proportion of population for the age category i of the 17 age categories in given year y; p _y_ represents the total population in given year y; and e _i, y_ represents DALYs rate given age category i in year y. The contribution of each factor to the change in DALYs from 1990 to 2021 was defined by the effect of one factor changing while the other factors were held constant. For example, the effect of age structure was calculated as:

[(DALY _a2021, p1990, e1990_ + DALY _a2021, p2021, e2021_)/3+ (DALY _a2021, p1990, e2021_ + DALY _a2021, p2021, e1990_)/6] - [(DALY _a1990, p2021, e2021_ + DALY _a1990, p1990, e1990_)/3+ (DALY _a1990, p2021b, e1990_ + DALY _a1990, p1990, e2021_)/6]

**Cross-country inequality analysis**

Concentration index.

The concentration index is a relative measure of inequality that displays the health gradient of multiple subgroups with natural ranking, most commonly education or wealth[10]. It indicates to what extent health indicators are concentrated among vulnerable or advantaged groups. Given that population is ranked based on socioeconomic status, when health indicators are concentrated among vulnerable groups such as the poor or those with lower education levels, the concentration index is negative; When health indicators are concentrated in advantageous groups such as the wealthy or those with higher education, they have a positive value. When there is no inequality, the concentration index is 0. If an individual (the smallest possible subgroup of the population) accounts for 100% of the population's health indicators (theoretically the highest possible relative inequality), this will result in the concentration index approaching its maximum absolute value of -1 or+1. Although ± 1 is the theoretical maximum value of the concentration index, in practice, the absolute value of the concentration index rarely exceeds 0.5, and values between 0.2 and 0.3 are considered to represent a relatively high level of relative inequality. The intuitive method for explaining concentration index is to use a related concept called concentration curve. Like the slope index of inequality, it first ranks a weighted sample of the entire population based on education or wealth (x-axis), from the most vulnerable subgroup (ranked 0) to the most advantageous subgroup (ranked 1). The y-axis represents the cumulative score of health indicators corresponding to each subgroup. The concentration curve is drawn by connecting these points. If the health indicators are concentrated in the advantageous population, the concentration curve is located below the 45 ° diagonal from the lower left corner to the upper right corner, which is the so-called equality line; If health indicators are concentrated among vulnerable groups, the concentration curve is above the equality line. When there is no inequality, the concentration curve is located on the equation line. The concentration index is calculated as twice the area between the assumed contour line and the concentration curve.

Slope index of inequality

The slope index of inequality is used to display the health gradient of multiple subgroups with natural ranking, most commonly education or wealth[10]. The inequality slope index represents the absolute difference in health indicator predictions between individuals with the highest education or wealth levels and those with the lowest education or wealth levels, while using appropriate regression models to consider the overall distribution of education or wealth. To calculate the slope index of inequality, a weighted sample of the entire population is ranked from the most vulnerable subgroup (ranked zero or 0) to the most advantageous subgroup (ranked 1) based on factors such as education or wealth. This ranking is weighted, taking into account the proportional distribution of the population within each subgroup. Then, consider the population of each wealth or education category based on the range of cumulative population distribution and the midpoint of that range. Then, use an appropriate model to regress the health indicators of interest to the median values of wealth or education subgroups, and calculate the predicted values of the two extremes (rank 1 and rank 0) of health indicators. The difference between the predicted values of rank 1 and rank 0 (covering the entire distribution) generates the slope exponent of the inequality value. Therefore, the slope index of inequality represents the difference between the lowest and highest, while considering all other subgroups in the regression (i.e. the impact of education or wealth on the overall population distribution). When the slope of the regression line is flat, the slope exponent of the inequality is 0. When ranking from the weakest to the most favorable, positive values indicate that the health indicator of interest is more common in the most favorable subgroup, while negative values indicate that the indicator is more common in the weakest subgroup. The slope exponent of inequality values has a direct meaning and has the same measurement unit as health indicators, making it very useful. Non-technical audiences can understand the slope index as an estimate of the difference between the worst and best individuals in a population on a given health metric, although they may initially know little about how this number is calculated or why it is more advantageous than simple differences.

**Joinpoint model**

The joinpoint regression model's basic idea is to divide a long-term trend line into multiple segments by model-fitting; each segment is defined by continuous linearity, and the intersections of distinct trend segments are known as turning points. Lastly, we tested significance using the Monte Carlo permutation approach[8]. The joinpoint regression model can be expressed as a segmentation function:

$${E[y|x]=e}^{\beta_{0}+\beta_{1}x+\delta_{1}(x-\tau_{1})^{+}+...+\delta_{k}(x-\tau_{k})^{+}}$$

Where $y$ is mortality rate. $x$ indicates year. $\beta_{0}$ is the constant parameter. $\beta_{1}$ is regression coefficient, $k$ is the number of joinpoints. $\delta_{k}$ denotes the regression coefficient of the k^th^ segment function.

The equation for the estimation of each segment’s APC and the AAPC for each segment can be expressed as:

$$ln(APC)=[\frac{y_{x+1}-y_{x}}{y_{x}}]*100\%=\left( e^{\beta_{1}}-1 \right)*100\%$$

$$ln(AAPC)=(e^{{\sum\omega_{i}\beta_{i}}/{\sum\omega_{i}}}-1)*100\%$$

**Data rating quality**

For GBD 2016, a simple star-rating system from 0 to 5 was developed to give a picture of the quality of data available in a given country over the full time series used in GBD estimates. Countries improve in the star rating as they increase availability, completeness, and detail of their mortality data and reduce the percentage of deaths coded to ill-defined garbage codes or highly aggregated causes[11].

Two dimensions determine this star rating: (I) the percentage of total deaths determined to be major garbage (such as ill-defined). Causes such as “injuries” or “cancer” will also be included in major garbage percentage because this percentage includes use of highly aggregated causes; and (II) the level of completeness of death registration (percentage of total deaths captured by the death registration). These two values were used to create a “percentage well-certified” value between 0 and 1, determined a*s:*

*𝑝𝑐𝑡_w𝑒𝑙𝑙𝑐𝑒𝑟𝑡𝑖𝑓𝑖𝑒𝑑_ = Completeness × (1 – 𝑝𝑐𝑡_𝑚ajgarbage_ )*

The mapping of percentage well certified to star rating is as followed:

0 star*: 0% = 𝑝𝑐𝑡_w𝑒𝑙𝑙𝑐𝑒𝑟𝑡𝑖𝑓𝑖𝑒𝑑_*

1 star: *0% < 𝑝𝑐𝑡_w𝑒𝑙𝑙𝑐𝑒𝑟𝑡𝑖𝑓𝑖𝑒𝑑_* *< 10%*

2 star: *10% <= 𝑝𝑐𝑡_w𝑒𝑙𝑙𝑐𝑒𝑟𝑡𝑖𝑓𝑖𝑒𝑑_* *< 35%*

3 star: *35% <= 𝑝𝑐𝑡_w𝑒𝑙𝑙𝑐𝑒𝑟𝑡𝑖𝑓𝑖𝑒𝑑_ < 65%*

4 star: *65% <= 𝑝𝑐𝑡_w𝑒𝑙𝑙𝑐𝑒𝑟𝑡𝑖𝑓𝑖𝑒𝑑_* *< 85%*

5 star: *𝑝𝑐𝑡_w𝑒𝑙𝑙𝑐𝑒𝑟𝑡𝑖𝑓𝑖𝑒𝑑_*  ≥ 85%

While stars are calculated for each five-year time interval as well as the full time series from 1980 to 2020, stars in the main text are presented for the full time series only.

The data rating quality in 204 countries as follows:

| Location | Time Window | Stars |
| --- | --- | --- |
| Afghanistan | 1980-1984 | 0 |
| Afghanistan | 1985-1989 | 0 |
| Afghanistan | 1990-1994 | 0 |
| Afghanistan | 1995-1999 | 0 |
| Afghanistan | 2000-2004 | 1 |
| Afghanistan | 2005-2009 | 2 |
| Afghanistan | 2010-2020 | 2 |
| Albania | 1980-1984 | 0 |
| Albania | 1985-1989 | 4 |
| Albania | 1990-1994 | 3 |
| Albania | 1995-1999 | 4 |
| Albania | 2000-2004 | 4 |
| Albania | 2005-2009 | 4 |
| Albania | 2010-2020 | 3 |
| Algeria | 1980-1984 | 0 |
| Algeria | 1985-1989 | 0 |
| Algeria | 1990-1994 | 0 |
| Algeria | 1995-1999 | 0 |
| Algeria | 2000-2004 | 0 |
| Algeria | 2005-2009 | 2 |
| Algeria | 2010-2020 | 0 |
| American Samoa | 1980-1984 | 0 |
| American Samoa | 1985-1989 | 0 |
| American Samoa | 1990-1994 | 0 |
| American Samoa | 1995-1999 | 3 |
| American Samoa | 2000-2004 | 4 |
| American Samoa | 2005-2009 | 4 |
| American Samoa | 2010-2020 | 4 |
| Andorra | 1980-1984 | 0 |
| Andorra | 1985-1989 | 0 |
| Andorra | 1990-1994 | 0 |
| Andorra | 1995-1999 | 0 |
| Andorra | 2000-2004 | 0 |
| Andorra | 2005-2009 | 0 |
| Andorra | 2010-2020 | 3 |
| Angola | 1980-1984 | 0 |
| Angola | 1985-1989 | 0 |
| Angola | 1990-1994 | 0 |
| Angola | 1995-1999 | 0 |
| Angola | 2000-2004 | 0 |
| Angola | 2005-2009 | 0 |
| Angola | 2010-2020 | 1 |
| Antigua and Barbuda | 1980-1984 | 3 |
| Antigua and Barbuda | 1985-1989 | 4 |
| Antigua and Barbuda | 1990-1994 | 4 |
| Antigua and Barbuda | 1995-1999 | 4 |
| Antigua and Barbuda | 2000-2004 | 4 |
| Antigua and Barbuda | 2005-2009 | 4 |
| Antigua and Barbuda | 2010-2020 | 4 |
| Argentina | 1980-1984 | 4 |
| Argentina | 1985-1989 | 4 |
| Argentina | 1990-1994 | 4 |
| Argentina | 1995-1999 | 4 |
| Argentina | 2000-2004 | 4 |
| Argentina | 2005-2009 | 4 |
| Argentina | 2010-2020 | 4 |
| Armenia | 1980-1984 | 4 |
| Armenia | 1985-1989 | 4 |
| Armenia | 1990-1994 | 4 |
| Armenia | 1995-1999 | 4 |
| Armenia | 2000-2004 | 5 |
| Armenia | 2005-2009 | 5 |
| Armenia | 2010-2020 | 5 |
| Australia | 1980-1984 | 5 |
| Australia | 1985-1989 | 5 |
| Australia | 1990-1994 | 5 |
| Australia | 1995-1999 | 5 |
| Australia | 2000-2004 | 5 |
| Australia | 2005-2009 | 5 |
| Australia | 2010-2020 | 5 |
| Austria | 1980-1984 | 5 |
| Austria | 1985-1989 | 5 |
| Austria | 1990-1994 | 5 |
| Austria | 1995-1999 | 5 |
| Austria | 2000-2004 | 5 |
| Austria | 2005-2009 | 5 |
| Austria | 2010-2020 | 5 |
| Azerbaijan | 1980-1984 | 4 |
| Azerbaijan | 1985-1989 | 4 |
| Azerbaijan | 1990-1994 | 4 |
| Azerbaijan | 1995-1999 | 4 |
| Azerbaijan | 2000-2004 | 4 |
| Azerbaijan | 2005-2009 | 3 |
| Azerbaijan | 2010-2020 | 0 |
| Bahrain | 1980-1984 | 0 |
| Bahrain | 1985-1989 | 4 |
| Bahrain | 1990-1994 | 0 |
| Bahrain | 1995-1999 | 3 |
| Bahrain | 2000-2004 | 3 |
| Bahrain | 2005-2009 | 3 |
| Bahrain | 2010-2020 | 3 |
| Bangladesh | 1980-1984 | 1 |
| Bangladesh | 1985-1989 | 1 |
| Bangladesh | 1990-1994 | 2 |
| Bangladesh | 1995-1999 | 1 |
| Bangladesh | 2000-2004 | 2 |
| Bangladesh | 2005-2009 | 2 |
| Bangladesh | 2010-2020 | 3 |
| Barbados | 1980-1984 | 4 |
| Barbados | 1985-1989 | 4 |
| Barbados | 1990-1994 | 4 |
| Barbados | 1995-1999 | 4 |
| Barbados | 2000-2004 | 4 |
| Barbados | 2005-2009 | 4 |
| Barbados | 2010-2020 | 4 |
| Belarus | 1980-1984 | 5 |
| Belarus | 1985-1989 | 5 |
| Belarus | 1990-1994 | 4 |
| Belarus | 1995-1999 | 4 |
| Belarus | 2000-2004 | 4 |
| Belarus | 2005-2009 | 4 |
| Belarus | 2010-2020 | 5 |
| Belgium | 1980-1984 | 4 |
| Belgium | 1985-1989 | 4 |
| Belgium | 1990-1994 | 4 |
| Belgium | 1995-1999 | 5 |
| Belgium | 2000-2004 | 5 |
| Belgium | 2005-2009 | 5 |
| Belgium | 2010-2020 | 4 |
| Belize | 1980-1984 | 3 |
| Belize | 1985-1989 | 3 |
| Belize | 1990-1994 | 3 |
| Belize | 1995-1999 | 4 |
| Belize | 2000-2004 | 4 |
| Belize | 2005-2009 | 5 |
| Belize | 2010-2020 | 5 |
| Benin | 1980-1984 | 0 |
| Benin | 1985-1989 | 1 |
| Benin | 1990-1994 | 0 |
| Benin | 1995-1999 | 0 |
| Benin | 2000-2004 | 0 |
| Benin | 2005-2009 | 0 |
| Benin | 2010-2020 | 1 |
| Bermuda | 1980-1984 | 5 |
| Bermuda | 1985-1989 | 5 |
| Bermuda | 1990-1994 | 5 |
| Bermuda | 1995-1999 | 5 |
| Bermuda | 2000-2004 | 5 |
| Bermuda | 2005-2009 | 5 |
| Bermuda | 2010-2020 | 5 |
| Bhutan | 1980-1984 | 0 |
| Bhutan | 1985-1989 | 0 |
| Bhutan | 1990-1994 | 0 |
| Bhutan | 1995-1999 | 0 |
| Bhutan | 2000-2004 | 0 |
| Bhutan | 2005-2009 | 0 |
| Bhutan | 2010-2020 | 0 |
| Bolivia | 1980-1984 | 0 |
| Bolivia | 1985-1989 | 0 |
| Bolivia | 1990-1994 | 0 |
| Bolivia | 1995-1999 | 0 |
| Bolivia | 2000-2004 | 2 |
| Bolivia | 2005-2009 | 0 |
| Bolivia | 2010-2020 | 0 |
| Bosnia and Herzegovina | 1980-1984 | 0 |
| Bosnia and Herzegovina | 1985-1989 | 3 |
| Bosnia and Herzegovina | 1990-1994 | 4 |
| Bosnia and Herzegovina | 1995-1999 | 0 |
| Bosnia and Herzegovina | 2000-2004 | 0 |
| Bosnia and Herzegovina | 2005-2009 | 0 |
| Bosnia and Herzegovina | 2010-2020 | 4 |
| Botswana | 1980-1984 | 0 |
| Botswana | 1985-1989 | 0 |
| Botswana | 1990-1994 | 0 |
| Botswana | 1995-1999 | 0 |
| Botswana | 2000-2004 | 0 |
| Botswana | 2005-2009 | 0 |
| Botswana | 2010-2020 | 0 |
| Brazil | 1980-1984 | 3 |
| Brazil | 1985-1989 | 3 |
| Brazil | 1990-1994 | 3 |
| Brazil | 1995-1999 | 4 |
| Brazil | 2000-2004 | 4 |
| Brazil | 2005-2009 | 4 |
| Brazil | 2010-2020 | 4 |
| Brunei | 1980-1984 | 0 |
| Brunei | 1985-1989 | 0 |
| Brunei | 1990-1994 | 0 |
| Brunei | 1995-1999 | 3 |
| Brunei | 2000-2004 | 4 |
| Brunei | 2005-2009 | 4 |
| Brunei | 2010-2020 | 4 |
| Bulgaria | 1980-1984 | 4 |
| Bulgaria | 1985-1989 | 4 |
| Bulgaria | 1990-1994 | 4 |
| Bulgaria | 1995-1999 | 4 |
| Bulgaria | 2000-2004 | 4 |
| Bulgaria | 2005-2009 | 4 |
| Bulgaria | 2010-2020 | 4 |
| Burkina Faso | 1980-1984 | 1 |
| Burkina Faso | 1985-1989 | 0 |
| Burkina Faso | 1990-1994 | 0 |
| Burkina Faso | 1995-1999 | 1 |
| Burkina Faso | 2000-2004 | 1 |
| Burkina Faso | 2005-2009 | 1 |
| Burkina Faso | 2010-2020 | 1 |
| Burundi | 1980-1984 | 0 |
| Burundi | 1985-1989 | 0 |
| Burundi | 1990-1994 | 1 |
| Burundi | 1995-1999 | 0 |
| Burundi | 2000-2004 | 0 |
| Burundi | 2005-2009 | 0 |
| Burundi | 2010-2020 | 0 |
| Cambodia | 1980-1984 | 0 |
| Cambodia | 1985-1989 | 0 |
| Cambodia | 1990-1994 | 0 |
| Cambodia | 1995-1999 | 0 |
| Cambodia | 2000-2004 | 1 |
| Cambodia | 2005-2009 | 1 |
| Cambodia | 2010-2020 | 0 |
| Cameroon | 1980-1984 | 0 |
| Cameroon | 1985-1989 | 0 |
| Cameroon | 1990-1994 | 0 |
| Cameroon | 1995-1999 | 0 |
| Cameroon | 2000-2004 | 0 |
| Cameroon | 2005-2009 | 0 |
| Cameroon | 2010-2020 | 0 |
| Canada | 1980-1984 | 5 |
| Canada | 1985-1989 | 5 |
| Canada | 1990-1994 | 5 |
| Canada | 1995-1999 | 5 |
| Canada | 2000-2004 | 5 |
| Canada | 2005-2009 | 5 |
| Canada | 2010-2020 | 5 |
| Cape Verde | 1980-1984 | 3 |
| Cape Verde | 1985-1989 | 0 |
| Cape Verde | 1990-1994 | 1 |
| Cape Verde | 1995-1999 | 0 |
| Cape Verde | 2000-2004 | 0 |
| Cape Verde | 2005-2009 | 0 |
| Cape Verde | 2010-2020 | 4 |
| Central African Republic | 1980-1984 | 0 |
| Central African Republic | 1985-1989 | 0 |
| Central African Republic | 1990-1994 | 0 |
| Central African Republic | 1995-1999 | 0 |
| Central African Republic | 2000-2004 | 0 |
| Central African Republic | 2005-2009 | 0 |
| Central African Republic | 2010-2020 | 0 |
| Chad | 1980-1984 | 0 |
| Chad | 1985-1989 | 0 |
| Chad | 1990-1994 | 0 |
| Chad | 1995-1999 | 0 |
| Chad | 2000-2004 | 0 |
| Chad | 2005-2009 | 0 |
| Chad | 2010-2020 | 1 |
| Chile | 1980-1984 | 4 |
| Chile | 1985-1989 | 4 |
| Chile | 1990-1994 | 4 |
| Chile | 1995-1999 | 5 |
| Chile | 2000-2004 | 5 |
| Chile | 2005-2009 | 5 |
| Chile | 2010-2020 | 5 |
| China | 1980-1984 | 0 |
| China | 1985-1989 | 1 |
| China | 1990-1994 | 4 |
| China | 1995-1999 | 4 |
| China | 2000-2004 | 4 |
| China | 2005-2009 | 4 |
| China | 2010-2020 | 4 |
| Colombia | 1980-1984 | 4 |
| Colombia | 1985-1989 | 4 |
| Colombia | 1990-1994 | 4 |
| Colombia | 1995-1999 | 5 |
| Colombia | 2000-2004 | 5 |
| Colombia | 2005-2009 | 5 |
| Colombia | 2010-2020 | 5 |
| Comoros | 1980-1984 | 0 |
| Comoros | 1985-1989 | 0 |
| Comoros | 1990-1994 | 0 |
| Comoros | 1995-1999 | 0 |
| Comoros | 2000-2004 | 0 |
| Comoros | 2005-2009 | 0 |
| Comoros | 2010-2020 | 0 |
| Congo (Brazzaville) | 1980-1984 | 0 |
| Congo (Brazzaville) | 1985-1989 | 0 |
| Congo (Brazzaville) | 1990-1994 | 0 |
| Congo (Brazzaville) | 1995-1999 | 0 |
| Congo (Brazzaville) | 2000-2004 | 0 |
| Congo (Brazzaville) | 2005-2009 | 0 |
| Congo (Brazzaville) | 2010-2020 | 0 |
| Cook Islands | 1980-1984 | 0 |
| Cook Islands | 1985-1989 | 0 |
| Cook Islands | 1990-1994 | 0 |
| Cook Islands | 1995-1999 | 0 |
| Cook Islands | 2000-2004 | 3 |
| Cook Islands | 2005-2009 | 3 |
| Cook Islands | 2010-2020 | 4 |
| Costa Rica | 1980-1984 | 4 |
| Costa Rica | 1985-1989 | 4 |
| Costa Rica | 1990-1994 | 4 |
| Costa Rica | 1995-1999 | 5 |
| Costa Rica | 2000-2004 | 5 |
| Costa Rica | 2005-2009 | 5 |
| Costa Rica | 2010-2020 | 5 |
| Croatia | 1980-1984 | 0 |
| Croatia | 1985-1989 | 4 |
| Croatia | 1990-1994 | 5 |
| Croatia | 1995-1999 | 4 |
| Croatia | 2000-2004 | 5 |
| Croatia | 2005-2009 | 5 |
| Croatia | 2010-2020 | 5 |
| Cuba | 1980-1984 | 5 |
| Cuba | 1985-1989 | 5 |
| Cuba | 1990-1994 | 5 |
| Cuba | 1995-1999 | 5 |
| Cuba | 2000-2004 | 5 |
| Cuba | 2005-2009 | 5 |
| Cuba | 2010-2020 | 5 |
| Cyprus | 1980-1984 | 0 |
| Cyprus | 1985-1989 | 0 |
| Cyprus | 1990-1994 | 0 |
| Cyprus | 1995-1999 | 2 |
| Cyprus | 2000-2004 | 3 |
| Cyprus | 2005-2009 | 3 |
| Cyprus | 2010-2020 | 4 |
| Czechia | 1980-1984 | 0 |
| Czechia | 1985-1989 | 5 |
| Czechia | 1990-1994 | 5 |
| Czechia | 1995-1999 | 5 |
| Czechia | 2000-2004 | 5 |
| Czechia | 2005-2009 | 5 |
| Czechia | 2010-2020 | 5 |
| Côte d'Ivoire | 1980-1984 | 0 |
| Côte d'Ivoire | 1985-1989 | 1 |
| Côte d'Ivoire | 1990-1994 | 1 |
| Côte d'Ivoire | 1995-1999 | 0 |
| Côte d'Ivoire | 2000-2004 | 0 |
| Côte d'Ivoire | 2005-2009 | 1 |
| Côte d'Ivoire | 2010-2020 | 1 |
| DR Congo | 1980-1984 | 0 |
| DR Congo | 1985-1989 | 1 |
| DR Congo | 1990-1994 | 1 |
| DR Congo | 1995-1999 | 0 |
| DR Congo | 2000-2004 | 0 |
| DR Congo | 2005-2009 | 0 |
| DR Congo | 2010-2020 | 1 |
| Denmark | 1980-1984 | 4 |
| Denmark | 1985-1989 | 4 |
| Denmark | 1990-1994 | 5 |
| Denmark | 1995-1999 | 5 |
| Denmark | 2000-2004 | 5 |
| Denmark | 2005-2009 | 5 |
| Denmark | 2010-2020 | 5 |
| Djibouti | 1980-1984 | 0 |
| Djibouti | 1985-1989 | 0 |
| Djibouti | 1990-1994 | 0 |
| Djibouti | 1995-1999 | 0 |
| Djibouti | 2000-2004 | 0 |
| Djibouti | 2005-2009 | 0 |
| Djibouti | 2010-2020 | 0 |
| Dominica | 1980-1984 | 3 |
| Dominica | 1985-1989 | 3 |
| Dominica | 1990-1994 | 3 |
| Dominica | 1995-1999 | 3 |
| Dominica | 2000-2004 | 3 |
| Dominica | 2005-2009 | 4 |
| Dominica | 2010-2020 | 4 |
| Dominican Republic | 1980-1984 | 3 |
| Dominican Republic | 1985-1989 | 3 |
| Dominican Republic | 1990-1994 | 3 |
| Dominican Republic | 1995-1999 | 3 |
| Dominican Republic | 2000-2004 | 3 |
| Dominican Republic | 2005-2009 | 3 |
| Dominican Republic | 2010-2020 | 3 |
| Ecuador | 1980-1984 | 4 |
| Ecuador | 1985-1989 | 4 |
| Ecuador | 1990-1994 | 4 |
| Ecuador | 1995-1999 | 4 |
| Ecuador | 2000-2004 | 3 |
| Ecuador | 2005-2009 | 3 |
| Ecuador | 2010-2020 | 5 |
| Egypt | 1980-1984 | 2 |
| Egypt | 1985-1989 | 2 |
| Egypt | 1990-1994 | 3 |
| Egypt | 1995-1999 | 0 |
| Egypt | 2000-2004 | 3 |
| Egypt | 2005-2009 | 3 |
| Egypt | 2010-2020 | 3 |
| El Salvador | 1980-1984 | 3 |
| El Salvador | 1985-1989 | 0 |
| El Salvador | 1990-1994 | 3 |
| El Salvador | 1995-1999 | 3 |
| El Salvador | 2000-2004 | 4 |
| El Salvador | 2005-2009 | 4 |
| El Salvador | 2010-2020 | 3 |
| Equatorial Guinea | 1980-1984 | 0 |
| Equatorial Guinea | 1985-1989 | 0 |
| Equatorial Guinea | 1990-1994 | 0 |
| Equatorial Guinea | 1995-1999 | 0 |
| Equatorial Guinea | 2000-2004 | 0 |
| Equatorial Guinea | 2005-2009 | 0 |
| Equatorial Guinea | 2010-2020 | 0 |
| Eritrea | 1980-1984 | 0 |
| Eritrea | 1985-1989 | 0 |
| Eritrea | 1990-1994 | 0 |
| Eritrea | 1995-1999 | 0 |
| Eritrea | 2000-2004 | 0 |
| Eritrea | 2005-2009 | 0 |
| Eritrea | 2010-2020 | 0 |
| Estonia | 1980-1984 | 5 |
| Estonia | 1985-1989 | 5 |
| Estonia | 1990-1994 | 5 |
| Estonia | 1995-1999 | 5 |
| Estonia | 2000-2004 | 5 |
| Estonia | 2005-2009 | 5 |
| Estonia | 2010-2020 | 5 |
| Eswatini | 1980-1984 | 0 |
| Eswatini | 1985-1989 | 0 |
| Eswatini | 1990-1994 | 0 |
| Eswatini | 1995-1999 | 0 |
| Eswatini | 2000-2004 | 1 |
| Eswatini | 2005-2009 | 0 |
| Eswatini | 2010-2020 | 0 |
| Ethiopia | 1980-1984 | 0 |
| Ethiopia | 1985-1989 | 1 |
| Ethiopia | 1990-1994 | 1 |
| Ethiopia | 1995-1999 | 1 |
| Ethiopia | 2000-2004 | 1 |
| Ethiopia | 2005-2009 | 1 |
| Ethiopia | 2010-2020 | 2 |
| Federated States of Micronesia | 1980-1984 | 0 |
| Federated States of Micronesia | 1985-1989 | 0 |
| Federated States of Micronesia | 1990-1994 | 0 |
| Federated States of Micronesia | 1995-1999 | 0 |
| Federated States of Micronesia | 2000-2004 | 0 |
| Federated States of Micronesia | 2005-2009 | 0 |
| Federated States of Micronesia | 2010-2020 | 0 |
| Fiji | 1980-1984 | 0 |
| Fiji | 1985-1989 | 0 |
| Fiji | 1990-1994 | 0 |
| Fiji | 1995-1999 | 2 |
| Fiji | 2000-2004 | 3 |
| Fiji | 2005-2009 | 3 |
| Fiji | 2010-2020 | 4 |
| Finland | 1980-1984 | 4 |
| Finland | 1985-1989 | 5 |
| Finland | 1990-1994 | 5 |
| Finland | 1995-1999 | 5 |
| Finland | 2000-2004 | 5 |
| Finland | 2005-2009 | 5 |
| Finland | 2010-2020 | 5 |
| France | 1980-1984 | 4 |
| France | 1985-1989 | 4 |
| France | 1990-1994 | 4 |
| France | 1995-1999 | 4 |
| France | 2000-2004 | 4 |
| France | 2005-2009 | 4 |
| France | 2010-2020 | 4 |
| Gabon | 1980-1984 | 0 |
| Gabon | 1985-1989 | 0 |
| Gabon | 1990-1994 | 0 |
| Gabon | 1995-1999 | 0 |
| Gabon | 2000-2004 | 0 |
| Gabon | 2005-2009 | 0 |
| Gabon | 2010-2020 | 0 |
| Georgia | 1980-1984 | 4 |
| Georgia | 1985-1989 | 4 |
| Georgia | 1990-1994 | 4 |
| Georgia | 1995-1999 | 4 |
| Georgia | 2000-2004 | 4 |
| Georgia | 2005-2009 | 3 |
| Georgia | 2010-2020 | 4 |
| Germany | 1980-1984 | 4 |
| Germany | 1985-1989 | 4 |
| Germany | 1990-1994 | 5 |
| Germany | 1995-1999 | 5 |
| Germany | 2000-2004 | 5 |
| Germany | 2005-2009 | 5 |
| Germany | 2010-2020 | 5 |
| Ghana | 1980-1984 | 0 |
| Ghana | 1985-1989 | 1 |
| Ghana | 1990-1994 | 1 |
| Ghana | 1995-1999 | 1 |
| Ghana | 2000-2004 | 1 |
| Ghana | 2005-2009 | 2 |
| Ghana | 2010-2020 | 1 |
| Greece | 1980-1984 | 4 |
| Greece | 1985-1989 | 4 |
| Greece | 1990-1994 | 4 |
| Greece | 1995-1999 | 4 |
| Greece | 2000-2004 | 4 |
| Greece | 2005-2009 | 4 |
| Greece | 2010-2020 | 4 |
| Greenland | 1980-1984 | 0 |
| Greenland | 1985-1989 | 0 |
| Greenland | 1990-1994 | 0 |
| Greenland | 1995-1999 | 4 |
| Greenland | 2000-2004 | 5 |
| Greenland | 2005-2009 | 5 |
| Greenland | 2010-2020 | 4 |
| Grenada | 1980-1984 | 4 |
| Grenada | 1985-1989 | 3 |
| Grenada | 1990-1994 | 4 |
| Grenada | 1995-1999 | 3 |
| Grenada | 2000-2004 | 4 |
| Grenada | 2005-2009 | 4 |
| Grenada | 2010-2020 | 5 |
| Guam | 1980-1984 | 0 |
| Guam | 1985-1989 | 0 |
| Guam | 1990-1994 | 4 |
| Guam | 1995-1999 | 4 |
| Guam | 2000-2004 | 4 |
| Guam | 2005-2009 | 4 |
| Guam | 2010-2020 | 4 |
| Guatemala | 1980-1984 | 4 |
| Guatemala | 1985-1989 | 3 |
| Guatemala | 1990-1994 | 4 |
| Guatemala | 1995-1999 | 4 |
| Guatemala | 2000-2004 | 4 |
| Guatemala | 2005-2009 | 4 |
| Guatemala | 2010-2020 | 4 |
| Guinea | 1980-1984 | 0 |
| Guinea | 1985-1989 | 0 |
| Guinea | 1990-1994 | 0 |
| Guinea | 1995-1999 | 1 |
| Guinea | 2000-2004 | 0 |
| Guinea | 2005-2009 | 0 |
| Guinea | 2010-2020 | 0 |
| Guinea-Bissau | 1980-1984 | 0 |
| Guinea-Bissau | 1985-1989 | 0 |
| Guinea-Bissau | 1990-1994 | 1 |
| Guinea-Bissau | 1995-1999 | 1 |
| Guinea-Bissau | 2000-2004 | 0 |
| Guinea-Bissau | 2005-2009 | 0 |
| Guinea-Bissau | 2010-2020 | 0 |
| Guyana | 1980-1984 | 3 |
| Guyana | 1985-1989 | 3 |
| Guyana | 1990-1994 | 3 |
| Guyana | 1995-1999 | 4 |
| Guyana | 2000-2004 | 4 |
| Guyana | 2005-2009 | 4 |
| Guyana | 2010-2020 | 4 |
| Haiti | 1980-1984 | 2 |
| Haiti | 1985-1989 | 1 |
| Haiti | 1990-1994 | 1 |
| Haiti | 1995-1999 | 2 |
| Haiti | 2000-2004 | 1 |
| Haiti | 2005-2009 | 0 |
| Haiti | 2010-2020 | 0 |
| Honduras | 1980-1984 | 2 |
| Honduras | 1985-1989 | 3 |
| Honduras | 1990-1994 | 3 |
| Honduras | 1995-1999 | 2 |
| Honduras | 2000-2004 | 2 |
| Honduras | 2005-2009 | 2 |
| Honduras | 2010-2020 | 2 |
| Hungary | 1980-1984 | 5 |
| Hungary | 1985-1989 | 5 |
| Hungary | 1990-1994 | 5 |
| Hungary | 1995-1999 | 5 |
| Hungary | 2000-2004 | 5 |
| Hungary | 2005-2009 | 5 |
| Hungary | 2010-2020 | 5 |
| Iceland | 1980-1984 | 5 |
| Iceland | 1985-1989 | 5 |
| Iceland | 1990-1994 | 5 |
| Iceland | 1995-1999 | 5 |
| Iceland | 2000-2004 | 5 |
| Iceland | 2005-2009 | 5 |
| Iceland | 2010-2020 | 5 |
| India | 1980-1984 | 1 |
| India | 1985-1989 | 1 |
| India | 1990-1994 | 1 |
| India | 1995-1999 | 1 |
| India | 2000-2004 | 1 |
| India | 2005-2009 | 3 |
| India | 2010-2020 | 3 |
| Indonesia | 1980-1984 | 1 |
| Indonesia | 1985-1989 | 0 |
| Indonesia | 1990-1994 | 1 |
| Indonesia | 1995-1999 | 1 |
| Indonesia | 2000-2004 | 1 |
| Indonesia | 2005-2009 | 3 |
| Indonesia | 2010-2020 | 3 |
| Iran | 1980-1984 | 0 |
| Iran | 1985-1989 | 0 |
| Iran | 1990-1994 | 0 |
| Iran | 1995-1999 | 0 |
| Iran | 2000-2004 | 3 |
| Iran | 2005-2009 | 3 |
| Iran | 2010-2020 | 4 |
| Iraq | 1980-1984 | 0 |
| Iraq | 1985-1989 | 0 |
| Iraq | 1990-1994 | 0 |
| Iraq | 1995-1999 | 0 |
| Iraq | 2000-2004 | 0 |
| Iraq | 2005-2009 | 3 |
| Iraq | 2010-2020 | 3 |
| Ireland | 1980-1984 | 5 |
| Ireland | 1985-1989 | 5 |
| Ireland | 1990-1994 | 5 |
| Ireland | 1995-1999 | 5 |
| Ireland | 2000-2004 | 5 |
| Ireland | 2005-2009 | 5 |
| Ireland | 2010-2020 | 5 |
| Israel | 1980-1984 | 4 |
| Israel | 1985-1989 | 4 |
| Israel | 1990-1994 | 4 |
| Israel | 1995-1999 | 4 |
| Israel | 2000-2004 | 4 |
| Israel | 2005-2009 | 4 |
| Israel | 2010-2020 | 4 |
| Italy | 1980-1984 | 5 |
| Italy | 1985-1989 | 5 |
| Italy | 1990-1994 | 5 |
| Italy | 1995-1999 | 5 |
| Italy | 2000-2004 | 5 |
| Italy | 2005-2009 | 5 |
| Italy | 2010-2020 | 5 |
| Jamaica | 1980-1984 | 4 |
| Jamaica | 1985-1989 | 4 |
| Jamaica | 1990-1994 | 4 |
| Jamaica | 1995-1999 | 0 |
| Jamaica | 2000-2004 | 4 |
| Jamaica | 2005-2009 | 4 |
| Jamaica | 2010-2020 | 5 |
| Japan | 1980-1984 | 4 |
| Japan | 1985-1989 | 4 |
| Japan | 1990-1994 | 4 |
| Japan | 1995-1999 | 5 |
| Japan | 2000-2004 | 5 |
| Japan | 2005-2009 | 5 |
| Japan | 2010-2020 | 5 |
| Jordan | 1980-1984 | 0 |
| Jordan | 1985-1989 | 0 |
| Jordan | 1990-1994 | 0 |
| Jordan | 1995-1999 | 1 |
| Jordan | 2000-2004 | 4 |
| Jordan | 2005-2009 | 4 |
| Jordan | 2010-2020 | 4 |
| Kazakhstan | 1980-1984 | 4 |
| Kazakhstan | 1985-1989 | 4 |
| Kazakhstan | 1990-1994 | 5 |
| Kazakhstan | 1995-1999 | 5 |
| Kazakhstan | 2000-2004 | 4 |
| Kazakhstan | 2005-2009 | 4 |
| Kazakhstan | 2010-2020 | 4 |
| Kenya | 1980-1984 | 0 |
| Kenya | 1985-1989 | 1 |
| Kenya | 1990-1994 | 0 |
| Kenya | 1995-1999 | 1 |
| Kenya | 2000-2004 | 1 |
| Kenya | 2005-2009 | 1 |
| Kenya | 2010-2020 | 2 |
| Kiribati | 1980-1984 | 0 |
| Kiribati | 1985-1989 | 0 |
| Kiribati | 1990-1994 | 3 |
| Kiribati | 1995-1999 | 3 |
| Kiribati | 2000-2004 | 2 |
| Kiribati | 2005-2009 | 0 |
| Kiribati | 2010-2020 | 0 |
| Kuwait | 1980-1984 | 4 |
| Kuwait | 1985-1989 | 4 |
| Kuwait | 1990-1994 | 4 |
| Kuwait | 1995-1999 | 4 |
| Kuwait | 2000-2004 | 4 |
| Kuwait | 2005-2009 | 4 |
| Kuwait | 2010-2020 | 4 |
| Kyrgyzstan | 1980-1984 | 4 |
| Kyrgyzstan | 1985-1989 | 4 |
| Kyrgyzstan | 1990-1994 | 4 |
| Kyrgyzstan | 1995-1999 | 4 |
| Kyrgyzstan | 2000-2004 | 5 |
| Kyrgyzstan | 2005-2009 | 5 |
| Kyrgyzstan | 2010-2020 | 5 |
| Laos | 1980-1984 | 0 |
| Laos | 1985-1989 | 1 |
| Laos | 1990-1994 | 0 |
| Laos | 1995-1999 | 0 |
| Laos | 2000-2004 | 0 |
| Laos | 2005-2009 | 0 |
| Laos | 2010-2020 | 0 |
| Latvia | 1980-1984 | 5 |
| Latvia | 1985-1989 | 5 |
| Latvia | 1990-1994 | 5 |
| Latvia | 1995-1999 | 5 |
| Latvia | 2000-2004 | 5 |
| Latvia | 2005-2009 | 5 |
| Latvia | 2010-2020 | 5 |
| Lebanon | 1980-1984 | 0 |
| Lebanon | 1985-1989 | 1 |
| Lebanon | 1990-1994 | 0 |
| Lebanon | 1995-1999 | 0 |
| Lebanon | 2000-2004 | 1 |
| Lebanon | 2005-2009 | 0 |
| Lebanon | 2010-2020 | 3 |
| Lesotho | 1980-1984 | 0 |
| Lesotho | 1985-1989 | 0 |
| Lesotho | 1990-1994 | 0 |
| Lesotho | 1995-1999 | 0 |
| Lesotho | 2000-2004 | 0 |
| Lesotho | 2005-2009 | 0 |
| Lesotho | 2010-2020 | 0 |
| Liberia | 1980-1984 | 1 |
| Liberia | 1985-1989 | 1 |
| Liberia | 1990-1994 | 1 |
| Liberia | 1995-1999 | 0 |
| Liberia | 2000-2004 | 0 |
| Liberia | 2005-2009 | 0 |
| Liberia | 2010-2020 | 0 |
| Libya | 1980-1984 | 0 |
| Libya | 1985-1989 | 0 |
| Libya | 1990-1994 | 0 |
| Libya | 1995-1999 | 0 |
| Libya | 2000-2004 | 0 |
| Libya | 2005-2009 | 2 |
| Libya | 2010-2020 | 2 |
| Lithuania | 1980-1984 | 5 |
| Lithuania | 1985-1989 | 5 |
| Lithuania | 1990-1994 | 5 |
| Lithuania | 1995-1999 | 5 |
| Lithuania | 2000-2004 | 5 |
| Lithuania | 2005-2009 | 5 |
| Lithuania | 2010-2020 | 5 |
| Luxembourg | 1980-1984 | 5 |
| Luxembourg | 1985-1989 | 5 |
| Luxembourg | 1990-1994 | 5 |
| Luxembourg | 1995-1999 | 5 |
| Luxembourg | 2000-2004 | 4 |
| Luxembourg | 2005-2009 | 4 |
| Luxembourg | 2010-2020 | 4 |
| Madagascar | 1980-1984 | 1 |
| Madagascar | 1985-1989 | 1 |
| Madagascar | 1990-1994 | 1 |
| Madagascar | 1995-1999 | 1 |
| Madagascar | 2000-2004 | 0 |
| Madagascar | 2005-2009 | 0 |
| Madagascar | 2010-2020 | 0 |
| Malawi | 1980-1984 | 0 |
| Malawi | 1985-1989 | 1 |
| Malawi | 1990-1994 | 0 |
| Malawi | 1995-1999 | 1 |
| Malawi | 2000-2004 | 1 |
| Malawi | 2005-2009 | 1 |
| Malawi | 2010-2020 | 1 |
| Malaysia | 1980-1984 | 3 |
| Malaysia | 1985-1989 | 0 |
| Malaysia | 1990-1994 | 0 |
| Malaysia | 1995-1999 | 2 |
| Malaysia | 2000-2004 | 3 |
| Malaysia | 2005-2009 | 3 |
| Malaysia | 2010-2020 | 3 |
| Maldives | 1980-1984 | 0 |
| Maldives | 1985-1989 | 0 |
| Maldives | 1990-1994 | 0 |
| Maldives | 1995-1999 | 0 |
| Maldives | 2000-2004 | 3 |
| Maldives | 2005-2009 | 3 |
| Maldives | 2010-2020 | 3 |
| Mali | 1980-1984 | 1 |
| Mali | 1985-1989 | 0 |
| Mali | 1990-1994 | 1 |
| Mali | 1995-1999 | 0 |
| Mali | 2000-2004 | 0 |
| Mali | 2005-2009 | 0 |
| Mali | 2010-2020 | 1 |
| Malta | 1980-1984 | 4 |
| Malta | 1985-1989 | 5 |
| Malta | 1990-1994 | 5 |
| Malta | 1995-1999 | 5 |
| Malta | 2000-2004 | 5 |
| Malta | 2005-2009 | 5 |
| Malta | 2010-2020 | 5 |
| Marshall Islands | 1980-1984 | 0 |
| Marshall Islands | 1985-1989 | 0 |
| Marshall Islands | 1990-1994 | 0 |
| Marshall Islands | 1995-1999 | 0 |
| Marshall Islands | 2000-2004 | 0 |
| Marshall Islands | 2005-2009 | 0 |
| Marshall Islands | 2010-2020 | 0 |
| Mauritania | 1980-1984 | 0 |
| Mauritania | 1985-1989 | 0 |
| Mauritania | 1990-1994 | 0 |
| Mauritania | 1995-1999 | 0 |
| Mauritania | 2000-2004 | 0 |
| Mauritania | 2005-2009 | 0 |
| Mauritania | 2010-2020 | 0 |
| Mauritius | 1980-1984 | 4 |
| Mauritius | 1985-1989 | 4 |
| Mauritius | 1990-1994 | 4 |
| Mauritius | 1995-1999 | 4 |
| Mauritius | 2000-2004 | 4 |
| Mauritius | 2005-2009 | 5 |
| Mauritius | 2010-2020 | 5 |
| Mexico | 1980-1984 | 3 |
| Mexico | 1985-1989 | 4 |
| Mexico | 1990-1994 | 4 |
| Mexico | 1995-1999 | 4 |
| Mexico | 2000-2004 | 4 |
| Mexico | 2005-2009 | 4 |
| Mexico | 2010-2020 | 5 |
| Moldova | 1980-1984 | 5 |
| Moldova | 1985-1989 | 5 |
| Moldova | 1990-1994 | 4 |
| Moldova | 1995-1999 | 5 |
| Moldova | 2000-2004 | 5 |
| Moldova | 2005-2009 | 5 |
| Moldova | 2010-2020 | 5 |
| Monaco | 1980-1984 | 0 |
| Monaco | 1985-1989 | 4 |
| Monaco | 1990-1994 | 0 |
| Monaco | 1995-1999 | 0 |
| Monaco | 2000-2004 | 0 |
| Monaco | 2005-2009 | 0 |
| Monaco | 2010-2020 | 4 |
| Mongolia | 1980-1984 | 0 |
| Mongolia | 1985-1989 | 0 |
| Mongolia | 1990-1994 | 3 |
| Mongolia | 1995-1999 | 0 |
| Mongolia | 2000-2004 | 2 |
| Mongolia | 2005-2009 | 2 |
| Mongolia | 2010-2020 | 4 |
| Montenegro | 1980-1984 | 0 |
| Montenegro | 1985-1989 | 0 |
| Montenegro | 1990-1994 | 0 |
| Montenegro | 1995-1999 | 0 |
| Montenegro | 2000-2004 | 4 |
| Montenegro | 2005-2009 | 3 |
| Montenegro | 2010-2020 | 0 |
| Morocco | 1980-1984 | 0 |
| Morocco | 1985-1989 | 2 |
| Morocco | 1990-1994 | 0 |
| Morocco | 1995-1999 | 0 |
| Morocco | 2000-2004 | 2 |
| Morocco | 2005-2009 | 2 |
| Morocco | 2010-2020 | 2 |
| Mozambique | 1980-1984 | 0 |
| Mozambique | 1985-1989 | 0 |
| Mozambique | 1990-1994 | 0 |
| Mozambique | 1995-1999 | 1 |
| Mozambique | 2000-2004 | 2 |
| Mozambique | 2005-2009 | 3 |
| Mozambique | 2010-2020 | 1 |
| Myanmar | 1980-1984 | 0 |
| Myanmar | 1985-1989 | 0 |
| Myanmar | 1990-1994 | 0 |
| Myanmar | 1995-1999 | 0 |
| Myanmar | 2000-2004 | 0 |
| Myanmar | 2005-2009 | 1 |
| Myanmar | 2010-2020 | 3 |
| Namibia | 1980-1984 | 0 |
| Namibia | 1985-1989 | 0 |
| Namibia | 1990-1994 | 0 |
| Namibia | 1995-1999 | 0 |
| Namibia | 2000-2004 | 0 |
| Namibia | 2005-2009 | 0 |
| Namibia | 2010-2020 | 0 |
| Nauru | 1980-1984 | 0 |
| Nauru | 1985-1989 | 0 |
| Nauru | 1990-1994 | 0 |
| Nauru | 1995-1999 | 0 |
| Nauru | 2000-2004 | 0 |
| Nauru | 2005-2009 | 0 |
| Nauru | 2010-2020 | 0 |
| Nepal | 1980-1984 | 1 |
| Nepal | 1985-1989 | 1 |
| Nepal | 1990-1994 | 0 |
| Nepal | 1995-1999 | 1 |
| Nepal | 2000-2004 | 1 |
| Nepal | 2005-2009 | 3 |
| Nepal | 2010-2020 | 1 |
| Netherlands | 1980-1984 | 5 |
| Netherlands | 1985-1989 | 5 |
| Netherlands | 1990-1994 | 5 |
| Netherlands | 1995-1999 | 5 |
| Netherlands | 2000-2004 | 4 |
| Netherlands | 2005-2009 | 5 |
| Netherlands | 2010-2020 | 5 |
| New Zealand | 1980-1984 | 5 |
| New Zealand | 1985-1989 | 5 |
| New Zealand | 1990-1994 | 5 |
| New Zealand | 1995-1999 | 5 |
| New Zealand | 2000-2004 | 5 |
| New Zealand | 2005-2009 | 5 |
| New Zealand | 2010-2020 | 5 |
| Nicaragua | 1980-1984 | 0 |
| Nicaragua | 1985-1989 | 3 |
| Nicaragua | 1990-1994 | 3 |
| Nicaragua | 1995-1999 | 4 |
| Nicaragua | 2000-2004 | 4 |
| Nicaragua | 2005-2009 | 4 |
| Nicaragua | 2010-2020 | 5 |
| Niger | 1980-1984 | 0 |
| Niger | 1985-1989 | 0 |
| Niger | 1990-1994 | 0 |
| Niger | 1995-1999 | 0 |
| Niger | 2000-2004 | 0 |
| Niger | 2005-2009 | 3 |
| Niger | 2010-2020 | 0 |
| Nigeria | 1980-1984 | 0 |
| Nigeria | 1985-1989 | 0 |
| Nigeria | 1990-1994 | 1 |
| Nigeria | 1995-1999 | 1 |
| Nigeria | 2000-2004 | 0 |
| Nigeria | 2005-2009 | 1 |
| Nigeria | 2010-2020 | 3 |
| Niue | 1980-1984 | 0 |
| Niue | 1985-1989 | 0 |
| Niue | 1990-1994 | 0 |
| Niue | 1995-1999 | 0 |
| Niue | 2000-2004 | 0 |
| Niue | 2005-2009 | 0 |
| Niue | 2010-2020 | 0 |
| North Korea | 1980-1984 | 0 |
| North Korea | 1985-1989 | 0 |
| North Korea | 1990-1994 | 0 |
| North Korea | 1995-1999 | 0 |
| North Korea | 2000-2004 | 0 |
| North Korea | 2005-2009 | 0 |
| North Korea | 2010-2020 | 0 |
| North Macedonia | 1980-1984 | 0 |
| North Macedonia | 1985-1989 | 0 |
| North Macedonia | 1990-1994 | 4 |
| North Macedonia | 1995-1999 | 4 |
| North Macedonia | 2000-2004 | 4 |
| North Macedonia | 2005-2009 | 4 |
| North Macedonia | 2010-2020 | 4 |
| Northern Mariana Islands | 1980-1984 | 0 |
| Northern Mariana Islands | 1985-1989 | 0 |
| Northern Mariana Islands | 1990-1994 | 0 |
| Northern Mariana Islands | 1995-1999 | 3 |
| Northern Mariana Islands | 2000-2004 | 3 |
| Northern Mariana Islands | 2005-2009 | 3 |
| Northern Mariana Islands | 2010-2020 | 3 |
| Norway | 1980-1984 | 5 |
| Norway | 1985-1989 | 5 |
| Norway | 1990-1994 | 5 |
| Norway | 1995-1999 | 5 |
| Norway | 2000-2004 | 5 |
| Norway | 2005-2009 | 5 |
| Norway | 2010-2020 | 5 |
| Oman | 1980-1984 | 0 |
| Oman | 1985-1989 | 0 |
| Oman | 1990-1994 | 0 |
| Oman | 1995-1999 | 0 |
| Oman | 2000-2004 | 0 |
| Oman | 2005-2009 | 3 |
| Oman | 2010-2020 | 2 |
| Pakistan | 1980-1984 | 0 |
| Pakistan Pakistan | 1985-1989 1990-1994 | 2 1 |
| Pakistan | 1995-1999 | 0 |
| Pakistan | 2000-2004 | 2 |
| Pakistan | 2005-2009 | 2 |
| Pakistan | 2010-2020 | 2 |
| Palau | 1980-1984 | 0 |
| Palau | 1985-1989 | 0 |
| Palau | 1990-1994 | 0 |
| Palau | 1995-1999 | 0 |
| Palau | 2000-2004 | 0 |
| Palau | 2005-2009 | 0 |
| Palau | 2010-2020 | 3 |
| Palestine | 1980-1984 | 0 |
| Palestine | 1985-1989 | 0 |
| Palestine | 1990-1994 | 0 |
| Palestine | 1995-1999 | 2 |
| Palestine | 2000-2004 | 2 |
| Palestine | 2005-2009 | 2 |
| Palestine | 2010-2020 | 4 |
| Panama | 1980-1984 | 4 |
| Panama | 1985-1989 | 4 |
| Panama | 1990-1994 | 0 |
| Panama | 1995-1999 | 4 |
| Panama | 2000-2004 | 5 |
| Panama | 2005-2009 | 5 |
| Panama | 2010-2020 | 4 |
| Papua New Guinea | 1980-1984 | 1 |
| Papua New Guinea | 1985-1989 | 1 |
| Papua New Guinea | 1990-1994 | 0 |
| Papua New Guinea | 1995-1999 | 0 |
| Papua New Guinea | 2000-2004 | 0 |
| Papua New Guinea | 2005-2009 | 0 |
| Papua New Guinea | 2010-2020 | 2 |
| Paraguay | 1980-1984 | 3 |
| Paraguay | 1985-1989 | 3 |
| Paraguay | 1990-1994 | 3 |
| Paraguay | 1995-1999 | 3 |
| Paraguay | 2000-2004 | 3 |
| Paraguay | 2005-2009 | 4 |
| Paraguay | 2010-2020 | 4 |
| Peru | 1980-1984 | 3 |
| Peru | 1985-1989 | 2 |
| Peru | 1990-1994 | 3 |
| Peru | 1995-1999 | 3 |
| Peru | 2000-2004 | 3 |
| Peru | 2005-2009 | 3 |
| Peru | 2010-2020 | 3 |
| Philippines | 1980-1984 | 1 |
| Philippines | 1985-1989 | 2 |
| Philippines | 1990-1994 | 2 |
| Philippines | 1995-1999 | 3 |
| Philippines | 2000-2004 | 3 |
| Philippines | 2005-2009 | 4 |
| Philippines | 2010-2020 | 4 |
| Poland | 1980-1984 | 3 |
| Poland | 1985-1989 | 3 |
| Poland | 1990-1994 | 3 |
| Poland | 1995-1999 | 4 |
| Poland | 2000-2004 | 4 |
| Poland | 2005-2009 | 4 |
| Poland | 2010-2020 | 4 |
| Portugal | 1980-1984 | 4 |
| Portugal | 1985-1989 | 4 |
| Portugal | 1990-1994 | 4 |
| Portugal | 1995-1999 | 4 |
| Portugal | 2000-2004 | 4 |
| Portugal | 2005-2009 | 4 |
| Portugal | 2010-2020 | 5 |
| Puerto Rico | 1980-1984 | 4 |
| Puerto Rico | 1985-1989 | 4 |
| Puerto Rico | 1990-1994 | 4 |
| Puerto Rico | 1995-1999 | 4 |
| Puerto Rico | 2000-2004 | 4 |
| Puerto Rico | 2005-2009 | 4 |
| Puerto Rico | 2010-2020 | 4 |
| Qatar | 1980-1984 | 2 |
| Qatar | 1985-1989 | 2 |
| Qatar | 1990-1994 | 0 |
| Qatar | 1995-1999 | 3 |
| Qatar | 2000-2004 | 3 |
| Qatar | 2005-2009 | 3 |
| Qatar | 2010-2020 | 3 |
| Romania | 1980-1984 | 4 |
| Romania | 1985-1989 | 4 |
| Romania | 1990-1994 | 4 |
| Romania | 1995-1999 | 5 |
| Romania | 2000-2004 | 5 |
| Romania | 2005-2009 | 5 |
| Romania | 2010-2020 | 5 |
| Russia | 1980-1984 | 5 |
| Russia | 1985-1989 | 5 |
| Russia | 1990-1994 | 5 |
| Russia | 1995-1999 | 5 |
| Russia | 2000-2004 | 5 |
| Russia | 2005-2009 | 5 |
| Russia | 2010-2020 | 5 |
| Rwanda | 1980-1984 | 0 |
| Rwanda | 1985-1989 | 0 |
| Rwanda | 1990-1994 | 0 |
| Rwanda | 1995-1999 | 0 |
| Rwanda | 2000-2004 | 0 |
| Rwanda | 2005-2009 | 2 |
| Rwanda | 2010-2020 | 1 |
| Saint Kitts and Nevis | 1980-1984 | 4 |
| Saint Kitts and Nevis | 1985-1989 | 3 |
| Saint Kitts and Nevis | 1990-1994 | 4 |
| Saint Kitts and Nevis | 1995-1999 | 4 |
| Saint Kitts and Nevis | 2000-2004 | 4 |
| Saint Kitts and Nevis | 2005-2009 | 5 |
| Saint Kitts and Nevis | 2010-2020 | 5 |
| Saint Lucia | 1980-1984 | 4 |
| Saint Lucia | 1985-1989 | 4 |
| Saint Lucia | 1990-1994 | 4 |
| Saint Lucia | 1995-1999 | 4 |
| Saint Lucia | 2000-2004 | 4 |
| Saint Lucia | 2005-2009 | 4 |
| Saint Lucia | 2010-2020 | 5 |
| Saint Vincent and the Grenadines | 1980-1984 | 4 |
| Saint Vincent and the Grenadines | 1985-1989 | 3 |
| Saint Vincent and the Grenadines | 1990-1994 | 3 |
| Saint Vincent and the Grenadines | 1995-1999 | 5 |
| Saint Vincent and the Grenadines | 2000-2004 | 5 |
| Saint Vincent and the Grenadines | 2005-2009 | 5 |
| Saint Vincent and the Grenadines | 2010-2020 | 5 |
| Samoa | 1980-1984 | 0 |
| Samoa | 1985-1989 | 0 |
| Samoa | 1990-1994 | 0 |
| Samoa | 1995-1999 | 0 |
| Samoa | 2000-2004 | 0 |
| Samoa | 2005-2009 | 0 |
| Samoa | 2010-2020 | 0 |
| San Marino | 1980-1984 | 0 |
| San Marino | 1985-1989 | 0 |
| San Marino | 1990-1994 | 0 |
| San Marino | 1995-1999 | 4 |
| San Marino | 2000-2004 | 4 |
| San Marino | 2005-2009 | 4 |
| San Marino | 2010-2020 | 4 |
| Saudi Arabia | 1980-1984 | 0 |
| Saudi Arabia | 1985-1989 | 0 |
| Saudi Arabia | 1990-1994 | 0 |
| Saudi Arabia | 1995-1999 | 2 |
| Saudi Arabia | 2000-2004 | 2 |
| Saudi Arabia | 2005-2009 | 2 |
| Saudi Arabia | 2010-2020 | 2 |
| Senegal | 1980-1984 | 1 |
| Senegal | 1985-1989 | 1 |
| Senegal | 1990-1994 | 1 |
| Senegal | 1995-1999 | 1 |
| Senegal | 2000-2004 | 0 |
| Senegal | 2005-2009 | 1 |
| Senegal | 2010-2020 | 1 |
| Serbia | 1980-1984 | 0 |
| Serbia | 1985-1989 | 0 |
| Serbia | 1990-1994 | 0 |
| Serbia | 1995-1999 | 4 |
| Serbia | 2000-2004 | 4 |
| Serbia | 2005-2009 | 4 |
| Serbia | 2010-2020 | 4 |
| Seychelles | 1980-1984 | 4 |
| Seychelles | 1985-1989 | 4 |
| Seychelles | 1990-1994 | 0 |
| Seychelles | 1995-1999 | 0 |
| Seychelles | 2000-2004 | 4 |
| Seychelles | 2005-2009 | 4 |
| Seychelles | 2010-2020 | 4 |
| Sierra Leone | 1980-1984 | 0 |
| Sierra Leone | 1985-1989 | 0 |
| Sierra Leone | 1990-1994 | 1 |
| Sierra Leone | 1995-1999 | 0 |
| Sierra Leone | 2000-2004 | 0 |
| Sierra Leone | 2005-2009 | 0 |
| Sierra Leone | 2010-2020 | 1 |
| Singapore | 1980-1984 | 5 |
| Singapore | 1985-1989 | 5 |
| Singapore | 1990-1994 | 5 |
| Singapore | 1995-1999 | 5 |
| Singapore | 2000-2004 | 5 |
| Singapore | 2005-2009 | 5 |
| Singapore | 2010-2020 | 5 |
| Slovakia | 1980-1984 | 0 |
| Slovakia | 1985-1989 | 0 |
| Slovakia | 1990-1994 | 4 |
| Slovakia | 1995-1999 | 4 |
| Slovakia | 2000-2004 | 5 |
| Slovakia | 2005-2009 | 5 |
| Slovakia | 2010-2020 | 5 |
| Slovenia | 1980-1984 | 0 |
| Slovenia | 1985-1989 | 5 |
| Slovenia | 1990-1994 | 5 |
| Slovenia | 1995-1999 | 5 |
| Slovenia | 2000-2004 | 5 |
| Slovenia | 2005-2009 | 5 |
| Slovenia | 2010-2020 | 5 |
| Solomon Islands | 1980-1984 | 0 |
| Solomon Islands | 1985-1989 | 0 |
| Solomon Islands | 1990-1994 | 0 |
| Solomon Islands | 1995-1999 | 0 |
| Solomon Islands | 2000-2004 | 0 |
| Solomon Islands | 2005-2009 | 0 |
| Solomon Islands | 2010-2020 | 3 |
| Somalia | 1980-1984 | 0 |
| Somalia | 1985-1989 | 0 |
| Somalia | 1990-1994 | 0 |
| Somalia | 1995-1999 | 0 |
| Somalia | 2000-2004 | 0 |
| Somalia | 2005-2009 | 0 |
| Somalia | 2010-2020 | 0 |
| South Africa | 1980-1984 | 0 |
| South Africa | 1985-1989 | 0 |
| South Africa | 1990-1994 | 1 |
| South Africa | 1995-1999 | 4 |
| South Africa | 2000-2004 | 4 |
| South Africa | 2005-2009 | 4 |
| South Africa | 2010-2020 | 4 |
| South Korea | 1980-1984 | 0 |
| South Korea | 1985-1989 | 3 |
| South Korea | 1990-1994 | 4 |
| South Korea | 1995-1999 | 4 |
| South Korea | 2000-2004 | 4 |
| South Korea | 2005-2009 | 4 |
| South Korea | 2010-2020 | 4 |
| South Sudan | 1980-1984 | 0 |
| South Sudan | 1985-1989 | 0 |
| South Sudan | 1990-1994 | 0 |
| South Sudan | 1995-1999 | 0 |
| South Sudan | 2000-2004 | 0 |
| South Sudan | 2005-2009 | 0 |
| South Sudan | 2010-2020 | 0 |
| Spain | 1980-1984 | 4 |
| Spain | 1985-1989 | 4 |
| Spain | 1990-1994 | 5 |
| Spain | 1995-1999 | 5 |
| Spain | 2000-2004 | 5 |
| Spain | 2005-2009 | 5 |
| Spain | 2010-2020 | 5 |
| Sri Lanka | 1980-1984 | 3 |
| Sri Lanka | 1985-1989 | 3 |
| Sri Lanka | 1990-1994 | 3 |
| Sri Lanka | 1995-1999 | 3 |
| Sri Lanka | 2000-2004 | 3 |
| Sri Lanka | 2005-2009 | 4 |
| Sri Lanka | 2010-2020 | 3 |
| Sudan | 1980-1984 | 0 |
| Sudan | 1985-1989 | 0 |
| Sudan | 1990-1994 | 0 |
| Sudan | 1995-1999 | 0 |
| Sudan | 2000-2004 | 0 |
| Sudan | 2005-2009 | 0 |
| Sudan | 2010-2020 | 0 |
| Suriname | 1980-1984 | 3 |
| Suriname | 1985-1989 | 3 |
| Suriname | 1990-1994 | 3 |
| Suriname | 1995-1999 | 3 |
| Suriname | 2000-2004 | 4 |
| Suriname | 2005-2009 | 4 |
| Suriname | 2010-2020 | 4 |
| Sweden | 1980-1984 | 5 |
| Sweden | 1985-1989 | 5 |
| Sweden | 1990-1994 | 5 |
| Sweden | 1995-1999 | 5 |
| Sweden | 2000-2004 | 5 |
| Sweden | 2005-2009 | 5 |
| Sweden | 2010-2020 | 5 |
| Switzerland | 1980-1984 | 4 |
| Switzerland | 1985-1989 | 4 |
| Switzerland | 1990-1994 | 4 |
| Switzerland | 1995-1999 | 5 |
| Switzerland | 2000-2004 | 5 |
| Switzerland | 2005-2009 | 5 |
| Switzerland | 2010-2020 | 5 |
| Syria | 1980-1984 | 2 |
| Syria | 1985-1989 | 2 |
| Syria | 1990-1994 | 0 |
| Syria | 1995-1999 | 3 |
| Syria | 2000-2004 | 3 |
| Syria | 2005-2009 | 4 |
| Syria | 2010-2020 | 3 |
| São Tomé and Príncipe | 1980-1984 | 0 |
| São Tomé and Príncipe | 1985-1989 | 3 |
| São Tomé and Príncipe | 1990-1994 | 0 |
| São Tomé and Príncipe | 1995-1999 | 0 |
| São Tomé and Príncipe | 2000-2004 | 0 |
| São Tomé and Príncipe | 2005-2009 | 0 |
| São Tomé and Príncipe | 2010-2020 | 0 |
| Taiwan (province of China) | 1980-1984 | 4 |
| Taiwan (province of China) | 1985-1989 | 4 |
| Taiwan (province of China) | 1990-1994 | 4 |
| Taiwan (province of China) | 1995-1999 | 4 |
| Taiwan (province of China) | 2000-2004 | 4 |
| Taiwan (province of China) | 2005-2009 | 5 |
| Taiwan (province of China) | 2010-2020 | 5 |
| Tajikistan | 1980-1984 | 4 |
| Tajikistan | 1985-1989 | 4 |
| Tajikistan | 1990-1994 | 4 |
| Tajikistan | 1995-1999 | 3 |
| Tajikistan | 2000-2004 | 3 |
| Tajikistan | 2005-2009 | 3 |
| Tajikistan | 2010-2020 | 3 |
| Tanzania | 1980-1984 | 0 |
| Tanzania | 1985-1989 | 1 |
| Tanzania | 1990-1994 | 1 |
| Tanzania | 1995-1999 | 1 |
| Tanzania | 2000-2004 | 1 |
| Tanzania | 2005-2009 | 1 |
| Tanzania | 2010-2020 | 1 |
| Thailand | 1980-1984 | 2 |
| Thailand | 1985-1989 | 2 |
| Thailand | 1990-1994 | 2 |
| Thailand | 1995-1999 | 3 |
| Thailand | 2000-2004 | 3 |
| Thailand | 2005-2009 | 3 |
| Thailand | 2010-2020 | 4 |
| The Bahamas | 1980-1984 | 4 |
| The Bahamas | 1985-1989 | 4 |
| The Bahamas | 1990-1994 | 4 |
| The Bahamas | 1995-1999 | 5 |
| The Bahamas | 2000-2004 | 5 |
| The Bahamas | 2005-2009 | 5 |
| The Bahamas | 2010-2020 | 5 |
| The Gambia | 1980-1984 | 1 |
| The Gambia | 1985-1989 | 1 |
| The Gambia | 1990-1994 | 1 |
| The Gambia | 1995-1999 | 1 |
| The Gambia | 2000-2004 | 1 |
| The Gambia | 2005-2009 | 1 |
| The Gambia | 2010-2020 | 0 |
| Timor-Leste | 1980-1984 | 0 |
| Timor-Leste | 1985-1989 | 0 |
| Timor-Leste | 1990-1994 | 0 |
| Timor-Leste | 1995-1999 | 0 |
| Timor-Leste | 2000-2004 | 0 |
| Timor-Leste | 2005-2009 | 0 |
| Timor-Leste | 2010-2020 | 0 |
| Togo | 1980-1984 | 0 |
| Togo | 1985-1989 | 0 |
| Togo | 1990-1994 | 0 |
| Togo | 1995-1999 | 0 |
| Togo | 2000-2004 | 0 |
| Togo | 2005-2009 | 0 |
| Togo | 2010-2020 | 0 |
| Tokelau | 1980-1984 | 0 |
| Tokelau | 1985-1989 | 0 |
| Tokelau | 1990-1994 | 0 |
| Tokelau | 1995-1999 | 0 |
| Tokelau | 2000-2004 | 0 |
| Tokelau | 2005-2009 | 0 |
| Tokelau | 2010-2020 | 0 |
| Tonga | 1980-1984 | 0 |
| Tonga | 1985-1989 | 0 |
| Tonga | 1990-1994 | 0 |
| Tonga | 1995-1999 | 0 |
| Tonga | 2000-2004 | 3 |
| Tonga | 2005-2009 | 0 |
| Tonga | 2010-2020 | 0 |
| Trinidad and Tobago | 1980-1984 | 4 |
| Trinidad and Tobago | 1985-1989 | 4 |
| Trinidad and Tobago | 1990-1994 | 5 |
| Trinidad and Tobago | 1995-1999 | 5 |
| Trinidad and Tobago | 2000-2004 | 5 |
| Trinidad and Tobago | 2005-2009 | 5 |
| Trinidad and Tobago | 2010-2020 | 5 |
| Tunisia | 1980-1984 | 0 |
| Tunisia | 1985-1989 | 0 |
| Tunisia | 1990-1994 | 0 |
| Tunisia | 1995-1999 | 0 |
| Tunisia | 2000-2004 | 0 |
| Tunisia | 2005-2009 | 2 |
| Tunisia | 2010-2020 | 2 |
| Turkey | 1980-1984 | 2 |
| Turkey | 1985-1989 | 2 |
| Turkey | 1990-1994 | 2 |
| Turkey | 1995-1999 | 2 |
| Turkey | 2000-2004 | 3 |
| Turkey | 2005-2009 | 3 |
| Turkey | 2010-2020 | 4 |
| Turkmenistan | 1980-1984 | 5 |
| Turkmenistan | 1985-1989 | 5 |
| Turkmenistan | 1990-1994 | 5 |
| Turkmenistan | 1995-1999 | 4 |
| Turkmenistan | 2000-2004 | 4 |
| Turkmenistan | 2005-2009 | 4 |
| Turkmenistan | 2010-2020 | 4 |
| Tuvalu | 1980-1984 | 0 |
| Tuvalu | 1985-1989 | 0 |
| Tuvalu | 1990-1994 | 0 |
| Tuvalu | 1995-1999 | 0 |
| Tuvalu | 2000-2004 | 0 |
| Tuvalu | 2005-2009 | 0 |
| Tuvalu | 2010-2020 | 0 |
| UK | 1980-1984 | 5 |
| UK | 1985-1989 | 5 |
| UK | 1990-1994 | 5 |
| UK | 1995-1999 | 5 |
| UK | 2000-2004 | 5 |
| UK | 2005-2009 | 5 |
| UK | 2010-2020 | 5 |
| USA | 1980-1984 | 5 |
| USA | 1985-1989 | 5 |
| USA | 1990-1994 | 5 |
| USA | 1995-1999 | 5 |
| USA | 2000-2004 | 5 |
| USA | 2005-2009 | 5 |
| USA | 2010-2020 | 5 |
| Uganda | 1980-1984 | 0 |
| Uganda | 1985-1989 | 0 |
| Uganda | 1990-1994 | 0 |
| Uganda | 1995-1999 | 0 |
| Uganda | 2000-2004 | 1 |
| Uganda | 2005-2009 | 1 |
| Uganda | 2010-2020 | 1 |
| Ukraine | 1980-1984 | 5 |
| Ukraine | 1985-1989 | 5 |
| Ukraine | 1990-1994 | 4 |
| Ukraine | 1995-1999 | 4 |
| Ukraine | 2000-2004 | 4 |
| Ukraine | 2005-2009 | 5 |
| Ukraine | 2010-2020 | 5 |
| United Arab Emirates | 1980-1984 | 0 |
| United Arab Emirates | 1985-1989 | 0 |
| United Arab Emirates | 1990-1994 | 0 |
| United Arab Emirates | 1995-1999 | 0 |
| United Arab Emirates | 2000-2004 | 0 |
| United Arab Emirates | 2005-2009 | 3 |
| United Arab Emirates | 2010-2020 | 2 |
| Uruguay | 1980-1984 | 4 |
| Uruguay | 1985-1989 | 4 |
| Uruguay | 1990-1994 | 4 |
| Uruguay | 1995-1999 | 4 |
| Uruguay | 2000-2004 | 4 |
| Uruguay | 2005-2009 | 4 |
| Uruguay | 2010-2020 | 4 |
| Uzbekistan | 1980-1984 | 5 |
| Uzbekistan | 1985-1989 | 5 |
| Uzbekistan | 1990-1994 | 4 |
| Uzbekistan | 1995-1999 | 4 |
| Uzbekistan | 2000-2004 | 3 |
| Uzbekistan | 2005-2009 | 3 |
| Uzbekistan | 2010-2020 | 3 |
| Vanuatu | 1980-1984 | 0 |
| Vanuatu | 1985-1989 | 0 |
| Vanuatu | 1990-1994 | 0 |
| Vanuatu | 1995-1999 | 0 |
| Vanuatu | 2000-2004 | 0 |
| Vanuatu | 2005-2009 | 0 |
| Vanuatu | 2010-2020 | 0 |
| Venezuela | 1980-1984 | 4 |
| Venezuela | 1985-1989 | 4 |
| Venezuela | 1990-1994 | 4 |
| Venezuela | 1995-1999 | 5 |
| Venezuela | 2000-2004 | 5 |
| Venezuela | 2005-2009 | 5 |
| Venezuela | 2010-2020 | 5 |
| Vietnam | 1980-1984 | 0 |
| Vietnam | 1985-1989 | 1 |
| Vietnam | 1990-1994 | 1 |
| Vietnam | 1995-1999 | 1 |
| Vietnam | 2000-2004 | 1 |
| Vietnam | 2005-2009 | 3 |
| Vietnam | 2010-2020 | 1 |
| Virgin Islands | 1980-1984 | 4 |
| Virgin Islands | 1985-1989 | 0 |
| Virgin Islands | 1990-1994 | 4 |
| Virgin Islands | 1995-1999 | 5 |
| Virgin Islands | 2000-2004 | 4 |
| Virgin Islands | 2005-2009 | 4 |
| Virgin Islands | 2010-2020 | 3 |
| Yemen | 1980-1984 | 0 |
| Yemen | 1985-1989 | 0 |
| Yemen | 1990-1994 | 0 |
| Yemen | 1995-1999 | 0 |
| Yemen | 2000-2004 | 0 |
| Yemen | 2005-2009 | 0 |
| Yemen | 2010-2020 | 0 |
| Zambia | 1980-1984 | 0 |
| Zambia | 1985-1989 | 0 |
| Zambia | 1990-1994 | 0 |
| Zambia | 1995-1999 | 0 |
| Zambia | 2000-2004 | 0 |
| Zambia | 2005-2009 | 1 |
| Zambia | 2010-2020 | 2 |
| Zimbabwe | 1980-1984 | 0 |
| Zimbabwe | 1985-1989 | 0 |
| Zimbabwe | 1990-1994 | 3 |
| Zimbabwe | 1995-1999 | 3 |
| Zimbabwe | 2000-2004 | 1 |
| Zimbabwe | 2005-2009 | 3 |
| Zimbabwe | 2010-2020 | 0 |

**The difference between GBD and Global Cancer Observatory (GLOBOCAN)**

The Global Cancer Observatory (GLOBOCAN) 2020 and Global Burden of Disease (GBD) 2019 are both essential resources for global cancer statistics but differ in methods, sources, and data coverage[12]. GLOBOCAN's data primarily relies on national population-based cancer registries and cancer registry networks, while GBD draws from a broader range of sources, including survey data and hospital records, which can vary in quality and consistency. GLOBOCAN uses stable population models and assumes a relationship between cancer incidence and mortality, often extrapolating data in areas with limited cancer registries. In contrast, GBD utilizes a multi-source aggregation and adjusts for data gaps, which can lead to differences in estimated incidence and mortality. Another key difference lies in coding: GBD includes a broader range of biliary tract cancers under gallbladder cancer (ICD-10 C23-C24), while GLOBOCAN restricts this to C23 only, which can result in varied cancer burden assessments across databases, especially in countries with inconsistent cancer reporting systems[12].

**References**

1. GBD 2021 Diseases and Injuries Collaborators. Global incidence, prevalence, years lived with disability (YLDs), disability-adjusted life-years (DALYs), and healthy life expectancy (HALE) for 371 diseases and injuries in 204 countries and territories and 811 subnational locations, 1990-2021: a systematic analysis for the Global Burden of Disease Study 2021. Lancet. 2024;403:2133–61.

2. GBD 2019 Colorectal Cancer Collaborators. Global, regional, and national burden of colorectal cancer and its risk factors, 1990-2019: a systematic analysis for the Global Burden of Disease Study 2019. Lancet Gastroenterol Hepatol. 2022;7:627–47.

3. GBD 2021 Appendicitis Collaborator Group. Trends and levels of the global, regional, and national burden of appendicitis between 1990 and 2021: findings from the Global Burden of Disease Study 2021. Lancet Gastroenterol Hepatol. 2024;:S2468-1253(24)00157-2.

4. Baidoun F, Elshiwy K, Elkeraie Y, Merjaneh Z, Khoudari G, Sarmini MT, et al. Colorectal Cancer Epidemiology: Recent Trends and Impact on Outcomes. Curr Drug Targets. 2021;22:998–1009.

5. Ferris JS, Prest MT, Hur C, Chen L, Elkin EB, Melamed A, et al. Trends in uterine cancer incidence in the United States: The contribution of age, period and cohort effects. Gynecol Oncol. 2024;187:151–62.

6. Rosenberg PS, Check DP, Anderson WF. A web tool for age-period-cohort analysis of cancer incidence and mortality rates. Cancer Epidemiol Biomarkers Prev. 2014;23:2296–302.

7. Das Gupta P. Standardization and decomposition of rates from cross-classified data. Genus. 1994;50:171–96.

8. Liu C, Zhu S, Zhang J, Wu P, Wang X, Du S, et al. Global, regional, and national burden of liver cancer due to non-alcoholic steatohepatitis, 1990-2019: a decomposition and age-period-cohort analysis. J Gastroenterol. 2023;58:1222–36.

9. Cheng X, Yang Y, Schwebel DC, Liu Z, Li L, Cheng P, et al. Population ageing and mortality during 1990-2017: A global decomposition analysis. PLoS Med. 2020;17:e1003138.

10. Organization WH. Handbook on Health Inequality Monitoring: With a Special Focus on Low- and Middle-income Countries. World Health Organization; 2013.

11. Collaborators G 2021 C of D. Global burden of 288 causes of death and life expectancy decomposition in 204 countries and territories and 811 subnational locations, 1990–2021: a systematic analysis for the Global Burden of Disease Study 2021. Lancet (London, England). 2024;403:2100.

12. Yu Z, Bai X, Zhou R, Ruan G, Guo M, Han W, et al. Differences in the incidence and mortality of digestive cancer between Global Cancer Observatory 2020 and Global Burden of Disease 2019. Int J Cancer. 2024;154:615–25.

| Supplementary Table 1. Global and regional AAPC for ASR of GBTC incidence, deaths, and DALYs from 1990 to 2021 | | | | | | |
| --- | --- | --- | --- | --- | --- | --- |
| Location | Incidence | | Death | | DALYs | |
|  | AAPC (95%CI) | *P* value | AAPC (95%CI) | *P* value | AAPC (95%CI) | *P* value |
| Global | -0.388 (-0.495 to -0.282) | <0.001 | -0.878 (-0.96 to -0.795) | <0.001 | -0.972 (-1.068 to -0.875) | <0.001 |
| **Sex** |  |  |  |  |  |  |
| Male | 0.229 (0.078 to 0.381) | 0.003 | -0.363 (-0.46 to -0.266) | <0.001 | -0.531 (-0.628 to -0.435) | <0.001 |
| Female | -0.817 (-0.94 to -0.695) | <0.001 | -1.205 (-1.308 to -1.101) | <0.001 | -1.273 (-1.343 to -1.202) | <0.001 |
| **Region** |  |  |  |  |  |  |
| Central Asia | -0.817 (-1.607 to -0.02) | 0.045 | -0.837 (-1.639 to -0.029) | 0.042 | -0.997 (-1.753 to -0.234) | 0.01 |
| Central Europe | -1.53 (-1.733 to -1.327) | <0.001 | -1.933 (-2.135 to -1.731) | <0.001 | -1.944 (-2.153 to -1.735) | <0.001 |
| Eastern Europe | 0.275 (-0.794 to 1.356) | 0.616 | -0.469 (-1.247 to 0.315) | 0.24 | -0.644 (-1.639 to 0.362) | 0.209 |
| Australasia | -0.06 (-0.987 to 0.877) | 0.901 | -1.574 (-1.833 to -1.315) | <0.001 | -1.623 (-1.888 to -1.358) | <0.001 |
| High-income Asia Pacific | -1.244 (-1.435 to -1.054) | <0.001 | -1.89 (-2.003 to -1.777) | <0.001 | -2.296 (-2.427 to -2.166) | <0.001 |
| High-income North America | -0.513 (-0.819 to -0.208) | 0.001 | -1.448 (-1.759 to -1.137) | <0.001 | -1.349 (-1.449 to -1.249) | <0.001 |
| Southern Latin America | -1.737 (-2.034 to -1.439) | <0.001 | -2.209 (-2.522 to -1.896) | <0.001 | -2.132 (-2.492 to -1.771) | <0.001 |
| Western Europe | -1.148 (-1.301 to -0.994) | <0.001 | -2.184 (-2.376 to -1.992) | <0.001 | -2.3 (-2.469 to -2.13) | <0.001 |
| Andean Latin America | -0.678 (-1.146 to -0.208) | 0.005 | -0.9 (-1.379 to -0.418) | <0.001 | -1.002 (-1.449 to -0.552) | <0.001 |
| Caribbean | -1.612 (-1.797 to -1.427) | <0.001 | -1.786 (-1.968 to -1.604) | <0.001 | -1.686 (-1.859 to -1.512) | <0.001 |
| Central Latin America | -2.16 (-2.522 to -1.797) | <0.001 | -2.521 (-2.822 to -2.218) | <0.001 | -2.516 (-2.835 to -2.195) | <0.001 |
| Tropical Latin America | -1.009 (-1.22 to -0.797) | <0.001 | -1.194 (-1.389 to -0.998) | <0.001 | -1.121 (-1.352 to -0.889) | <0.001 |
| North Africa and Middle East | -0.007 (-0.069 to 0.055) | 0.831 | -0.302 (-0.376 to -0.228) | <0.001 | -0.462 (-0.53 to -0.394) | <0.001 |
| South Asia | 1.027 (0.781 to 1.274) | <0.001 | 0.961 (0.657 to 1.265) | <0.001 | 0.824 (0.636 to 1.013) | <0.001 |
| East Asia | 0.399 (0.206 to 0.592) | <0.001 | -0.745 (-1.062 to -0.427) | <0.001 | -0.865 (-1.109 to -0.621) | <0.001 |
| Oceania | -0.652 (-0.836 to -0.468) | <0.001 | -0.671 (-0.766 to -0.576) | <0.001 | -0.713 (-0.884 to -0.543) | <0.001 |
| Southeast Asia | 0.451 (0.377 to 0.525) | <0.001 | 0.127 (0.052 to 0.202) | 0.001 | -0.089 (-0.18 to 0.002) | 0.055 |
| Central Sub-Saharan Africa | 0.218 (0.125 to 0.31) | <0.001 | 0.211 (0.121 to 0.301) | <0.001 | 0.122 (0.057 to 0.187) | <0.001 |
| Eastern Sub-Saharan Africa | -0.285 (-0.349 to -0.221) | <0.001 | -0.265 (-0.322 to -0.208) | <0.001 | -0.49 (-0.551 to -0.428) | <0.001 |
| Southern Sub-Saharan Africa | 0.643 (0.246 to 1.043) | 0.002 | 0.597 (0.199 to 0.997) | 0.003 | 0.531 (0.13 to 0.933) | 0.009 |
| Western Sub-Saharan Africa | 0.597 (0.395 to 0.799) | <0.001 | 0.635 (0.43 to 0.841) | <0.001 | 0.367 (0.198 to 0.535) | <0.001 |
| **Socio-demographic index** |  |  |  |  |  |  |
| High SDI | -0.743 (-0.909 to -0.577) | <0.001 | -1.488 (-1.613 to -1.364) | <0.001 | -1.832 (-1.967 to -1.697) | <0.001 |
| High-middle SDI | -0.322 (-0.678 to 0.035) | 0.077 | -1.212 (-1.312 to -1.112) | <0.001 | -1.221 (-1.422 to -1.02) | <0.001 |
| Middle SDI | -0.019 (-0.138 to 0.1) | 0.756 | -0.565 (-0.696 to -0.433) | <0.001 | -0.643 (-0.738 to -0.548) | <0.001 |
| Low-middle SDI | 0.657 (0.498 to 0.816) | <0.001 | 0.6 (0.414 to 0.785) | <0.001 | 0.493 (0.369 to 0.617) | <0.001 |
| Low SDI | 0.75 (0.555 to 0.946) | <0.001 | 0.754 (0.546 to 0.964) | <0.001 | 0.516 (0.351 to 0.682) | <0.001 |
| GBTC, gallbladder and biliary tract cancer; AAPC, average annual percentage change; DALYs, disability-adjusted life years; ASR, age-standardized rate, per 105 Population; rate;UI, Uncertainty interval; SDI, socio-demographic index ; | | | | | | |

| Supplementary Table 2. National ASIR of GBTC in 2021, and its temporal trend from 1990 to 2021 | | | | | |
| --- | --- | --- | --- | --- | --- |
| Nations | 2021 | | | 1990-2021 | |
|  | ASR per 100,000 | Upper 95%UI | Lower95%UI | AAPC (95CI%) | P value |
| Afghanistan | 1.777584107 | 2.777743683 | 0.871825146 | 0.844(0.748 to 0.94) | <0.001 |
| Albania | 1.27703221 | 1.869631976 | 0.866331594 | -0.544(-1.09 to 0.004) | 0.052 |
| Algeria | 3.233488493 | 4.172992223 | 2.338955294 | -0.476(-0.674 to -0.278) | <0.001 |
| American Samoa | 0.498978352 | 0.779905638 | 0.388461008 | -0.873(-1.387 to -0.356) | 0.001 |
| Andorra | 2.853640422 | 3.914090342 | 1.921962177 | -0.905(-1.36 to -0.447) | <0.001 |
| Angola | 0.370595649 | 0.516685783 | 0.241775623 | 0.555(0.396 to 0.713) | <0.001 |
| Antigua and Barbuda | 1.11648861 | 1.181633699 | 1.048570992 | -2.359(-3.53 to -1.173) | <0.001 |
| Argentina | 3.322377204 | 3.535822057 | 3.081093374 | -2.024(-2.317 to -1.73) | <0.001 |
| Armenia | 1.558943691 | 1.836359798 | 1.311355594 | 2.307(0.953 to 3.679) | 0.001 |
| Australia | 3.307770801 | 3.567074879 | 2.922479864 | -0.409(-1.096 to 0.284) | 0.247 |
| Austria | 2.332705302 | 2.534096292 | 2.081415761 | -2.201(-2.598 to -1.802) | <0.001 |
| Azerbaijan | 0.655608288 | 0.983931265 | 0.38799392 | -0.787(-1.366 to -0.205) | 0.008 |
| Bahamas | 1.48534324 | 1.81496942 | 1.2177533 | -1.486(-1.772 to -1.2) | <0.001 |
| Bahrain | 0.991276674 | 1.31441442 | 0.661773318 | -0.47(-0.847 to -0.092) | 0.015 |
| Bangladesh | 1.839283623 | 2.803743254 | 1.240284809 | 0.334(-0.038 to 0.707) | 0.079 |
| Barbados | 1.316624539 | 1.641538737 | 1.028733073 | -0.837(-1.226 to -0.446) | <0.001 |
| Belarus | 1.470471066 | 1.791912806 | 1.198220909 | 0.641(0.128 to 1.158) | 0.014 |
| Belgium | 1.304756786 | 1.416120749 | 1.155250053 | -1.719(-2.021 to -1.416) | <0.001 |
| Belize | 1.01282773 | 1.147803146 | 0.895507939 | -1.614(-2.397 to -0.824) | <0.001 |
| Benin | 0.041703743 | 0.056307637 | 0.021379023 | 0.602(0.427 to 0.777) | <0.001 |
| Bermuda | 0.752998798 | 0.921737248 | 0.637755118 | -2.718(-3.387 to -2.045) | <0.001 |
| Bhutan | 2.12737993 | 3.066604071 | 1.465891197 | 0.475(0.411 to 0.538) | <0.001 |
| Bolivia (Plurinational State of) | 5.479699643 | 7.89283673 | 3.636287009 | -0.912(-0.991 to -0.833) | <0.001 |
| Bosnia and Herzegovina | 3.08490507 | 5.265335604 | 2.242312245 | -1.551(-1.888 to -1.212) | <0.001 |
| Botswana | 0.642232558 | 0.961631805 | 0.455681573 | -0.149(-1.036 to 0.746) | 0.743 |
| Brazil | 2.290141306 | 2.404040436 | 2.108164825 | -1.029(-1.244 to -0.813) | <0.001 |
| Brunei Darussalam | 2.51079227 | 3.222480105 | 1.883379589 | -1.326(-1.521 to -1.132) | <0.001 |
| Bulgaria | 1.654673914 | 1.943082592 | 1.39224001 | -0.35(-1 to 0.305) | 0.295 |
| Burkina Faso | 0.049928949 | 0.067152775 | 0.02449826 | 0.735(0.475 to 0.995) | <0.001 |
| Burundi | 0.726706536 | 1.058697725 | 0.411860059 | -1.009(-1.179 to -0.84) | <0.001 |
| Cabo Verde | 0.349099293 | 0.464521276 | 0.134281816 | 3.877(3.623 to 4.131) | <0.001 |
| Cambodia | 1.035057649 | 2.189440825 | 0.675920026 | -0.059(-0.163 to 0.046) | 0.269 |
| Cameroon | 0.05226085 | 0.073845092 | 0.025945418 | 0.664(0.577 to 0.752) | <0.001 |
| Canada | 1.764037912 | 1.912158501 | 1.570613729 | -1.544(-1.785 to -1.303) | <0.001 |
| Central African Republic | 0.336059787 | 0.550299102 | 0.208508782 | -0.263(-0.424 to -0.102) | 0.001 |
| Chad | 0.043985333 | 0.057929632 | 0.025214753 | 1.284(1.064 to 1.506) | <0.001 |
| Chile | 9.008990587 | 9.774763911 | 8.218560892 | -2.08(-2.461 to -1.698) | <0.001 |
| China | 2.492644784 | 3.212989513 | 1.71186546 | 0.402(0.216 to 0.588) | <0.001 |
| Colombia | 2.499122917 | 2.924261828 | 2.086737061 | -1.755(-1.959 to -1.552) | <0.001 |
| Comoros | 0.783387987 | 1.185690032 | 0.49660661 | 0.05(-0.056 to 0.156) | 0.356 |
| Congo | 0.405017607 | 0.527722497 | 0.267331427 | -0.02(-0.204 to 0.165) | 0.832 |
| Cook Islands | 0.518378222 | 0.802282854 | 0.34921706 | -1.119(-1.624 to -0.61) | <0.001 |
| Costa Rica | 2.029073418 | 2.285058569 | 1.769677855 | -1.981(-2.673 to -1.285) | <0.001 |
| Coted'Ivoire | 0.048878747 | 0.064701071 | 0.023595844 | 0.874(0.573 to 1.176) | <0.001 |
| Croatia | 3.825327995 | 4.344422507 | 3.248099159 | -0.337(-1.191 to 0.524) | 0.442 |
| Cuba | 0.989492984 | 1.115545279 | 0.852928128 | -1.907(-2.246 to -1.567) | <0.001 |
| Cyprus | 2.041761237 | 2.678443288 | 1.501214197 | -1.501(-1.81 to -1.191) | <0.001 |
| Czechia | 4.399287717 | 5.119703725 | 3.722913118 | -1.756(-2.09 to -1.422) | <0.001 |
| Democratic People's Republic of Korea | 1.459123061 | 2.597062035 | 0.980160301 | -0.545(-0.594 to -0.496) | <0.001 |
| Democratic Republic of the Congo | 0.302468337 | 0.464253469 | 0.189752221 | 0.18(0.034 to 0.326) | 0.015 |
| Denmark | 1.921203405 | 2.096239072 | 1.72161943 | 0.217(-0.547 to 0.987) | 0.58 |
| Djibouti | 0.777422149 | 1.250054862 | 0.46901614 | 0.166(0.117 to 0.215) | <0.001 |
| Dominica | 1.617177888 | 2.025701016 | 1.208975637 | -0.843(-0.975 to -0.711) | <0.001 |
| Dominican Republic | 0.806156572 | 1.145089343 | 0.609978918 | -0.81(-1.206 to -0.412) | <0.001 |
| Ecuador | 2.948017146 | 3.631317135 | 2.334236823 | -1.494(-2.301 to -0.681) | <0.001 |
| Egypt | 1.374416384 | 1.733277464 | 0.999225555 | 1.016(0.702 to 1.331) | <0.001 |
| El Salvador | 2.515996601 | 3.46612602 | 1.93964733 | -0.373(-0.61 to -0.136) | 0.002 |
| Equatorial Guinea | 0.376449715 | 0.557808889 | 0.235971082 | 0.459(0.174 to 0.746) | 0.002 |
| Eritrea | 0.980803532 | 1.425631522 | 0.590578717 | 0.268(0.186 to 0.35) | <0.001 |
| Estonia | 1.889522908 | 2.14484755 | 1.632374511 | -0.284(-0.943 to 0.38) | 0.402 |
| Eswatini | 0.922801255 | 1.39230893 | 0.588726471 | 0.975(0.836 to 1.114) | <0.001 |
| Ethiopia | 1.291596145 | 1.816050548 | 0.888552234 | -1.12(-1.235 to -1.005) | <0.001 |
| Fiji | 1.042040472 | 1.420594615 | 0.622376318 | 0.22(-0.105 to 0.547) | 0.184 |
| Finland | 2.577052243 | 2.831115934 | 2.273765255 | -1.048(-1.676 to -0.415) | 0.001 |
| France | 1.481708584 | 1.657133307 | 1.29841887 | -2.16(-2.463 to -1.856) | <0.001 |
| Gabon | 0.411818344 | 0.549023808 | 0.268920972 | -0.073(-0.227 to 0.081) | 0.352 |
| Gambia | 3.63E-05 | 4.77E-05 | 1.71E-05 | 0.231(-0.197 to 0.66) | 0.291 |
| Georgia | 1.498236107 | 1.814046663 | 1.225667128 | -0.669(-1.766 to 0.441) | 0.237 |
| Germany | 3.282343673 | 3.556689246 | 2.932288071 | -1.864(-2.338 to -1.388) | <0.001 |
| Ghana | 0.052572063 | 0.069588417 | 0.02704311 | 1.475(1.33 to 1.62) | <0.001 |
| Greece | 1.90545471 | 2.041769028 | 1.739309419 | -0.771(-1.447 to -0.091) | 0.026 |
| Greenland | 1.729195148 | 2.459141081 | 1.313147322 | -2.431(-2.907 to -1.952) | <0.001 |
| Grenada | 1.361248873 | 1.544799112 | 1.184640096 | -1.14(-1.379 to -0.9) | <0.001 |
| Guam | 0.284251312 | 0.3872852 | 0.217725226 | -1.857(-2.687 to -1.02) | <0.001 |
| Guatemala | 1.596646389 | 1.846014423 | 1.38170563 | -3.554(-4.195 to -2.908) | <0.001 |
| Guinea | 0.043259043 | 0.061067822 | 0.023003522 | 0.909(0.782 to 1.036) | <0.001 |
| Guinea-Bissau | 0.061383865 | 0.087826198 | 0.033679351 | 0.964(0.838 to 1.09) | <0.001 |
| Guyana | 1.058416032 | 1.34146265 | 0.834945587 | -1.189(-1.774 to -0.6) | <0.001 |
| Haiti | 1.459683152 | 2.187594028 | 0.918839634 | -1.015(-1.083 to -0.947) | <0.001 |
| Honduras | 3.199274285 | 4.266977027 | 2.174119837 | 1.091(0.659 to 1.525) | <0.001 |
| Hungary | 3.102802826 | 3.498920157 | 2.701588916 | -2.711(-3.075 to -2.346) | <0.001 |
| Iceland | 1.668332474 | 1.881807291 | 1.449770137 | -1.042(-1.458 to -0.625) | <0.001 |
| India | 2.237553964 | 2.641442802 | 1.537450533 | 1.292(0.951 to 1.634) | <0.001 |
| Indonesia | 0.690602731 | 1.484323038 | 0.463791609 | -0.258(-0.324 to -0.193) | <0.001 |
| Iran (Islamic Republic of) | 0.875087245 | 1.021254715 | 0.473559004 | 1.648(1.359 to 1.939) | <0.001 |
| Iraq | 1.072722379 | 1.369496154 | 0.734876217 | 0.152(0.022 to 0.283) | 0.022 |
| Ireland | 1.230070088 | 1.363622115 | 1.065352939 | -1.481(-2.485 to -0.466) | 0.004 |
| Israel | 1.112228553 | 1.229058731 | 0.980493209 | -1.933(-3.02 to -0.832) | 0.001 |
| Italy | 3.225117681 | 3.494938708 | 2.872040783 | -0.734(-0.869 to -0.6) | <0.001 |
| Jamaica | 1.06525184 | 1.381651628 | 0.832144996 | -0.679(-1.732 to 0.385) | 0.21 |
| Japan | 6.260713818 | 6.790456726 | 5.337646806 | -1.35(-1.546 to -1.153) | <0.001 |
| Jordan | 1.294871241 | 1.800646786 | 0.897580472 | -0.889(-1.48 to -0.296) | 0.003 |
| Kazakhstan | 1.081159603 | 1.270691192 | 0.904814213 | -0.533(-1.164 to 0.102) | 0.1 |
| Kenya | 1.122759663 | 1.613318409 | 0.808344352 | 0.527(0.394 to 0.661) | <0.001 |
| Kiribati | 0.606177432 | 0.818009857 | 0.287331443 | 0.116(0.051 to 0.181) | <0.001 |
| Kuwait | 1.3073353 | 1.544696412 | 1.075927031 | -1.78(-5.553 to 2.144) | 0.369 |
| Kyrgyzstan | 1.026621496 | 1.240889555 | 0.847424971 | 0.047(-1.693 to 1.818) | 0.958 |
| Lao People's Democratic Republic | 0.818657902 | 1.80530074 | 0.524934069 | -0.948(-1.007 to -0.888) | <0.001 |
| Latvia | 1.306897397 | 1.488248383 | 1.131839235 | 0.353(-0.846 to 1.567) | 0.565 |
| Lebanon | 1.560645872 | 2.042687397 | 0.980824415 | -0.551(-0.773 to -0.328) | <0.001 |
| Lesotho | 0.931632749 | 1.387870608 | 0.618881843 | 2.101(1.792 to 2.411) | <0.001 |
| Liberia | 0.042950014 | 0.062042007 | 0.021613766 | 0.547(0.174 to 0.921) | 0.004 |
| Libya | 3.6031555 | 4.928130864 | 2.417929924 | 0.14(-0.092 to 0.372) | 0.236 |
| Lithuania | 2.16013988 | 2.439146887 | 1.889669589 | 0.24(-1.563 to 2.075) | 0.796 |
| Luxembourg | 1.499260498 | 1.682485603 | 1.331233038 | -1.825(-2.138 to -1.511) | <0.001 |
| Madagascar | 0.632245056 | 0.885003363 | 0.384600007 | -0.22(-0.325 to -0.114) | <0.001 |
| Malawi | 0.256019889 | 0.366963744 | 0.157234727 | 0.097(-0.034 to 0.228) | 0.146 |
| Malaysia | 1.128063792 | 1.366536029 | 0.710165357 | -0.314(-0.61 to -0.018) | 0.038 |
| Maldives | 0.589022029 | 0.761249449 | 0.412541294 | -1.912(-2.154 to -1.67) | <0.001 |
| Mali | 0.230189565 | 0.352723604 | 0.164454315 | 0.07(-0.068 to 0.209) | 0.318 |
| Malta | 1.078758134 | 1.210184205 | 0.949997775 | -1.632(-1.738 to -1.525) | <0.001 |
| Marshall Islands | 0.624875095 | 0.859460322 | 0.405676265 | -0.476(-0.566 to -0.386) | <0.001 |
| Mauritania | 0.042400827 | 0.061200357 | 0.022785747 | 0.285(0.037 to 0.534) | 0.024 |
| Mauritius | 0.795069821 | 0.842705306 | 0.733623347 | -2.294(-4.533 to -0.003) | 0.05 |
| Mexico | 2.16706771 | 2.433709411 | 1.909523844 | -2.539(-3.074 to -2.001) | <0.001 |
| Micronesia (Federated States of) | 0.638400357 | 0.866874553 | 0.42093279 | -0.798(-0.867 to -0.729) | <0.001 |
| Monaco | 0.944606726 | 1.274841186 | 0.68484958 | -0.078(-0.201 to 0.044) | 0.211 |
| Mongolia | 2.685645991 | 4.008919179 | 1.945761135 | -0.74(-1.016 to -0.463) | <0.001 |
| Montenegro | 1.642336452 | 2.207636273 | 1.191431911 | 0.272(-0.024 to 0.569) | 0.071 |
| Morocco | 0.668241592 | 0.857269617 | 0.393641158 | 0.191(0.136 to 0.247) | <0.001 |
| Mozambique | 0.892933174 | 1.297948554 | 0.496894992 | 0.732(0.584 to 0.88) | <0.001 |
| Myanmar | 0.689621392 | 1.444831331 | 0.453367502 | -1.024(-1.101 to -0.947) | <0.001 |
| Namibia | 0.533370555 | 0.755389124 | 0.357149261 | 0.242(0.023 to 0.462) | 0.03 |
| Nauru | 0.855145532 | 1.199744621 | 0.508761829 | -0.5(-0.57 to -0.43) | <0.001 |
| Nepal | 2.146804038 | 2.990383671 | 1.572737834 | 0.833(0.758 to 0.909) | <0.001 |
| Netherlands | 2.465010488 | 2.700704958 | 2.20161187 | -0.936(-1.305 to -0.566) | <0.001 |
| New Zealand | 3.593410396 | 3.93566947 | 3.190941642 | 1.433(-0.558 to 3.464) | 0.159 |
| Nicaragua | 2.101076334 | 3.261729326 | 1.639126675 | -1.288(-1.758 to -0.815) | <0.001 |
| Niger | 0.038358264 | 0.054754587 | 0.020896912 | 0.658(0.427 to 0.89) | <0.001 |
| Nigeria | 0.041779203 | 0.057181137 | 0.029023176 | 0.424(0.264 to 0.584) | <0.001 |
| Niue | 0.610994877 | 0.779740656 | 0.42391228 | -0.48(-0.563 to -0.397) | <0.001 |
| North Macedonia | 2.448271478 | 3.130882856 | 1.79645129 | -0.378(-0.549 to -0.206) | <0.001 |
| Northern Mariana Islands | 0.408332849 | 0.71847927 | 0.323667711 | -0.263(-0.766 to 0.244) | 0.309 |
| Norway | 1.985596452 | 2.138265672 | 1.774968207 | 0.399(-0.401 to 1.207) | 0.329 |
| Oman | 0.747424214 | 0.993248564 | 0.532073408 | -0.631(-1.043 to -0.218) | 0.003 |
| Pakistan | 3.223499027 | 4.346396205 | 2.353068763 | 0.49(0.449 to 0.53) | <0.001 |
| Palau | 0.414590684 | 0.58220633 | 0.28259958 | -0.285(-0.412 to -0.158) | <0.001 |
| Palestine | 1.337865586 | 1.646908159 | 0.847658048 | -0.648(-0.804 to -0.493) | <0.001 |
| Panama | 1.22900717 | 1.466202028 | 0.981669382 | -2.341(-3.153 to -1.522) | <0.001 |
| Papua New Guinea | 0.441796946 | 0.622693226 | 0.288642854 | -0.788(-0.877 to -0.699) | <0.001 |
| Paraguay | 1.874225831 | 2.626442507 | 1.306907007 | 0.128(-0.109 to 0.366) | 0.29 |
| Peru | 4.129654348 | 5.706092007 | 2.895655113 | -0.175(-0.98 to 0.636) | 0.671 |
| Philippines | 0.507217069 | 0.865810962 | 0.405477451 | 0.324(0.291 to 0.357) | <0.001 |
| Poland | 2.800534494 | 3.033887307 | 2.545504525 | -1.798(-2.147 to -1.447) | <0.001 |
| Portugal | 2.246280278 | 2.447217 | 2.019115764 | -0.687(-1.323 to -0.048) | 0.035 |
| Puerto Rico | 0.766305736 | 0.898970948 | 0.627001225 | -1.946(-2.575 to -1.313) | <0.001 |
| Qatar | 1.680537251 | 2.384630889 | 1.179134044 | -0.892(-1.764 to -0.012) | 0.047 |
| Republic of Korea | 8.1249583 | 10.30747354 | 5.056211698 | -0.874(-1.144 to -0.603) | <0.001 |
| Republic of Moldova | 0.875206579 | 0.978013718 | 0.790897841 | -1.806(-3.255 to -0.336) | 0.016 |
| Romania | 1.566897532 | 1.769610626 | 1.369833502 | -1.304(-2.305 to -0.294) | 0.012 |
| Russian Federation | 2.129111635 | 2.297270345 | 1.956484956 | 0.666(-0.455 to 1.799) | 0.246 |
| Rwanda | 0.9100223 | 1.274330732 | 0.57943518 | -0.752(-0.847 to -0.656) | <0.001 |
| Saint Kitts and Nevis | 0.98882745 | 1.156178054 | 0.825549844 | -2.355(-2.868 to -1.839) | <0.001 |
| Saint Lucia | 0.812644936 | 0.981010569 | 0.663672075 | -2.388(-2.77 to -2.005) | <0.001 |
| Saint Vincent and the Grenadines | 0.862140581 | 0.978514524 | 0.763556831 | -2.192(-2.697 to -1.684) | <0.001 |
| Samoa | 0.611681276 | 0.795829656 | 0.421856227 | -0.448(-0.482 to -0.413) | <0.001 |
| San Marino | 2.037387983 | 2.982671046 | 1.26137647 | -2.35(-2.515 to -2.185) | <0.001 |
| Sao Tome and Principe | 1.3934075 | 1.807508659 | 0.925235548 | 0.576(0.414 to 0.739) | <0.001 |
| Saudi Arabia | 1.717699573 | 2.548083588 | 1.293865768 | 0.232(0.051 to 0.414) | 0.012 |
| Senegal | 0.045332234 | 0.06519951 | 0.024592771 | 0.916(0.744 to 1.088) | <0.001 |
| Serbia | 2.166603244 | 2.878261196 | 1.513210994 | -0.84(-1.044 to -0.636) | <0.001 |
| Seychelles | 0.883009685 | 1.301406676 | 0.713575419 | -1.867(-2.097 to -1.637) | <0.001 |
| Sierra Leone | 0.042960305 | 0.058760475 | 0.021628497 | 0.955(0.782 to 1.128) | <0.001 |
| Singapore | 1.328937694 | 1.459762053 | 1.17697523 | -1.07(-1.39 to -0.749) | <0.001 |
| Slovakia | 5.683271251 | 7.707252272 | 4.066737184 | -0.518(-0.627 to -0.409) | <0.001 |
| Slovenia | 3.536037193 | 4.100535944 | 2.961532053 | -0.781(-1.413 to -0.145) | 0.016 |
| Solomon Islands | 0.57388236 | 0.825657469 | 0.374165322 | -0.361(-0.511 to -0.21) | <0.001 |
| Somalia | 0.77537873 | 1.290452145 | 0.457701386 | 0.138(0.049 to 0.228) | 0.002 |
| South Africa | 0.802678096 | 0.94704361 | 0.530490942 | 0.725(0.175 to 1.279) | 0.01 |
| South Sudan | 0.762594717 | 1.11587147 | 0.476656568 | 0.351(0.28 to 0.422) | <0.001 |
| Spain | 2.895735501 | 3.190977248 | 2.554870164 | -0.847(-1.408 to -0.284) | 0.003 |
| Sri Lanka | 0.80808067 | 1.496065832 | 0.494553439 | -3.431(-4.326 to -2.529) | <0.001 |
| Sudan | 0.883717038 | 1.279806978 | 0.612941395 | 0.212(0.146 to 0.279) | <0.001 |
| Suriname | 0.891819013 | 1.207930309 | 0.647692202 | -1.14(-1.52 to -0.76) | <0.001 |
| Sweden | 2.623306 | 2.949800115 | 2.278793308 | -2.187(-2.73 to -1.642) | <0.001 |
| Switzerland | 2.53830593 | 2.906542824 | 2.169349899 | 0.293(-0.418 to 1.008) | 0.42 |
| Syrian Arab Republic | 0.087390672 | 0.116557916 | 0.04973968 | 0.535(0.278 to 0.792) | <0.001 |
| Taiwan (Province of China) | 3.047529188 | 3.32992726 | 2.732274782 | 0.237(-0.199 to 0.675) | 0.288 |
| Tajikistan | 0.10979568 | 0.152242544 | 0.072541415 | -1.632(-2.228 to -1.032) | <0.001 |
| Thailand | 7.10887958 | 9.691714049 | 3.599216428 | 0.352(0.123 to 0.583) | 0.003 |
| Timor-Leste | 0.62888632 | 1.328058001 | 0.414629792 | -0.611(-0.845 to -0.377) | <0.001 |
| Togo | 0.050534045 | 0.073806679 | 0.025380734 | 1.151(0.985 to 1.317) | <0.001 |
| Tokelau | 0.553735198 | 0.74623472 | 0.382388325 | -0.929(-0.983 to -0.875) | <0.001 |
| Tonga | 0.464018358 | 0.656138733 | 0.317706881 | -0.433(-0.59 to -0.276) | <0.001 |
| Trinidad and Tobago | 1.0002788 | 1.273269195 | 0.773077036 | -2.135(-2.484 to -1.785) | <0.001 |
| Tunisia | 2.2653344 | 3.101093415 | 1.493975354 | -0.026(-0.108 to 0.056) | 0.526 |
| Turkey | 1.456838197 | 1.934080084 | 1.080578023 | -1.082(-1.278 to -0.886) | <0.001 |
| Turkmenistan | 0.749228172 | 0.97533502 | 0.575065925 | -3.377(-5.997 to -0.683) | 0.014 |
| Tuvalu | 0.582391335 | 0.747517364 | 0.401205494 | -0.92(-0.973 to -0.866) | <0.001 |
| Uganda | 0.764913896 | 1.068982617 | 0.534422706 | 0.235(0.135 to 0.335) | <0.001 |
| Ukraine | 1.121353016 | 1.445175271 | 0.833674414 | -0.051(-0.69 to 0.591) | 0.875 |
| United Arab Emirates | 3.953477194 | 5.518270888 | 2.94095025 | -0.839(-2.74 to 1.099) | 0.394 |
| United Kingdom | 2.517958113 | 2.633035727 | 2.30830165 | 0.879(0.576 to 1.184) | <0.001 |
| United Republic of Tanzania | 0.791706093 | 1.084108018 | 0.515239375 | -0.013(-0.096 to 0.07) | 0.76 |
| United States of America | 2.186689828 | 2.298009237 | 2.007186229 | -0.418(-0.723 to -0.113) | 0.007 |
| United States Virgin Islands | 0.933096499 | 1.303157338 | 0.660034603 | -2.645(-3.038 to -2.25) | <0.001 |
| Uruguay | 4.243120495 | 4.603185791 | 3.86542821 | -1.052(-1.416 to -0.686) | <0.001 |
| Uzbekistan | 0.365354036 | 0.465474476 | 0.283018365 | 1.309(0.974 to 1.645) | <0.001 |
| Vanuatu | 0.513025618 | 0.697633197 | 0.347730248 | -0.5(-0.655 to -0.344) | <0.001 |
| Venezuela (Bolivarian Republic of) | 1.609870563 | 2.077599812 | 1.202817736 | -2.809(-3.307 to -2.309) | <0.001 |
| Viet Nam | 1.272163403 | 1.869910433 | 0.87125019 | 0.721(0.659 to 0.783) | <0.001 |
| Yemen | 0.99283341 | 1.490102375 | 0.645045117 | 0.41(0.272 to 0.548) | <0.001 |
| Zambia | 0.903612304 | 1.216109911 | 0.560424275 | 0.289(0.126 to 0.454) | 0.001 |
| Zimbabwe | 1.166003058 | 1.54840583 | 0.819461467 | 0.507(0.126 to 0.889) | 0.009 |
| GBTC, gallbladder and biliary tract cancer; ASIR, age-standardized incidence rate; AAPC, average annual percentage change;UI, Uncertainty interval; | | | | | |

| Supplementary Table 3. National ASMR of GBTC in 2021, and its temporal trend from 1990 to 2021 | | | | | |
| --- | --- | --- | --- | --- | --- |
| Nations | 2021 | | | 1990-2021 | |
|  | ASR per 100,000 | Upper 95%UI | Lower95%UI | AAPC (95CI%) | P value |
| Afghanistan | 1.899658392 | 2.919511417 | 0.963715503 | 0.856(0.776 to 0.935) | <0.001 |
| Albania | 1.300503222 | 1.891747664 | 0.873301164 | -0.763(-1.174 to -0.35) | <0.001 |
| Algeria | 3.281733163 | 4.269674179 | 2.371654749 | -0.73(-0.956 to -0.504) | <0.001 |
| American Samoa | 0.515091994 | 0.79039115 | 0.40288197 | -0.977(-1.496 to -0.456) | <0.001 |
| Andorra | 1.746685229 | 2.40197216 | 1.188042041 | -1.737(-2.162 to -1.312) | <0.001 |
| Angola | 0.403390473 | 0.566377985 | 0.265273701 | 0.548(0.399 to 0.698) | <0.001 |
| Antigua and Barbuda | 1.11724155 | 1.18827828 | 1.049358058 | -2.47(-3.645 to -1.281) | <0.001 |
| Argentina | 3.161310165 | 3.370947722 | 2.91528833 | -2.319(-2.77 to -1.867) | <0.001 |
| Armenia | 1.589046643 | 1.865899609 | 1.331833161 | 2.212(0.844 to 3.598) | 0.001 |
| Australia | 0.998523438 | 1.076701635 | 0.882721777 | -1.929(-2.588 to -1.266) | <0.001 |
| Austria | 1.652990937 | 1.791439726 | 1.472352854 | -3.145(-3.562 to -2.726) | <0.001 |
| Azerbaijan | 0.686062174 | 1.0363531 | 0.404369504 | -0.822(-1.408 to -0.232) | 0.006 |
| Bahamas | 1.502382153 | 1.82856095 | 1.237289773 | -1.578(-1.872 to -1.283) | <0.001 |
| Bahrain | 0.921495007 | 1.204947224 | 0.616873191 | -0.9(-1.308 to -0.49) | <0.001 |
| Bangladesh | 1.940551396 | 2.952860923 | 1.324012564 | 0.268(-0.13 to 0.666) | 0.187 |
| Barbados | 1.312189479 | 1.617961251 | 1.026195402 | -0.957(-1.359 to -0.553) | <0.001 |
| Belarus | 1.340931372 | 1.627945653 | 1.092896856 | 0.097(-0.406 to 0.603) | 0.707 |
| Belgium | 0.856406514 | 0.93874638 | 0.751428222 | -2.542(-2.818 to -2.264) | <0.001 |
| Belize | 1.041210026 | 1.174960968 | 0.918844178 | -1.696(-2.517 to -0.869) | <0.001 |
| Benin | 0.047141402 | 0.063904238 | 0.023816004 | 0.717(0.555 to 0.879) | <0.001 |
| Bermuda | 0.582472352 | 0.71329788 | 0.491654444 | -3.571(-4.209 to -2.928) | <0.001 |
| Bhutan | 2.270143607 | 3.224912489 | 1.580102095 | 0.427(0.364 to 0.489) | <0.001 |
| Bolivia (Plurinational State of) | 5.902940451 | 8.3741915 | 3.91284556 | -0.948(-1.046 to -0.849) | <0.001 |
| Bosnia and Herzegovina | 3.067461115 | 5.155183902 | 2.234462911 | -1.7(-1.98 to -1.42) | <0.001 |
| Botswana | 0.699584182 | 1.051276075 | 0.495593055 | -0.15(-1.017 to 0.725) | 0.736 |
| Brazil | 2.31931518 | 2.443631553 | 2.12052199 | -1.216(-1.417 to -1.014) | <0.001 |
| Brunei Darussalam | 2.64994827 | 3.372248125 | 1.948779781 | -1.458(-1.663 to -1.252) | <0.001 |
| Bulgaria | 1.327786569 | 1.559382901 | 1.118959269 | -0.846(-1.317 to -0.372) | <0.001 |
| Burkina Faso | 0.056694441 | 0.077401189 | 0.027721714 | 0.867(0.6 to 1.136) | <0.001 |
| Burundi | 0.798240976 | 1.159039284 | 0.457201088 | -0.977(-1.154 to -0.8) | <0.001 |
| Cabo Verde | 0.373179392 | 0.497401345 | 0.137797717 | 3.811(3.555 to 4.067) | <0.001 |
| Cambodia | 1.091098807 | 2.319585139 | 0.719042908 | -0.127(-0.232 to -0.022) | 0.017 |
| Cameroon | 0.058707846 | 0.083433685 | 0.028104396 | 0.765(0.676 to 0.854) | <0.001 |
| Canada | 0.963792728 | 1.042473072 | 0.849492999 | -2.35(-2.771 to -1.928) | <0.001 |
| Central African Republic | 0.36875793 | 0.601831387 | 0.226562439 | -0.24(-0.395 to -0.084) | 0.003 |
| Chad | 0.049555618 | 0.066084161 | 0.027719126 | 1.375(1.165 to 1.586) | <0.001 |
| Chile | 7.572794598 | 8.208248952 | 6.855989949 | -2.695(-3.068 to -2.321) | <0.001 |
| China | 1.850547051 | 2.396253668 | 1.294636275 | -0.731(-0.942 to -0.52) | <0.001 |
| Colombia | 2.404283285 | 2.811393014 | 2.001165842 | -2.098(-2.439 to -1.754) | <0.001 |
| Comoros | 0.85423117 | 1.293700144 | 0.537920809 | 0.062(-0.033 to 0.158) | 0.198 |
| Congo | 0.438760446 | 0.568445673 | 0.289890806 | -0.038(-0.213 to 0.137) | 0.671 |
| Cook Islands | 0.47134337 | 0.714165967 | 0.319199686 | -1.667(-2.203 to -1.129) | <0.001 |
| Costa Rica | 1.875077769 | 2.104341191 | 1.64140402 | -2.255(-2.923 to -1.583) | <0.001 |
| Coted'Ivoire | 0.054986243 | 0.072476677 | 0.025904774 | 0.986(0.699 to 1.274) | <0.001 |
| Croatia | 2.648769315 | 3.045373088 | 2.267121386 | -1.288(-1.916 to -0.656) | <0.001 |
| Cuba | 0.907837906 | 1.021132447 | 0.783847025 | -2.213(-2.782 to -1.641) | <0.001 |
| Cyprus | 1.40004062 | 1.831080693 | 1.039294116 | -2.568(-3.104 to -2.028) | <0.001 |
| Czechia | 3.575450137 | 4.161637904 | 3.03692707 | -2.454(-2.759 to -2.147) | <0.001 |
| Democratic People's Republic of Korea | 1.435052369 | 2.571590462 | 0.969927367 | -0.766(-0.802 to -0.73) | <0.001 |
| Democratic Republic of the Congo | 0.330338932 | 0.513361299 | 0.204305837 | 0.18(0.04 to 0.32) | 0.012 |
| Denmark | 1.010737707 | 1.098516085 | 0.910564606 | -1.088(-1.489 to -0.685) | <0.001 |
| Djibouti | 0.849527912 | 1.350054714 | 0.510572359 | 0.16(0.116 to 0.205) | <0.001 |
| Dominica | 1.714242248 | 2.146090439 | 1.27551827 | -0.888(-1.018 to -0.757) | <0.001 |
| Dominican Republic | 0.841218284 | 1.209360555 | 0.634537898 | -0.95(-1.35 to -0.549) | <0.001 |
| Ecuador | 3.063125082 | 3.766172401 | 2.447890157 | -1.623(-2.393 to -0.848) | <0.001 |
| Egypt | 1.416432627 | 1.762644573 | 1.038095604 | 0.894(0.509 to 1.28) | <0.001 |
| El Salvador | 2.50840485 | 3.474554698 | 1.918529973 | -0.58(-0.833 to -0.326) | <0.001 |
| Equatorial Guinea | 0.402071888 | 0.599713644 | 0.252818832 | 0.395(0.123 to 0.668) | 0.004 |
| Eritrea | 1.078924422 | 1.55771766 | 0.645811656 | 0.304(0.228 to 0.38) | <0.001 |
| Estonia | 1.661093733 | 1.888453793 | 1.431718844 | -0.699(-1.182 to -0.213) | 0.005 |
| Eswatini | 0.997412544 | 1.482741864 | 0.636538362 | 0.939(0.806 to 1.071) | <0.001 |
| Ethiopia | 1.4144634 | 1.981776695 | 0.972690331 | -1.111(-1.227 to -0.994) | <0.001 |
| Fiji | 1.109738255 | 1.486613216 | 0.658874742 | 0.216(-0.11 to 0.542) | 0.195 |
| Finland | 1.794827284 | 1.964300103 | 1.577449666 | -1.855(-2.342 to -1.365) | <0.001 |
| France | 0.953461589 | 1.061085797 | 0.834387087 | -3.12(-3.443 to -2.796) | <0.001 |
| Gabon | 0.444693287 | 0.592778924 | 0.288561884 | -0.108(-0.268 to 0.051) | 0.181 |
| Gambia | 3.96E-05 | 5.27E-05 | 1.87E-05 | 0.208(-0.191 to 0.609) | 0.307 |
| Georgia | 1.549340209 | 1.872064539 | 1.260722519 | -0.768(-2.002 to 0.482) | 0.227 |
| Germany | 2.009837772 | 2.167837306 | 1.779160337 | -2.976(-3.461 to -2.489) | <0.001 |
| Ghana | 0.058942459 | 0.078429216 | 0.029922488 | 1.582(1.438 to 1.726) | <0.001 |
| Greece | 1.332918502 | 1.418460509 | 1.215057156 | -1.116(-2.038 to -0.185) | 0.019 |
| Greenland | 1.662063701 | 2.379456487 | 1.253060947 | -2.703(-3.203 to -2.201) | <0.001 |
| Grenada | 1.401278571 | 1.59569928 | 1.223726814 | -1.069(-1.522 to -0.613) | <0.001 |
| Guam | 0.261479249 | 0.357892396 | 0.199551181 | -2.25(-3.138 to -1.353) | <0.001 |
| Guatemala | 1.695280084 | 1.946189645 | 1.470895972 | -3.669(-4.31 to -3.023) | <0.001 |
| Guinea | 0.048797804 | 0.069644144 | 0.02511958 | 1.016(0.887 to 1.146) | <0.001 |
| Guinea-Bissau | 0.069207827 | 0.099655259 | 0.037100115 | 1.091(0.924 to 1.258) | <0.001 |
| Guyana | 1.114479935 | 1.407115503 | 0.878296169 | -1.258(-1.828 to -0.685) | <0.001 |
| Haiti | 1.586288453 | 2.380241092 | 1.005978478 | -1.018(-1.083 to -0.953) | <0.001 |
| Honduras | 3.419118894 | 4.542859784 | 2.327753508 | 1.049(0.644 to 1.455) | <0.001 |
| Hungary | 2.907364259 | 3.266800518 | 2.535934366 | -3.069(-3.464 to -2.671) | <0.001 |
| Iceland | 0.934828402 | 1.045493511 | 0.805049614 | -1.972(-2.205 to -1.739) | <0.001 |
| India | 2.349614872 | 2.771034121 | 1.607266124 | 1.274(0.868 to 1.682) | <0.001 |
| Indonesia | 0.726651022 | 1.579169123 | 0.481586087 | -0.312(-0.368 to -0.256) | <0.001 |
| Iran (Islamic Republic of) | 0.820728255 | 0.952498845 | 0.443749084 | 1.301(0.956 to 1.647) | <0.001 |
| Iraq | 1.058316209 | 1.341575591 | 0.730529157 | -0.024(-0.162 to 0.114) | 0.735 |
| Ireland | 0.746967841 | 0.82300439 | 0.648873016 | -2.625(-3.379 to -1.865) | <0.001 |
| Israel | 0.814036812 | 0.897272663 | 0.716374127 | -2.704(-3.817 to -1.578) | <0.001 |
| Italy | 2.434051994 | 2.629500251 | 2.15833749 | -1.321(-1.707 to -0.933) | <0.001 |
| Jamaica | 1.069730026 | 1.36675562 | 0.834401991 | -0.785(-1.789 to 0.229) | 0.129 |
| Japan | 4.570630123 | 4.958437492 | 3.898967177 | -1.882(-2.067 to -1.696) | <0.001 |
| Jordan | 1.2156723 | 1.685533231 | 0.838193526 | -1.27(-1.881 to -0.656) | <0.001 |
| Kazakhstan | 1.107430324 | 1.301237855 | 0.926142994 | -0.555(-1.25 to 0.144) | 0.119 |
| Kenya | 1.228329903 | 1.765952149 | 0.878297392 | 0.548(0.419 to 0.677) | <0.001 |
| Kiribati | 0.659928817 | 0.894730603 | 0.309428948 | 0.128(0.066 to 0.19) | <0.001 |
| Kuwait | 1.037970812 | 1.235761947 | 0.849149716 | -1.838(-4.302 to 0.69) | 0.153 |
| Kyrgyzstan | 1.066377329 | 1.291413562 | 0.873890487 | -0.072(-1.791 to 1.676) | 0.935 |
| Lao People's Democratic Republic | 0.875619004 | 1.936632546 | 0.556277736 | -0.967(-1.029 to -0.905) | <0.001 |
| Latvia | 1.237898467 | 1.415750212 | 1.073884574 | 0.039(-0.93 to 1.017) | 0.938 |
| Lebanon | 1.39965864 | 1.830802183 | 0.869757992 | -1.029(-1.224 to -0.834) | <0.001 |
| Lesotho | 1.014592071 | 1.507302574 | 0.675539093 | 2.095(1.763 to 2.429) | <0.001 |
| Liberia | 0.047890334 | 0.069217516 | 0.023291374 | 0.618(0.303 to 0.934) | <0.001 |
| Libya | 3.581770623 | 4.902226721 | 2.387595112 | -0.045(-0.372 to 0.282) | 0.787 |
| Lithuania | 1.829030212 | 2.063223851 | 1.59320922 | -0.131(-2.001 to 1.775) | 0.892 |
| Luxembourg | 0.977318839 | 1.089314494 | 0.86316119 | -2.761(-3.206 to -2.314) | <0.001 |
| Madagascar | 0.688034982 | 0.956905635 | 0.418885197 | -0.215(-0.318 to -0.112) | <0.001 |
| Malawi | 0.281097529 | 0.397534512 | 0.172448959 | 0.108(-0.061 to 0.278) | 0.211 |
| Malaysia | 1.107103949 | 1.338158103 | 0.702615671 | -0.567(-0.872 to -0.262) | <0.001 |
| Maldives | 0.570501439 | 0.732584916 | 0.392504809 | -2.252(-2.48 to -2.023) | <0.001 |
| Mali | 0.251346162 | 0.385585063 | 0.177837361 | 0.066(-0.068 to 0.2) | 0.334 |
| Malta | 0.729450887 | 0.81142362 | 0.64071672 | -2.53(-2.641 to -2.42) | <0.001 |
| Marshall Islands | 0.667736333 | 0.914215061 | 0.432643028 | -0.519(-0.609 to -0.429) | <0.001 |
| Mauritania | 0.046849389 | 0.067537851 | 0.024262562 | 0.333(0.085 to 0.581) | 0.008 |
| Mauritius | 0.77684181 | 0.824076773 | 0.719803022 | -2.853(-5.846 to 0.235) | 0.07 |
| Mexico | 2.196976986 | 2.462078401 | 1.943796189 | -2.818(-3.425 to -2.207) | <0.001 |
| Micronesia (Federated States of) | 0.679258561 | 0.919613218 | 0.447200052 | -0.855(-0.921 to -0.789) | <0.001 |
| Monaco | 0.592440583 | 0.800653391 | 0.438988221 | -0.627(-0.712 to -0.543) | <0.001 |
| Mongolia | 2.847293521 | 4.270003362 | 2.059003601 | -0.728(-1 to -0.454) | <0.001 |
| Montenegro | 1.572209437 | 2.100224256 | 1.132220776 | 0.444(0.129 to 0.76) | 0.006 |
| Morocco | 0.694242341 | 0.889823976 | 0.408480566 | 0.082(0.008 to 0.155) | 0.03 |
| Mozambique | 0.981317731 | 1.429954371 | 0.558527707 | 0.732(0.583 to 0.882) | <0.001 |
| Myanmar | 0.72911532 | 1.519886179 | 0.480759175 | -1.072(-1.128 to -1.017) | <0.001 |
| Namibia | 0.574187621 | 0.809718985 | 0.383371494 | 0.206(-0.012 to 0.424) | 0.064 |
| Nauru | 0.902184361 | 1.264441571 | 0.53917959 | -0.543(-0.619 to -0.467) | <0.001 |
| Nepal | 2.301326813 | 3.15603484 | 1.690678518 | 0.787(0.706 to 0.868) | <0.001 |
| Netherlands | 1.391824765 | 1.519915892 | 1.239492565 | -2.072(-2.354 to -1.789) | <0.001 |
| New Zealand | 1.612572041 | 1.749509107 | 1.433459845 | -0.039(-1.958 to 1.917) | 0.968 |
| Nicaragua | 2.136146029 | 3.31389615 | 1.671097302 | -1.437(-1.897 to -0.976) | <0.001 |
| Niger | 0.043449972 | 0.063152661 | 0.022856071 | 0.756(0.534 to 0.979) | <0.001 |
| Nigeria | 0.045440994 | 0.063086593 | 0.03256113 | 0.402(0.189 to 0.615) | <0.001 |
| Niue | 0.6277882 | 0.789675533 | 0.439512238 | -0.613(-0.696 to -0.53) | <0.001 |
| North Macedonia | 2.519824131 | 3.176366607 | 1.850184384 | -0.453(-0.593 to -0.314) | <0.001 |
| Northern Mariana Islands | 0.401785862 | 0.702581946 | 0.318886463 | -0.43(-0.986 to 0.129) | 0.132 |
| Norway | 0.946365461 | 1.012594303 | 0.841716214 | -1.353(-2.042 to -0.659) | <0.001 |
| Oman | 0.680668074 | 0.891815657 | 0.48030314 | -1.128(-1.653 to -0.6) | <0.001 |
| Pakistan | 3.486623928 | 4.679343949 | 2.556067161 | 0.486(0.403 to 0.569) | <0.001 |
| Palau | 0.422793405 | 0.600022343 | 0.288141048 | -0.396(-0.52 to -0.272) | <0.001 |
| Palestine | 1.344818133 | 1.657604315 | 0.849686674 | -0.87(-1.035 to -0.704) | <0.001 |
| Panama | 1.1950693 | 1.433995476 | 0.948297394 | -2.568(-3.352 to -1.777) | <0.001 |
| Papua New Guinea | 0.473594709 | 0.67434404 | 0.314362025 | -0.79(-0.873 to -0.708) | <0.001 |
| Paraguay | 1.936189475 | 2.673170919 | 1.356113202 | -0.136(-0.472 to 0.201) | 0.43 |
| Peru | 4.03234692 | 5.616048742 | 2.813732495 | -0.497(-1.302 to 0.315) | 0.23 |
| Philippines | 0.524739226 | 0.906602265 | 0.416566185 | 0.268(0.231 to 0.305) | <0.001 |
| Poland | 3.010697128 | 3.259555564 | 2.733710476 | -1.909(-2.245 to -1.571) | <0.001 |
| Portugal | 1.376638914 | 1.493575105 | 1.220361308 | -1.958(-2.23 to -1.685) | <0.001 |
| Puerto Rico | 0.64178678 | 0.757056716 | 0.525961072 | -2.57(-3.412 to -1.722) | <0.001 |
| Qatar | 1.391931118 | 1.958650232 | 0.977109168 | -1.668(-2.647 to -0.679) | 0.001 |
| Republic of Korea | 5.967525509 | 7.551187429 | 3.725748824 | -2.03(-2.245 to -1.815) | <0.001 |
| Republic of Moldova | 0.857870289 | 0.958746279 | 0.774487998 | -1.969(-3.432 to -0.484) | 0.01 |
| Romania | 1.528630678 | 1.731180398 | 1.336487601 | -1.484(-2.444 to -0.514) | 0.003 |
| Russian Federation | 1.420490923 | 1.535353811 | 1.306602112 | -0.513(-1.446 to 0.43) | 0.285 |
| Rwanda | 0.997141795 | 1.387850062 | 0.642879238 | -0.731(-0.822 to -0.64) | <0.001 |
| Saint Kitts and Nevis | 1.033200005 | 1.192039025 | 0.864359591 | -2.475(-3.003 to -1.943) | <0.001 |
| Saint Lucia | 0.833015721 | 1.000928054 | 0.68196443 | -2.488(-2.868 to -2.106) | <0.001 |
| Saint Vincent and the Grenadines | 0.898507599 | 1.012533467 | 0.796948115 | -2.259(-2.753 to -1.763) | <0.001 |
| Samoa | 0.636639606 | 0.834422104 | 0.436885627 | -0.559(-0.585 to -0.532) | <0.001 |
| San Marino | 1.263101122 | 1.862216776 | 0.772876402 | -2.984(-3.377 to -2.589) | <0.001 |
| Sao Tome and Principe | 1.487838266 | 1.936790913 | 0.991164092 | 0.512(0.349 to 0.674) | <0.001 |
| Saudi Arabia | 1.598459618 | 2.355320232 | 1.205914282 | -0.241(-0.324 to -0.158) | <0.001 |
| Senegal | 0.050909391 | 0.073752029 | 0.026825772 | 1.017(0.819 to 1.215) | <0.001 |
| Serbia | 2.182410894 | 2.909185557 | 1.544570241 | -1.137(-1.333 to -0.941) | <0.001 |
| Seychelles | 0.891807065 | 1.296271865 | 0.729584518 | -1.948(-2.551 to -1.342) | <0.001 |
| Sierra Leone | 0.048246653 | 0.06670268 | 0.023952304 | 1.04(0.849 to 1.232) | <0.001 |
| Singapore | 0.998389205 | 1.088622019 | 0.884129194 | -2.005(-2.358 to -1.651) | <0.001 |
| Slovakia | 3.727178776 | 4.994371047 | 2.705005829 | -1.294(-1.655 to -0.933) | <0.001 |
| Slovenia | 2.311188918 | 2.67032107 | 1.963596779 | -1.913(-2.58 to -1.241) | <0.001 |
| Solomon Islands | 0.613050803 | 0.877631395 | 0.397732697 | -0.411(-0.56 to -0.261) | <0.001 |
| Somalia | 0.847711635 | 1.411005063 | 0.499026537 | 0.157(0.07 to 0.243) | <0.001 |
| South Africa | 0.84872697 | 0.996879087 | 0.564021762 | 0.679(0.141 to 1.221) | 0.013 |
| South Sudan | 0.828508407 | 1.210156557 | 0.514828986 | 0.331(0.264 to 0.399) | <0.001 |
| Spain | 1.334142248 | 1.46593821 | 1.153643943 | -2.281(-2.565 to -1.996) | <0.001 |
| Sri Lanka | 0.753570424 | 1.346246107 | 0.46721988 | -3.873(-4.771 to -2.966) | <0.001 |
| Sudan | 0.927114629 | 1.338570576 | 0.647159916 | 0.143(0.083 to 0.202) | <0.001 |
| Suriname | 0.935157138 | 1.269397911 | 0.682770225 | -1.2(-1.568 to -0.832) | <0.001 |
| Sweden | 2.50122723 | 2.781626928 | 2.17504974 | -1.771(-2.374 to -1.164) | <0.001 |
| Switzerland | 1.0163463 | 1.150049521 | 0.872390956 | -0.901(-1.467 to -0.333) | 0.002 |
| Syrian Arab Republic | 0.085354378 | 0.113317653 | 0.048028158 | 0.267(0.098 to 0.437) | 0.002 |
| Taiwan (Province of China) | 2.152509086 | 2.354983338 | 1.92996837 | -0.579(-0.995 to -0.162) | 0.007 |
| Tajikistan | 0.117658957 | 0.161718011 | 0.077463897 | -1.551(-2.01 to -1.09) | <0.001 |
| Thailand | 6.371251193 | 8.644636182 | 3.19121549 | -0.155(-0.413 to 0.104) | 0.242 |
| Timor-Leste | 0.671578557 | 1.434701739 | 0.439390347 | -0.654(-0.873 to -0.434) | <0.001 |
| Togo | 0.056930339 | 0.083988777 | 0.027602881 | 1.296(1.076 to 1.516) | <0.001 |
| Tokelau | 0.569726274 | 0.768777572 | 0.397560393 | -1.068(-1.112 to -1.024) | <0.001 |
| Tonga | 0.480674115 | 0.681743834 | 0.331540193 | -0.496(-0.652 to -0.339) | <0.001 |
| Trinidad and Tobago | 1.012617416 | 1.283487988 | 0.790571739 | -2.303(-2.65 to -1.955) | <0.001 |
| Tunisia | 2.134337937 | 2.908230053 | 1.433274757 | -0.4(-0.53 to -0.269) | <0.001 |
| Turkey | 1.360576705 | 1.826578721 | 1.017447488 | -1.496(-1.83 to -1.16) | <0.001 |
| Turkmenistan | 0.780982004 | 1.015930466 | 0.603932656 | -3.422(-6.075 to -0.695) | 0.014 |
| Tuvalu | 0.617197338 | 0.797411731 | 0.424875879 | -0.981(-1.026 to -0.936) | <0.001 |
| Uganda | 0.835728075 | 1.167787432 | 0.587458613 | 0.23(0.136 to 0.323) | <0.001 |
| Ukraine | 1.004437447 | 1.299024607 | 0.746024877 | -0.408(-0.718 to -0.096) | 0.01 |
| United Arab Emirates | 4.036734854 | 5.537563715 | 3.01401229 | -0.961(-2.93 to 1.047) | 0.346 |
| United Kingdom | 1.027837013 | 1.079051651 | 0.929551462 | -0.488(-0.815 to -0.161) | 0.003 |
| United Republic of Tanzania | 0.86226948 | 1.19146867 | 0.562816822 | -0.02(-0.096 to 0.056) | 0.6 |
| United States of America | 0.868695713 | 0.914065582 | 0.791512479 | -1.353(-1.442 to -1.263) | <0.001 |
| United States Virgin Islands | 0.917913706 | 1.285368398 | 0.649821456 | -2.807(-3.175 to -2.438) | <0.001 |
| Uruguay | 3.924756852 | 4.266460409 | 3.579094528 | -1.313(-1.621 to -1.005) | <0.001 |
| Uzbekistan | 0.383678256 | 0.492310545 | 0.299387805 | 1.266(0.93 to 1.604) | <0.001 |
| Vanuatu | 0.551942723 | 0.74908585 | 0.373872016 | -0.536(-0.713 to -0.359) | <0.001 |
| Venezuela (Bolivarian Republic of) | 1.639177895 | 2.102895175 | 1.232211594 | -2.968(-3.465 to -2.469) | <0.001 |
| Viet Nam | 1.240373636 | 1.808814915 | 0.847077289 | 0.405(0.343 to 0.466) | <0.001 |
| Yemen | 1.060931768 | 1.571178299 | 0.693802847 | 0.378(0.217 to 0.539) | <0.001 |
| Zambia | 0.983081464 | 1.316900448 | 0.611102441 | 0.287(0.163 to 0.411) | <0.001 |
| Zimbabwe | 1.263033438 | 1.710650075 | 0.88803189 | 0.485(0.085 to 0.887) | 0.017 |
| GBTC, gallbladder and biliary tract cancer; ASMR, age-standardized mortality rate; AAPC, average annual percentage change;UI, Uncertainty interval; | | | | | |

| Supplementary Table 4. National ASDR of GBTC in 2021, and its temporal trend from 1990 to 2021 | | | | | |
| --- | --- | --- | --- | --- | --- |
| Nations | 2021 | | | 1990-2021 | |
|  | ASR per 100,000 | Upper 95%UI | Lower95%UI | AAPC (95CI%) | P value |
| Afghanistan | 47.1500434 | 74.42136044 | 21.49580948 | 0.739(0.647 to 0.83) | <0.001 |
| Albania | 25.5315643 | 37.15176781 | 17.19106378 | -0.951(-1.486 to -0.413) | 0.001 |
| Algeria | 67.41675665 | 87.59778611 | 49.49135708 | -0.856(-0.942 to -0.769) | <0.001 |
| American Samoa | 12.1438033 | 18.78899338 | 9.379879721 | -0.876(-1.395 to -0.354) | 0.001 |
| Andorra | 35.49882577 | 49.31761471 | 23.86233879 | -1.818(-2.199 to -1.436) | <0.001 |
| Angola | 9.266233409 | 12.80369194 | 6.116855907 | 0.425(0.197 to 0.654) | <0.001 |
| Antigua and Barbuda | 23.88203535 | 25.22829644 | 22.56330957 | -2.619(-3.645 to -1.581) | <0.001 |
| Argentina | 73.48521549 | 78.29078748 | 68.96613938 | -2.128(-2.433 to -1.822) | <0.001 |
| Armenia | 35.20307198 | 41.43510872 | 29.26842328 | 2.05(0.76 to 3.355) | 0.002 |
| Australia | 20.66331122 | 21.98180786 | 18.82452607 | -2.044(-2.78 to -1.303) | <0.001 |
| Austria | 33.33993149 | 35.7712415 | 30.41000858 | -3.195(-3.645 to -2.744) | <0.001 |
| Azerbaijan | 15.8749361 | 23.88155832 | 9.365319194 | -0.988(-1.545 to -0.429) | 0.001 |
| Bahamas | 36.00696472 | 44.30526426 | 29.25536988 | -1.696(-1.96 to -1.432) | <0.001 |
| Bahrain | 17.91358674 | 24.12193184 | 11.7391817 | -1.168(-1.523 to -0.811) | <0.001 |
| Bangladesh | 44.61054898 | 70.08485178 | 29.88818983 | 0.034(-0.301 to 0.37) | 0.844 |
| Barbados | 28.46792972 | 35.5921978 | 21.89278026 | -1.097(-1.489 to -0.704) | <0.001 |
| Belarus | 30.25092179 | 36.90139353 | 24.4392399 | 0.226(-0.39 to 0.846) | 0.473 |
| Belgium | 17.89390913 | 19.38078285 | 16.15634129 | -2.456(-2.821 to -2.09) | <0.001 |
| Belize | 25.14767927 | 28.44029661 | 22.01510514 | -1.604(-2.406 to -0.795) | <0.001 |
| Benin | 0.949598589 | 1.289000239 | 0.512352026 | 0.184(0.088 to 0.279) | <0.001 |
| Bermuda | 12.40056766 | 15.26115377 | 10.45460199 | -3.723(-4.018 to -3.428) | <0.001 |
| Bhutan | 50.70741326 | 74.21853058 | 34.67997425 | 0.195(0.128 to 0.261) | <0.001 |
| Bolivia (Plurinational State of) | 131.009934 | 186.329694 | 87.22230734 | -1.11(-1.202 to -1.018) | <0.001 |
| Bosnia and Herzegovina | 64.75089696 | 110.8604316 | 46.74875697 | -1.889(-2.248 to -1.528) | <0.001 |
| Botswana | 15.50669399 | 23.88735845 | 10.69036856 | -0.295(-1.184 to 0.602) | 0.518 |
| Brazil | 53.84228031 | 56.2046465 | 50.55656565 | -1.121(-1.352 to -0.889) | <0.001 |
| Brunei Darussalam | 53.35157572 | 68.13773436 | 41.07398518 | -1.561(-1.713 to -1.409) | <0.001 |
| Bulgaria | 29.54485833 | 34.73412705 | 24.97339855 | -0.815(-1.677 to 0.054) | 0.066 |
| Burkina Faso | 1.135664914 | 1.537316133 | 0.595613202 | 0.352(0.097 to 0.607) | 0.007 |
| Burundi | 18.15344801 | 26.79172173 | 10.25585479 | -1.173(-1.398 to -0.948) | <0.001 |
| Cabo Verde | 7.457940138 | 10.06055699 | 2.858393815 | 3.424(3.165 to 3.683) | <0.001 |
| Cambodia | 25.68294987 | 52.15291604 | 17.00137706 | -0.281(-0.355 to -0.207) | <0.001 |
| Cameroon | 1.208389015 | 1.703756609 | 0.614345518 | 0.288(0.218 to 0.357) | <0.001 |
| Canada | 19.36975688 | 20.72366933 | 17.72082549 | -2.507(-2.948 to -2.063) | <0.001 |
| Central African Republic | 8.681895042 | 14.41598044 | 5.326554434 | -0.322(-0.508 to -0.136) | 0.001 |
| Chad | 1.046688238 | 1.394522376 | 0.622340788 | 0.994(0.718 to 1.272) | <0.001 |
| Chile | 168.0260986 | 180.7001901 | 155.2170253 | -2.867(-3.242 to -2.491) | <0.001 |
| China | 40.38135342 | 52.61473151 | 28.2105878 | -0.849(-1.005 to -0.693) | <0.001 |
| Colombia | 54.79879462 | 64.23888381 | 45.65483565 | -2.186(-2.589 to -1.782) | <0.001 |
| Comoros | 19.36739119 | 30.12181874 | 12.13319764 | -0.071(-0.224 to 0.082) | 0.363 |
| Congo | 10.15947402 | 13.69896241 | 6.497585959 | -0.118(-0.317 to 0.081) | 0.245 |
| Cook Islands | 10.57951158 | 15.95734739 | 7.054863244 | -1.628(-2.127 to -1.127) | <0.001 |
| Costa Rica | 43.01932682 | 48.48003126 | 37.87445841 | -2.216(-2.679 to -1.751) | <0.001 |
| Coted'Ivoire | 1.119349089 | 1.498861417 | 0.552885836 | 0.484(0.324 to 0.644) | <0.001 |
| Croatia | 52.39411354 | 59.64284423 | 44.95533898 | -1.383(-1.988 to -0.774) | <0.001 |
| Cuba | 20.32354943 | 23.04180102 | 17.45396695 | -2.23(-2.808 to -1.65) | <0.001 |
| Cyprus | 25.79985554 | 33.47744251 | 19.39420018 | -2.502(-2.847 to -2.155) | <0.001 |
| Czechia | 73.94045958 | 85.38185728 | 62.93223798 | -2.62(-3.015 to -2.223) | <0.001 |
| Democratic People's Republic of Korea | 35.43108634 | 61.2306254 | 23.52871687 | -0.688(-0.713 to -0.663) | <0.001 |
| Democratic Republic of the Congo | 7.570641883 | 11.6985374 | 4.744342208 | 0.092(-0.059 to 0.244) | 0.232 |
| Denmark | 19.92530173 | 21.58730766 | 18.3675197 | -1.464(-1.865 to -1.06) | <0.001 |
| Djibouti | 18.9745078 | 31.1182513 | 11.14759314 | 0.067(0.013 to 0.122) | 0.015 |
| Dominica | 38.10593066 | 48.25556017 | 28.82355165 | -0.867(-1.002 to -0.732) | <0.001 |
| Dominican Republic | 19.8254037 | 27.9292941 | 14.98882848 | -0.86(-1.4 to -0.318) | 0.002 |
| Ecuador | 66.47992157 | 82.87367472 | 52.16727544 | -1.795(-2.208 to -1.38) | <0.001 |
| Egypt | 31.06315698 | 38.78437295 | 21.70887613 | 0.649(0.328 to 0.971) | <0.001 |
| El Salvador | 59.40606361 | 81.38017243 | 45.13325431 | -0.64(-0.908 to -0.372) | <0.001 |
| Equatorial Guinea | 9.082988281 | 13.90294319 | 5.472674021 | 0.188(-0.122 to 0.498) | 0.235 |
| Eritrea | 24.5240309 | 36.63720813 | 14.35493091 | 0.118(0.03 to 0.206) | 0.009 |
| Estonia | 33.96338908 | 38.41358485 | 29.23377339 | -1.054(-1.629 to -0.476) | <0.001 |
| Eswatini | 23.38835024 | 35.64396879 | 14.56998525 | 1.005(0.819 to 1.192) | <0.001 |
| Ethiopia | 31.53675562 | 45.17558408 | 21.50683055 | -1.357(-1.476 to -1.238) | <0.001 |
| Fiji | 25.540398 | 34.98727095 | 15.12858856 | 0.078(-0.255 to 0.412) | 0.647 |
| Finland | 36.24702655 | 39.25058238 | 32.77614606 | -1.933(-2.395 to -1.468) | <0.001 |
| France | 18.83772255 | 20.669529 | 16.98696839 | -3.192(-3.542 to -2.841) | <0.001 |
| Gabon | 9.911855471 | 13.49023324 | 6.24362084 | -0.211(-0.376 to -0.046) | 0.012 |
| Gambia | 0.000874046 | 0.001169421 | 0.000417384 | 0.175(-0.336 to 0.688) | 0.503 |
| Georgia | 35.97731595 | 43.82603395 | 28.84831292 | -0.841(-1.927 to 0.257) | 0.133 |
| Germany | 41.14838073 | 43.86429184 | 37.73268963 | -2.967(-3.472 to -2.46) | <0.001 |
| Ghana | 1.179450381 | 1.554039589 | 0.645800658 | 0.959(0.826 to 1.091) | <0.001 |
| Greece | 27.73417914 | 29.33028876 | 25.9244781 | -1.201(-1.85 to -0.548) | <0.001 |
| Greenland | 35.88763734 | 50.47180654 | 27.13740531 | -2.766(-3.058 to -2.473) | <0.001 |
| Grenada | 32.23428396 | 36.68618369 | 27.71089582 | -1.324(-1.513 to -1.134) | <0.001 |
| Guam | 7.032868742 | 9.275742337 | 5.51425429 | -1.266(-1.88 to -0.649) | <0.001 |
| Guatemala | 39.57542573 | 45.873338 | 33.89810124 | -3.566(-4.217 to -2.91) | <0.001 |
| Guinea | 1.018162138 | 1.431036487 | 0.564784627 | 0.559(0.47 to 0.647) | <0.001 |
| Guinea-Bissau | 1.456546433 | 2.068522343 | 0.847993039 | 0.504(0.391 to 0.618) | <0.001 |
| Guyana | 27.86478932 | 35.59919182 | 21.56703926 | -1.134(-1.729 to -0.535) | <0.001 |
| Haiti | 37.81400283 | 58.15571789 | 23.40847495 | -1.079(-1.152 to -1.006) | <0.001 |
| Honduras | 79.41857559 | 106.3281607 | 54.12414358 | 0.897(0.422 to 1.374) | <0.001 |
| Hungary | 64.15475671 | 72.82261985 | 55.67414872 | -2.938(-3.127 to -2.748) | <0.001 |
| Iceland | 18.6784323 | 20.56089143 | 16.53608654 | -2.126(-2.321 to -1.93) | <0.001 |
| India | 55.408205 | 65.32878978 | 37.4498993 | 1.038(0.781 to 1.294) | <0.001 |
| Indonesia | 16.85996077 | 35.22307928 | 11.53428877 | -0.502(-0.55 to -0.454) | <0.001 |
| Iran (Islamic Republic of) | 17.86468554 | 20.7546903 | 9.61435734 | 1.182(0.882 to 1.483) | <0.001 |
| Iraq | 23.82074704 | 30.94468845 | 16.31429603 | -0.35(-0.552 to -0.148) | 0.001 |
| Ireland | 14.71622698 | 16.13196153 | 13.06280834 | -2.801(-3.608 to -1.987) | <0.001 |
| Israel | 16.06406776 | 17.51578184 | 14.54299644 | -2.795(-3.852 to -1.726) | <0.001 |
| Italy | 47.95301198 | 51.113271 | 44.06080089 | -1.578(-1.816 to -1.34) | <0.001 |
| Jamaica | 24.73447666 | 32.04245947 | 18.77383914 | -0.752(-1.78 to 0.287) | 0.155 |
| Japan | 81.20217225 | 86.19559631 | 72.71290458 | -2.285(-2.444 to -2.127) | <0.001 |
| Jordan | 26.06427177 | 36.32669728 | 17.85986201 | -1.528(-1.98 to -1.075) | <0.001 |
| Kazakhstan | 26.34733842 | 31.02157945 | 22.00993998 | -0.704(-1.202 to -0.204) | 0.006 |
| Kenya | 27.18012692 | 38.92960486 | 19.51564461 | 0.43(0.297 to 0.562) | <0.001 |
| Kiribati | 15.66420589 | 21.51278999 | 7.311042857 | 0.021(-0.048 to 0.09) | 0.546 |
| Kuwait | 21.74468139 | 26.03297096 | 17.96938394 | -2.031(-4.406 to 0.403) | 0.101 |
| Kyrgyzstan | 25.44753611 | 30.93581377 | 20.82609102 | 0.003(-1.75 to 1.786) | 0.998 |
| Lao People's Democratic Republic | 20.76662833 | 44.17347252 | 13.26263753 | -1.137(-1.195 to -1.079) | <0.001 |
| Latvia | 27.28192285 | 31.39316412 | 23.7024569 | -0.266(-0.718 to 0.189) | 0.252 |
| Lebanon | 28.56411146 | 38.02367521 | 18.30426674 | -1.284(-1.49 to -1.077) | <0.001 |
| Lesotho | 23.67680494 | 35.49844579 | 15.64690744 | 2.14(1.866 to 2.414) | <0.001 |
| Liberia | 1.000777196 | 1.437208944 | 0.517012388 | 0.205(0.044 to 0.366) | 0.012 |
| Libya | 81.81128747 | 112.3389579 | 55.53914287 | -0.052(-0.306 to 0.204) | 0.692 |
| Lithuania | 39.86225255 | 45.23889844 | 34.59437707 | -0.271(-2.184 to 1.68) | 0.784 |
| Luxembourg | 19.19684525 | 21.19226777 | 17.24358259 | -2.897(-3.196 to -2.598) | <0.001 |
| Madagascar | 16.03463286 | 22.44126567 | 9.70197345 | -0.25(-0.344 to -0.155) | <0.001 |
| Malawi | 6.270879039 | 9.071792679 | 3.79119883 | -0.045(-0.177 to 0.087) | 0.503 |
| Malaysia | 25.20342046 | 30.76928925 | 15.56644048 | -0.69(-0.979 to -0.4) | <0.001 |
| Maldives | 11.65407069 | 15.08961713 | 8.355972262 | -2.674(-2.907 to -2.441) | <0.001 |
| Mali | 5.701720288 | 8.65952373 | 3.998609678 | -0.035(-0.168 to 0.099) | 0.61 |
| Malta | 14.93535629 | 16.64026328 | 13.31235337 | -2.528(-2.64 to -2.415) | <0.001 |
| Marshall Islands | 16.19444239 | 22.64203519 | 10.22127807 | -0.47(-0.553 to -0.388) | <0.001 |
| Mauritania | 0.94964608 | 1.35944702 | 0.521623301 | -0.151(-0.432 to 0.13) | 0.292 |
| Mauritius | 17.60013544 | 18.56788891 | 16.12358401 | -2.534(-4.757 to -0.259) | 0.029 |
| Mexico | 51.01178418 | 57.29662751 | 44.53509751 | -2.713(-3.079 to -2.345) | <0.001 |
| Micronesia (Federated States of) | 16.19372114 | 22.284687 | 10.63336119 | -0.877(-0.928 to -0.826) | <0.001 |
| Monaco | 12.25282305 | 16.2136982 | 8.996071324 | -0.695(-0.771 to -0.618) | <0.001 |
| Mongolia | 68.43018283 | 101.3344937 | 49.82282727 | -1.118(-1.427 to -0.807) | <0.001 |
| Montenegro | 31.40396509 | 42.35749694 | 22.60187006 | 0.056(-0.192 to 0.306) | 0.656 |
| Morocco | 15.71135549 | 20.45406948 | 9.244310226 | -0.027(-0.115 to 0.06) | 0.544 |
| Mozambique | 22.28671449 | 33.25991953 | 12.2131746 | 0.68(0.536 to 0.825) | <0.001 |
| Myanmar | 17.14891702 | 34.61937712 | 11.20013317 | -1.27(-1.345 to -1.194) | <0.001 |
| Namibia | 12.91476692 | 18.57109454 | 8.376760486 | 0.138(-0.049 to 0.326) | 0.149 |
| Nauru | 22.03918836 | 31.6799793 | 12.80765632 | -0.572(-0.671 to -0.473) | <0.001 |
| Nepal | 52.69972115 | 73.27339498 | 37.79818291 | 0.62(0.542 to 0.698) | <0.001 |
| Netherlands | 28.17502849 | 30.45643654 | 25.71065623 | -2.108(-2.384 to -1.831) | <0.001 |
| New Zealand | 33.99109576 | 36.67308151 | 30.79397708 | -0.11(-2.389 to 2.223) | 0.926 |
| Nicaragua | 50.07373931 | 78.15528476 | 39.39368501 | -1.407(-1.575 to -1.239) | <0.001 |
| Niger | 0.889501077 | 1.280376927 | 0.495739475 | 0.286(0.116 to 0.456) | 0.001 |
| Nigeria | 1.032883762 | 1.41998799 | 0.675168035 | 0.352(0.218 to 0.486) | <0.001 |
| Niue | 14.2211917 | 18.13493482 | 9.897175553 | -0.624(-0.731 to -0.516) | <0.001 |
| North Macedonia | 49.483637 | 64.16506732 | 36.51884478 | -0.839(-1.038 to -0.64) | <0.001 |
| Northern Mariana Islands | 9.084106694 | 15.98683037 | 7.168592223 | -0.403(-0.844 to 0.04) | 0.074 |
| Norway | 19.36031444 | 20.42641904 | 17.71237227 | -1.47(-2.205 to -0.73) | <0.001 |
| Oman | 14.57701867 | 19.81955343 | 10.49732835 | -1.299(-1.554 to -1.044) | <0.001 |
| Pakistan | 81.40971107 | 110.187588 | 59.22535407 | 0.405(0.362 to 0.448) | <0.001 |
| Palau | 9.872503644 | 13.8580157 | 6.541911439 | -0.422(-0.513 to -0.332) | <0.001 |
| Palestine | 28.29247038 | 35.0903623 | 18.0672769 | -0.969(-1.118 to -0.819) | <0.001 |
| Panama | 27.07862868 | 32.6613817 | 21.35874431 | -2.539(-3.47 to -1.599) | <0.001 |
| Papua New Guinea | 11.35666319 | 16.49046752 | 7.472972291 | -0.884(-0.975 to -0.793) | <0.001 |
| Paraguay | 42.99999548 | 59.17814891 | 29.95569569 | -0.055(-0.267 to 0.158) | 0.614 |
| Peru | 91.45238387 | 127.6367854 | 63.57451882 | -0.585(-1.372 to 0.208) | 0.148 |
| Philippines | 12.95447973 | 21.23046627 | 10.24894752 | 0.236(0.205 to 0.266) | <0.001 |
| Poland | 64.54409325 | 69.82208174 | 58.6851137 | -2.012(-2.292 to -1.732) | <0.001 |
| Portugal | 28.49293199 | 30.65772403 | 25.96824408 | -1.977(-2.252 to -1.701) | <0.001 |
| Puerto Rico | 15.00772496 | 17.79406357 | 12.28847481 | -2.403(-3.235 to -1.563) | <0.001 |
| Qatar | 26.91329866 | 39.73580708 | 18.73026561 | -1.748(-2.575 to -0.914) | <0.001 |
| Republic of Korea | 109.1864224 | 137.2531518 | 70.36075273 | -2.489(-2.577 to -2.401) | <0.001 |
| Republic of Moldova | 20.08720481 | 22.38231955 | 18.08871644 | -1.986(-3.436 to -0.516) | 0.008 |
| Romania | 35.10282176 | 39.54281302 | 30.72172824 | -1.649(-2.629 to -0.658) | 0.001 |
| Russian Federation | 31.47344764 | 34.05102641 | 28.92407077 | -0.624(-1.678 to 0.442) | 0.25 |
| Rwanda | 22.19584762 | 31.41304686 | 14.29043253 | -0.981(-1.092 to -0.871) | <0.001 |
| Saint Kitts and Nevis | 22.24201621 | 26.18243658 | 18.26591008 | -2.658(-3.141 to -2.172) | <0.001 |
| Saint Lucia | 19.06197807 | 22.95782303 | 15.47561961 | -2.455(-2.856 to -2.053) | <0.001 |
| Saint Vincent and the Grenadines | 20.88690802 | 23.79575807 | 18.41854226 | -2.249(-2.803 to -1.692) | <0.001 |
| Samoa | 14.98630096 | 19.67155217 | 10.28730189 | -0.49(-0.508 to -0.471) | <0.001 |
| San Marino | 25.70889148 | 38.41687791 | 15.53226893 | -2.843(-3.137 to -2.549) | <0.001 |
| Sao Tome and Principe | 33.32195443 | 43.884515 | 21.94893066 | 0.471(0.31 to 0.632) | <0.001 |
| Saudi Arabia | 36.24272607 | 54.3334871 | 27.09379179 | -0.239(-0.38 to -0.097) | 0.001 |
| Senegal | 1.040782249 | 1.492367616 | 0.580347978 | 0.491(0.307 to 0.676) | <0.001 |
| Serbia | 46.57354248 | 62.37728369 | 32.4882837 | -1.061(-1.395 to -0.726) | <0.001 |
| Seychelles | 19.53943283 | 28.80506553 | 15.80303149 | -2.157(-2.358 to -1.956) | <0.001 |
| Sierra Leone | 0.997736478 | 1.366397864 | 0.526864157 | 0.62(0.48 to 0.759) | <0.001 |
| Singapore | 18.96826539 | 20.47131003 | 17.10160858 | -2.43(-2.697 to -2.162) | <0.001 |
| Slovakia | 79.14379475 | 108.1979071 | 57.57030577 | -1.436(-1.571 to -1.3) | <0.001 |
| Slovenia | 46.28511279 | 53.71711371 | 38.96982149 | -2.248(-2.805 to -1.688) | <0.001 |
| Solomon Islands | 15.05065235 | 22.08685827 | 9.508429629 | -0.371(-0.544 to -0.198) | <0.001 |
| Somalia | 19.94454867 | 33.25522964 | 11.58956648 | 0.092(0.003 to 0.18) | 0.043 |
| South Africa | 19.44419009 | 22.80270302 | 12.91146475 | 0.523(-0.063 to 1.113) | 0.081 |
| South Sudan | 19.34147794 | 28.68775513 | 11.85512152 | 0.347(0.259 to 0.435) | <0.001 |
| Spain | 26.78238283 | 29.09847581 | 23.82326138 | -2.408(-2.675 to -2.141) | <0.001 |
| Sri Lanka | 16.76472177 | 30.13778757 | 10.14783994 | -3.874(-4.705 to -3.036) | <0.001 |
| Sudan | 21.93761482 | 31.98210155 | 14.77555447 | 0.016(-0.047 to 0.079) | 0.616 |
| Suriname | 22.55280139 | 30.60057379 | 16.36752807 | -1.173(-1.599 to -0.744) | <0.001 |
| Sweden | 50.08560347 | 55.93136493 | 44.2367653 | -2.016(-2.58 to -1.448) | <0.001 |
| Switzerland | 19.76214591 | 22.14801011 | 17.45165027 | -1.294(-1.899 to -0.686) | <0.001 |
| Syrian Arab Republic | 1.871833088 | 2.535181332 | 1.040094471 | 0.034(-0.317 to 0.387) | 0.848 |
| Taiwan (Province of China) | 47.69995868 | 51.76117425 | 43.35184101 | -0.94(-1.405 to -0.473) | <0.001 |
| Tajikistan | 2.767867158 | 3.857896002 | 1.876866262 | -1.637(-2.16 to -1.11) | <0.001 |
| Thailand | 150.280325 | 207.8210109 | 74.03032515 | -0.14(-0.376 to 0.096) | 0.244 |
| Timor-Leste | 15.74383717 | 32.8443427 | 10.31236377 | -0.731(-0.99 to -0.471) | <0.001 |
| Togo | 1.154600387 | 1.721305277 | 0.597820989 | 0.718(0.556 to 0.881) | <0.001 |
| Tokelau | 13.36406452 | 18.03072612 | 9.233142867 | -1.112(-1.198 to -1.025) | <0.001 |
| Tonga | 11.04548621 | 15.47054221 | 7.561680152 | -0.604(-0.73 to -0.479) | <0.001 |
| Trinidad and Tobago | 24.11727637 | 31.07331833 | 18.54052019 | -2.215(-2.555 to -1.872) | <0.001 |
| Tunisia | 46.29643163 | 64.18425804 | 30.88882152 | -0.413(-0.477 to -0.348) | <0.001 |
| Turkey | 29.16669405 | 39.09060596 | 21.53767103 | -1.775(-2.151 to -1.397) | <0.001 |
| Turkmenistan | 19.41656699 | 25.63360995 | 14.93303646 | -3.377(-5.999 to -0.682) | 0.014 |
| Tuvalu | 14.53497516 | 18.9290338 | 9.866905124 | -1.092(-1.137 to -1.047) | <0.001 |
| Uganda | 18.66815901 | 26.7705973 | 12.76803872 | 0.172(0.069 to 0.274) | 0.001 |
| Ukraine | 24.85568252 | 32.40444862 | 18.26808001 | -0.463(-0.631 to -0.295) | <0.001 |
| United Arab Emirates | 78.06523762 | 107.5741129 | 58.02941608 | -1.428(-2.882 to 0.049) | 0.058 |
| United Kingdom | 21.03253448 | 21.91604622 | 19.67937513 | -0.626(-0.944 to -0.308) | <0.001 |
| United Republic of Tanzania | 19.48811967 | 27.34420725 | 12.69583571 | -0.086(-0.174 to 0.001) | 0.053 |
| United States of America | 19.22992872 | 20.01880061 | 18.12223028 | -1.206(-1.306 to -1.107) | <0.001 |
| United States Virgin Islands | 21.66500211 | 29.69304011 | 15.47621786 | -2.73(-3.053 to -2.407) | <0.001 |
| Uruguay | 85.23939509 | 91.80538958 | 78.83908156 | -1.495(-1.977 to -1.01) | <0.001 |
| Uzbekistan | 9.251949465 | 11.89804395 | 7.174679763 | 1.33(1.027 to 1.635) | <0.001 |
| Vanuatu | 13.13437839 | 18.17821396 | 8.932083515 | -0.506(-0.698 to -0.314) | <0.001 |
| Venezuela (Bolivarian Republic of) | 37.55078754 | 49.20413841 | 27.51376714 | -3.069(-3.7 to -2.434) | <0.001 |
| Viet Nam | 27.23097597 | 40.81277192 | 18.43745126 | 0.282(0.231 to 0.334) | <0.001 |
| Yemen | 24.70671604 | 35.90432571 | 15.58671733 | 0.293(0.093 to 0.493) | 0.004 |
| Zambia | 22.52802065 | 30.91300617 | 13.75146592 | 0.155(0.046 to 0.264) | 0.005 |
| Zimbabwe | 29.96595436 | 41.42475439 | 20.63221466 | 0.674(0.246 to 1.104) | 0.002 |
| GBTC, gallbladder and biliary tract cancer; ASDR, age-standardized disability-adjusted life years rate; AAPC, average annual percentage change;UI, Uncertainty interval; | | | | | |

# Supplementary Table 5. Fitted longitudinal age-specific incidence rates of GBTC in global and five SDI regions from 1992 to 2021.

| **Location** | **Age** | **Middle age** | **Both**  Rate (95%CI) | **Male**  Rate (95%CI) | **Female**  Rate (95%CI) |
| --- | --- | --- | --- | --- | --- |
| Global | 20 to 24 | 22.5 | 0.007 (0.006-0.008) | 0.005 (0.004-0.006) | 0.01 (0.008-0.011) |
| Global | 25 to 29 | 27.5 | 0.015 (0.014-0.016) | 0.012 (0.011-0.013) | 0.019 (0.017-0.021) |
| Global | 30 to 34 | 32.5 | 0.036 (0.035-0.038) | 0.035 (0.034-0.037) | 0.037 (0.035-0.04) |
| Global | 35 to 39 | 37.5 | 0.085 (0.082-0.088) | 0.083 (0.08-0.086) | 0.087 (0.083-0.091) |
| Global | 40 to 44 | 42.5 | 0.176 (0.172-0.181) | 0.176 (0.172-0.181) | 0.176 (0.17-0.182) |
| Global | 45 to 49 | 47.5 | 0.318 (0.312-0.325) | 0.302 (0.296-0.308) | 0.334 (0.325-0.344) |
| Global | 50 to 54 | 52.5 | 0.587 (0.578-0.597) | 0.57 (0.56-0.58) | 0.604 (0.591-0.618) |
| Global | 55 to 59 | 57.5 | 1 (1-1) | 1 (1-1) | 1 (1-1) |
| Global | 60 to 64 | 62.5 | 1.544 (1.522-1.566) | 1.558 (1.535-1.58) | 1.529 (1.501-1.557) |
| Global | 65 to 69 | 67.5 | 2.258 (2.226-2.291) | 2.406 (2.371-2.441) | 2.131 (2.092-2.171) |
| Global | 70 to 74 | 72.5 | 3.206 (3.159-3.253) | 3.576 (3.523-3.63) | 2.905 (2.85-2.961) |
| Global | 75 to 79 | 77.5 | 4.366 (4.3-4.434) | 5.206 (5.125-5.289) | 3.752 (3.678-3.828) |
| Global | 80 to 84 | 82.5 | 5.824 (5.729-5.92) | 7.193 (7.071-7.317) | 4.884 (4.782-4.988) |
| Global | 85 to 89 | 87.5 | 7.686 (7.547-7.828) | 10.08 (9.885-10.28) | 6.221 (6.079-6.367) |
| Global | 90 to 94 | 92.5 | 9.475 (9.263-9.692) | 11.948 (11.636-12.268) | 7.809 (7.597-8.027) |
| Global | 95 plus | 97.5 | 10.348 (9.998-10.711) | 10.523 (10.017-11.055) | 9.039 (8.696-9.397) |
| High SDI | 20 to 24 | 22.5 | 0.007 (0.005-0.009) | 0.006 (0.004-0.008) | 0.008 (0.005-0.012) |
| High SDI | 25 to 29 | 27.5 | 0.016 (0.013-0.019) | 0.014 (0.012-0.017) | 0.017 (0.013-0.023) |
| High SDI | 30 to 34 | 32.5 | 0.041 (0.037-0.046) | 0.039 (0.035-0.044) | 0.043 (0.036-0.051) |
| High SDI | 35 to 39 | 37.5 | 0.096 (0.089-0.104) | 0.091 (0.085-0.098) | 0.101 (0.09-0.113) |
| High SDI | 40 to 44 | 42.5 | 0.202 (0.191-0.213) | 0.189 (0.18-0.199) | 0.216 (0.199-0.233) |
| High SDI | 45 to 49 | 47.5 | 0.344 (0.33-0.358) | 0.322 (0.31-0.335) | 0.367 (0.346-0.39) |
| High SDI | 50 to 54 | 52.5 | 0.591 (0.572-0.611) | 0.571 (0.553-0.589) | 0.613 (0.584-0.643) |
| High SDI | 55 to 59 | 57.5 | 1 (1-1) | 1 (1-1) | 1 (1-1) |
| High SDI | 60 to 64 | 62.5 | 1.522 (1.482-1.563) | 1.57 (1.531-1.61) | 1.474 (1.418-1.531) |
| High SDI | 65 to 69 | 67.5 | 2.33 (2.27-2.392) | 2.524 (2.462-2.588) | 2.149 (2.07-2.232) |
| High SDI | 70 to 74 | 72.5 | 3.315 (3.229-3.404) | 3.743 (3.65-3.839) | 2.947 (2.837-3.06) |
| High SDI | 75 to 79 | 77.5 | 4.62 (4.497-4.746) | 5.424 (5.284-5.567) | 3.991 (3.841-4.148) |
| High SDI | 80 to 84 | 82.5 | 6.213 (6.042-6.389) | 7.521 (7.318-7.73) | 5.286 (5.082-5.499) |
| High SDI | 85 to 89 | 87.5 | 8.116 (7.878-8.36) | 10.076 (9.779-10.381) | 6.853 (6.575-7.142) |
| High SDI | 90 to 94 | 92.5 | 9.959 (9.629-10.299) | 11.834 (11.407-12.277) | 8.603 (8.224-9) |
| High SDI | 95 plus | 97.5 | 10.995 (10.514-11.497) | 10.967 (10.322-11.652) | 9.924 (9.388-10.49) |
| High-middle SDI | 20 to 24 | 22.5 | 0.007 (0.006-0.008) | 0.005 (0.004-0.006) | 0.01 (0.008-0.012) |
| High-middle SDI | 25 to 29 | 27.5 | 0.014 (0.013-0.016) | 0.012 (0.01-0.013) | 0.018 (0.015-0.021) |
| High-middle SDI | 30 to 34 | 32.5 | 0.037 (0.034-0.04) | 0.036 (0.032-0.039) | 0.038 (0.034-0.042) |
| High-middle SDI | 35 to 39 | 37.5 | 0.091 (0.087-0.095) | 0.088 (0.082-0.094) | 0.093 (0.087-0.1) |
| High-middle SDI | 40 to 44 | 42.5 | 0.192 (0.185-0.199) | 0.191 (0.182-0.201) | 0.19 (0.18-0.2) |
| High-middle SDI | 45 to 49 | 47.5 | 0.33 (0.321-0.34) | 0.308 (0.296-0.32) | 0.355 (0.341-0.369) |
| High-middle SDI | 50 to 54 | 52.5 | 0.592 (0.579-0.606) | 0.575 (0.557-0.594) | 0.609 (0.589-0.629) |
| High-middle SDI | 55 to 59 | 57.5 | 1 (1-1) | 1 (1-1) | 1 (1-1) |
| High-middle SDI | 60 to 64 | 62.5 | 1.505 (1.477-1.534) | 1.508 (1.467-1.55) | 1.502 (1.463-1.543) |
| High-middle SDI | 65 to 69 | 67.5 | 2.19 (2.149-2.233) | 2.305 (2.242-2.371) | 2.094 (2.039-2.151) |
| High-middle SDI | 70 to 74 | 72.5 | 3.125 (3.064-3.188) | 3.398 (3.3-3.498) | 2.906 (2.828-2.986) |
| High-middle SDI | 75 to 79 | 77.5 | 4.222 (4.135-4.31) | 4.941 (4.792-5.095) | 3.721 (3.617-3.827) |
| High-middle SDI | 80 to 84 | 82.5 | 5.223 (5.108-5.342) | 6.404 (6.191-6.624) | 4.48 (4.347-4.616) |
| High-middle SDI | 85 to 89 | 87.5 | 6.248 (6.09-6.411) | 8.737 (8.396-9.093) | 5.007 (4.84-5.18) |
| High-middle SDI | 90 to 94 | 92.5 | 6.955 (6.72-7.199) | 9.747 (9.193-10.334) | 5.636 (5.397-5.886) |
| High-middle SDI | 95 plus | 97.5 | 6.699 (6.296-7.128) | 7.916 (6.975-8.984) | 5.759 (5.358-6.19) |
| Middle SDI | 20 to 24 | 22.5 | 0.007 (0.007-0.009) | 0.005 (0.004-0.006) | 0.01 (0.009-0.012) |
| Middle SDI | 25 to 29 | 27.5 | 0.016 (0.015-0.018) | 0.013 (0.011-0.015) | 0.02 (0.018-0.023) |
| Middle SDI | 30 to 34 | 32.5 | 0.038 (0.036-0.041) | 0.037 (0.034-0.04) | 0.04 (0.037-0.044) |
| Middle SDI | 35 to 39 | 37.5 | 0.089 (0.085-0.093) | 0.086 (0.081-0.091) | 0.091 (0.086-0.097) |
| Middle SDI | 40 to 44 | 42.5 | 0.179 (0.173-0.185) | 0.179 (0.171-0.188) | 0.177 (0.168-0.185) |
| Middle SDI | 45 to 49 | 47.5 | 0.317 (0.309-0.326) | 0.302 (0.291-0.314) | 0.333 (0.321-0.346) |
| Middle SDI | 50 to 54 | 52.5 | 0.598 (0.585-0.611) | 0.578 (0.56-0.596) | 0.617 (0.599-0.636) |
| Middle SDI | 55 to 59 | 57.5 | 1 (1-1) | 1 (1-1) | 1 (1-1) |
| Middle SDI | 60 to 64 | 62.5 | 1.537 (1.508-1.566) | 1.544 (1.502-1.588) | 1.527 (1.488-1.567) |
| Middle SDI | 65 to 69 | 67.5 | 2.168 (2.126-2.211) | 2.258 (2.194-2.324) | 2.086 (2.031-2.142) |
| Middle SDI | 70 to 74 | 72.5 | 2.995 (2.934-3.057) | 3.308 (3.209-3.411) | 2.737 (2.662-2.814) |
| Middle SDI | 75 to 79 | 77.5 | 3.843 (3.759-3.929) | 4.566 (4.418-4.718) | 3.307 (3.21-3.407) |
| Middle SDI | 80 to 84 | 82.5 | 4.595 (4.482-4.711) | 5.622 (5.414-5.838) | 3.861 (3.735-3.992) |
| Middle SDI | 85 to 89 | 87.5 | 5.491 (5.326-5.66) | 7.536 (7.193-7.895) | 4.268 (4.1-4.444) |
| Middle SDI | 90 to 94 | 92.5 | 5.573 (5.324-5.833) | 8.061 (7.495-8.67) | 4.22 (3.979-4.475) |
| Middle SDI | 95 plus | 97.5 | 4.709 (4.308-5.148) | 5.525 (4.704-6.489) | 3.969 (3.566-4.419) |
| Low-middle SDI | 20 to 24 | 22.5 | 0.007 (0.006-0.008) | 0.005 (0.004-0.007) | 0.009 (0.007-0.01) |
| Low-middle SDI | 25 to 29 | 27.5 | 0.015 (0.013-0.017) | 0.012 (0.01-0.014) | 0.017 (0.015-0.019) |
| Low-middle SDI | 30 to 34 | 32.5 | 0.03 (0.028-0.033) | 0.03 (0.026-0.035) | 0.031 (0.028-0.034) |
| Low-middle SDI | 35 to 39 | 37.5 | 0.068 (0.064-0.072) | 0.068 (0.061-0.075) | 0.069 (0.064-0.074) |
| Low-middle SDI | 40 to 44 | 42.5 | 0.139 (0.132-0.145) | 0.143 (0.132-0.154) | 0.138 (0.131-0.147) |
| Low-middle SDI | 45 to 49 | 47.5 | 0.286 (0.275-0.296) | 0.278 (0.262-0.296) | 0.294 (0.281-0.307) |
| Low-middle SDI | 50 to 54 | 52.5 | 0.571 (0.555-0.589) | 0.558 (0.531-0.587) | 0.584 (0.563-0.606) |
| Low-middle SDI | 55 to 59 | 57.5 | 1 (1-1) | 1 (1-1) | 1 (1-1) |
| Low-middle SDI | 60 to 64 | 62.5 | 1.631 (1.59-1.673) | 1.624 (1.556-1.696) | 1.621 (1.571-1.673) |
| Low-middle SDI | 65 to 69 | 67.5 | 2.249 (2.191-2.31) | 2.384 (2.28-2.492) | 2.146 (2.077-2.219) |
| Low-middle SDI | 70 to 74 | 72.5 | 3.015 (2.931-3.102) | 3.205 (3.056-3.361) | 2.847 (2.748-2.949) |
| Low-middle SDI | 75 to 79 | 77.5 | 3.488 (3.38-3.6) | 4.024 (3.819-4.241) | 3.107 (2.987-3.233) |
| Low-middle SDI | 80 to 84 | 82.5 | 4.507 (4.348-4.673) | 5.13 (4.829-5.449) | 4.005 (3.829-4.189) |
| Low-middle SDI | 85 to 89 | 87.5 | 5.319 (5.08-5.569) | 6.327 (5.861-6.83) | 4.556 (4.302-4.826) |
| Low-middle SDI | 90 to 94 | 92.5 | 5.671 (5.288-6.083) | 6.925 (6.157-7.788) | 4.717 (4.324-5.147) |
| Low-middle SDI | 95 plus | 97.5 | 4.539 (3.957-5.208) | 5.006 (3.889-6.445) | 3.847 (3.264-4.535) |
| Low SDI | 20 to 24 | 22.5 | 0.008 (0.006-0.011) | 0.005 (0.003-0.01) | 0.009 (0.007-0.013) |
| Low SDI | 25 to 29 | 27.5 | 0.016 (0.013-0.019) | 0.012 (0.008-0.019) | 0.018 (0.014-0.023) |
| Low SDI | 30 to 34 | 32.5 | 0.031 (0.026-0.036) | 0.032 (0.024-0.042) | 0.03 (0.025-0.037) |
| Low SDI | 35 to 39 | 37.5 | 0.069 (0.061-0.078) | 0.071 (0.058-0.088) | 0.069 (0.059-0.08) |
| Low SDI | 40 to 44 | 42.5 | 0.137 (0.125-0.151) | 0.149 (0.126-0.175) | 0.133 (0.118-0.149) |
| Low SDI | 45 to 49 | 47.5 | 0.28 (0.26-0.302) | 0.274 (0.24-0.313) | 0.284 (0.259-0.311) |
| Low SDI | 50 to 54 | 52.5 | 0.55 (0.518-0.585) | 0.56 (0.503-0.625) | 0.549 (0.51-0.592) |
| Low SDI | 55 to 59 | 57.5 | 1 (1-1) | 1 (1-1) | 1 (1-1) |
| Low SDI | 60 to 64 | 62.5 | 1.712 (1.626-1.803) | 1.717 (1.566-1.881) | 1.702 (1.599-1.811) |
| Low SDI | 65 to 69 | 67.5 | 2.395 (2.27-2.527) | 2.544 (2.315-2.796) | 2.307 (2.162-2.462) |
| Low SDI | 70 to 74 | 72.5 | 3.253 (3.073-3.443) | 3.495 (3.162-3.863) | 3.089 (2.883-3.31) |
| Low SDI | 75 to 79 | 77.5 | 3.753 (3.52-4.002) | 4.351 (3.893-4.863) | 3.387 (3.131-3.665) |
| Low SDI | 80 to 84 | 82.5 | 4.749 (4.405-5.121) | 5.549 (4.875-6.316) | 4.218 (3.845-4.628) |
| Low SDI | 85 to 89 | 87.5 | 5.691 (5.146-6.295) | 7.17 (6.058-8.487) | 4.801 (4.233-5.445) |
| Low SDI | 90 to 94 | 92.5 | 6.024 (5.083-7.139) | 7.594 (5.719-10.085) | 4.945 (3.998-6.117) |
| Low SDI | 95 plus | 97.5 | 4.592 (3.067-6.874) | 5.793 (2.829-11.865) | 3.54 (2.142-5.848) |

# Supplementary Table 6. Period rate ratio of GBTC in global and five SDI regions from 1992 to 2021

| **Location** | **Period** | **Middle period** | **Both**  RR (95% CI) | **Male**  RR (95% CI) | **Female**  RR (95% CI) |
| --- | --- | --- | --- | --- | --- |
| Global | 1992 to 1996 | 1,994.5 | 1.094 (1.079-1.109) | 0.993 (0.979-1.007) | 1.18 (1.16-1.2) |
| Global | 1997 to 2001 | 1,999.5 | 1.046 (1.033-1.058) | 0.978 (0.966-0.991) | 1.101 (1.085-1.118) |
| Global | 2002 to 2006 | 2,004.5 | 1.01 (0.999-1.02) | 0.975 (0.964-0.986) | 1.038 (1.023-1.052) |
| Global | 2007 to 2011 | 2,009.5 | 1 (1-1) | 1 (1-1) | 1 (1-1) |
| Global | 2012 to 2016 | 2,014.5 | 0.983 (0.974-0.993) | 1.007 (0.997-1.018) | 0.965 (0.953-0.978) |
| Global | 2017 to 2021 | 2,019.5 | 0.989 (0.978-0.999) | 1.03 (1.019-1.041) | 0.958 (0.945-0.971) |
| High SDI | 1992 to 1996 | 1,994.5 | 1.189 (1.159-1.221) | 1.079 (1.052-1.107) | 1.302 (1.254-1.352) |
| High SDI | 1997 to 2001 | 1,999.5 | 1.101 (1.078-1.125) | 1.041 (1.02-1.064) | 1.163 (1.129-1.199) |
| High SDI | 2002 to 2006 | 2,004.5 | 1.024 (1.006-1.042) | 0.999 (0.981-1.017) | 1.05 (1.025-1.076) |
| High SDI | 2007 to 2011 | 2,009.5 | 1 (1-1) | 1 (1-1) | 1 (1-1) |
| High SDI | 2012 to 2016 | 2,014.5 | 0.974 (0.958-0.991) | 0.983 (0.967-1) | 0.962 (0.94-0.985) |
| High SDI | 2017 to 2021 | 2,019.5 | 0.97 (0.951-0.989) | 0.994 (0.975-1.013) | 0.944 (0.918-0.971) |
| High-middle SDI | 1992 to 1996 | 1,994.5 | 1.043 (1.023-1.063) | 0.913 (0.887-0.939) | 1.173 (1.143-1.204) |
| High-middle SDI | 1997 to 2001 | 1,999.5 | 1.02 (1.004-1.037) | 0.932 (0.909-0.956) | 1.105 (1.081-1.131) |
| High-middle SDI | 2002 to 2006 | 2,004.5 | 0.998 (0.984-1.013) | 0.952 (0.931-0.973) | 1.041 (1.021-1.062) |
| High-middle SDI | 2007 to 2011 | 2,009.5 | 1 (1-1) | 1 (1-1) | 1 (1-1) |
| High-middle SDI | 2012 to 2016 | 2,014.5 | 0.977 (0.964-0.991) | 1.022 (1.001-1.043) | 0.937 (0.919-0.955) |
| High-middle SDI | 2017 to 2021 | 2,019.5 | 0.989 (0.975-1.004) | 1.051 (1.028-1.073) | 0.932 (0.913-0.952) |
| Middle SDI | 1992 to 1996 | 1,994.5 | 1.073 (1.052-1.095) | 0.95 (0.921-0.979) | 1.192 (1.16-1.224) |
| Middle SDI | 1997 to 2001 | 1,999.5 | 1.02 (1.002-1.039) | 0.927 (0.902-0.953) | 1.106 (1.08-1.133) |
| Middle SDI | 2002 to 2006 | 2,004.5 | 1.005 (0.989-1.022) | 0.959 (0.936-0.982) | 1.048 (1.025-1.071) |
| Middle SDI | 2007 to 2011 | 2,009.5 | 1 (1-1) | 1 (1-1) | 1 (1-1) |
| Middle SDI | 2012 to 2016 | 2,014.5 | 1.006 (0.991-1.021) | 1.041 (1.018-1.064) | 0.975 (0.955-0.995) |
| Middle SDI | 2017 to 2021 | 2,019.5 | 1.042 (1.026-1.058) | 1.091 (1.067-1.116) | 1 (0.98-1.022) |
| Low-middle SDI | 1992 to 1996 | 1,994.5 | 0.929 (0.904-0.955) | 0.904 (0.862-0.948) | 0.954 (0.922-0.987) |
| Low-middle SDI | 1997 to 2001 | 1,999.5 | 0.942 (0.918-0.965) | 0.923 (0.885-0.963) | 0.959 (0.93-0.989) |
| Low-middle SDI | 2002 to 2006 | 2,004.5 | 0.972 (0.951-0.995) | 0.955 (0.92-0.992) | 0.985 (0.958-1.013) |
| Low-middle SDI | 2007 to 2011 | 2,009.5 | 1 (1-1) | 1 (1-1) | 1 (1-1) |
| Low-middle SDI | 2012 to 2016 | 2,014.5 | 1.028 (1.007-1.049) | 1.025 (0.99-1.062) | 1.026 (1-1.053) |
| Low-middle SDI | 2017 to 2021 | 2,019.5 | 1.064 (1.042-1.087) | 1.074 (1.037-1.114) | 1.052 (1.025-1.08) |
| Low SDI | 1992 to 1996 | 1,994.5 | 0.986 (0.93-1.046) | 0.946 (0.834-1.073) | 1 (0.93-1.075) |
| Low SDI | 1997 to 2001 | 1,999.5 | 0.971 (0.921-1.023) | 0.95 (0.857-1.053) | 0.974 (0.913-1.039) |
| Low SDI | 2002 to 2006 | 2,004.5 | 0.99 (0.944-1.037) | 0.965 (0.886-1.051) | 0.999 (0.944-1.059) |
| Low SDI | 2007 to 2011 | 2,009.5 | 1 (1-1) | 1 (1-1) | 1 (1-1) |
| Low SDI | 2012 to 2016 | 2,014.5 | 1.061 (1.016-1.108) | 1.051 (0.972-1.137) | 1.07 (1.015-1.128) |
| Low SDI | 2017 to 2021 | 2,019.5 | 1.096 (1.048-1.146) | 1.103 (1.006-1.21) | 1.099 (1.04-1.162) |

# Supplementary Table 7. Cohort rate ratio of GBTC in global and five SDI regions from 1992 to 2021

| **Location** | **Cohort** | **Middle cohort** | **Both**  RR (95% CI) | **Male**  RR (95% CI) | **Female**  RR (95% CI) |
| --- | --- | --- | --- | --- | --- |
| Global | 1892 to 1901 | 1,897.0 | 0.87 (0.766-0.989) | 0.673 (0.551-0.823) | 1.035 (0.902-1.186) |
| Global | 1897 to 1906 | 1,902.0 | 1.019 (0.967-1.075) | 0.733 (0.681-0.789) | 1.245 (1.173-1.321) |
| Global | 1902 to 1911 | 1,907.0 | 1.129 (1.095-1.164) | 0.82 (0.79-0.852) | 1.386 (1.337-1.438) |
| Global | 1907 to 1916 | 1,912.0 | 1.224 (1.197-1.252) | 0.921 (0.898-0.946) | 1.486 (1.446-1.528) |
| Global | 1912 to 1921 | 1,917.0 | 1.207 (1.184-1.231) | 0.95 (0.93-0.971) | 1.439 (1.405-1.475) |
| Global | 1917 to 1926 | 1,922.0 | 1.202 (1.181-1.223) | 0.977 (0.959-0.996) | 1.408 (1.378-1.44) |
| Global | 1922 to 1931 | 1,927.0 | 1.181 (1.163-1.2) | 1.012 (0.996-1.029) | 1.339 (1.312-1.367) |
| Global | 1927 to 1936 | 1,932.0 | 1.151 (1.134-1.168) | 1.023 (1.008-1.039) | 1.269 (1.244-1.293) |
| Global | 1932 to 1941 | 1,937.0 | 1.105 (1.089-1.121) | 1.016 (1.001-1.032) | 1.185 (1.163-1.207) |
| Global | 1937 to 1946 | 1,942.0 | 1.075 (1.059-1.09) | 1.013 (0.999-1.028) | 1.129 (1.108-1.151) |
| Global | 1942 to 1951 | 1,947.0 | 1.033 (1.019-1.048) | 1.003 (0.989-1.018) | 1.06 (1.04-1.08) |
| Global | 1947 to 1956 | 1,952.0 | 1 (1-1) | 1 (1-1) | 1 (1-1) |
| Global | 1952 to 1961 | 1,957.0 | 0.966 (0.95-0.982) | 0.989 (0.973-1.005) | 0.946 (0.926-0.966) |
| Global | 1957 to 1966 | 1,962.0 | 0.936 (0.918-0.954) | 0.97 (0.952-0.989) | 0.906 (0.883-0.93) |
| Global | 1962 to 1971 | 1,967.0 | 0.91 (0.888-0.932) | 0.981 (0.958-1.005) | 0.848 (0.821-0.875) |
| Global | 1967 to 1976 | 1,972.0 | 0.899 (0.872-0.928) | 0.984 (0.955-1.015) | 0.825 (0.791-0.861) |
| Global | 1972 to 1981 | 1,977.0 | 0.899 (0.862-0.938) | 0.998 (0.958-1.039) | 0.813 (0.767-0.861) |
| Global | 1977 to 1986 | 1,982.0 | 0.893 (0.842-0.948) | 1.015 (0.958-1.076) | 0.79 (0.728-0.856) |
| Global | 1982 to 1991 | 1,987.0 | 0.916 (0.84-0.998) | 1.086 (0.998-1.182) | 0.777 (0.691-0.873) |
| Global | 1987 to 1996 | 1,992.0 | 0.886 (0.772-1.016) | 1.05 (0.909-1.213) | 0.759 (0.637-0.905) |
| Global | 1992 to 2001 | 1,997.0 | 0.837 (0.657-1.067) | 1.012 (0.776-1.318) | 0.713 (0.527-0.963) |
| High SDI | 1892 to 1901 | 1,897.0 | 0.856 (0.735-0.998) | 0.756 (0.595-0.96) | 1.019 (0.857-1.211) |
| High SDI | 1897 to 1906 | 1,902.0 | 1.065 (0.996-1.14) | 0.851 (0.776-0.933) | 1.304 (1.203-1.414) |
| High SDI | 1902 to 1911 | 1,907.0 | 1.233 (1.182-1.287) | 0.958 (0.911-1.008) | 1.532 (1.451-1.619) |
| High SDI | 1907 to 1916 | 1,912.0 | 1.372 (1.326-1.419) | 1.056 (1.017-1.095) | 1.72 (1.643-1.8) |
| High SDI | 1912 to 1921 | 1,917.0 | 1.401 (1.359-1.446) | 1.111 (1.076-1.146) | 1.736 (1.663-1.813) |
| High SDI | 1917 to 1926 | 1,922.0 | 1.38 (1.341-1.421) | 1.105 (1.074-1.137) | 1.7 (1.633-1.77) |
| High SDI | 1922 to 1931 | 1,927.0 | 1.386 (1.349-1.424) | 1.169 (1.139-1.201) | 1.645 (1.583-1.71) |
| High SDI | 1927 to 1936 | 1,932.0 | 1.351 (1.316-1.387) | 1.192 (1.162-1.223) | 1.538 (1.481-1.598) |
| High SDI | 1932 to 1941 | 1,937.0 | 1.266 (1.233-1.299) | 1.159 (1.13-1.187) | 1.388 (1.337-1.441) |
| High SDI | 1937 to 1946 | 1,942.0 | 1.174 (1.144-1.205) | 1.112 (1.085-1.14) | 1.241 (1.195-1.289) |
| High SDI | 1942 to 1951 | 1,947.0 | 1.045 (1.018-1.073) | 1.02 (0.996-1.046) | 1.07 (1.029-1.112) |
| High SDI | 1947 to 1956 | 1,952.0 | 1 (1-1) | 1 (1-1) | 1 (1-1) |
| High SDI | 1952 to 1961 | 1,957.0 | 0.914 (0.885-0.944) | 0.928 (0.901-0.956) | 0.9 (0.859-0.944) |
| High SDI | 1957 to 1966 | 1,962.0 | 0.847 (0.814-0.881) | 0.873 (0.842-0.906) | 0.821 (0.775-0.87) |
| High SDI | 1962 to 1971 | 1,967.0 | 0.796 (0.756-0.838) | 0.83 (0.791-0.871) | 0.762 (0.706-0.822) |
| High SDI | 1967 to 1976 | 1,972.0 | 0.776 (0.724-0.831) | 0.818 (0.767-0.872) | 0.734 (0.663-0.813) |
| High SDI | 1972 to 1981 | 1,977.0 | 0.773 (0.703-0.851) | 0.819 (0.749-0.895) | 0.729 (0.632-0.84) |
| High SDI | 1977 to 1986 | 1,982.0 | 0.796 (0.693-0.914) | 0.876 (0.772-0.994) | 0.714 (0.578-0.881) |
| High SDI | 1982 to 1991 | 1,987.0 | 0.831 (0.671-1.028) | 0.936 (0.772-1.136) | 0.724 (0.522-1.005) |
| High SDI | 1987 to 1996 | 1,992.0 | 0.792 (0.547-1.146) | 0.908 (0.646-1.277) | 0.663 (0.378-1.161) |
| High SDI | 1992 to 2001 | 1,997.0 | 0.723 (0.355-1.472) | 0.857 (0.445-1.65) | 0.609 (0.21-1.769) |
| High-middle SDI | 1892 to 1901 | 1,897.0 | 1.161 (0.937-1.44) | 0.677 (0.402-1.141) | 1.506 (1.189-1.908) |
| High-middle SDI | 1897 to 1906 | 1,902.0 | 1.12 (1.028-1.219) | 0.734 (0.617-0.872) | 1.438 (1.303-1.587) |
| High-middle SDI | 1902 to 1911 | 1,907.0 | 1.113 (1.062-1.166) | 0.775 (0.712-0.843) | 1.415 (1.337-1.498) |
| High-middle SDI | 1907 to 1916 | 1,912.0 | 1.168 (1.13-1.206) | 0.856 (0.81-0.904) | 1.452 (1.394-1.513) |
| High-middle SDI | 1912 to 1921 | 1,917.0 | 1.168 (1.137-1.2) | 0.902 (0.863-0.942) | 1.418 (1.369-1.47) |
| High-middle SDI | 1917 to 1926 | 1,922.0 | 1.152 (1.125-1.18) | 0.925 (0.891-0.959) | 1.368 (1.326-1.412) |
| High-middle SDI | 1922 to 1931 | 1,927.0 | 1.121 (1.097-1.145) | 0.928 (0.898-0.958) | 1.306 (1.269-1.344) |
| High-middle SDI | 1927 to 1936 | 1,932.0 | 1.116 (1.093-1.138) | 0.946 (0.918-0.975) | 1.278 (1.243-1.313) |
| High-middle SDI | 1932 to 1941 | 1,937.0 | 1.074 (1.053-1.095) | 0.955 (0.928-0.982) | 1.189 (1.157-1.221) |
| High-middle SDI | 1937 to 1946 | 1,942.0 | 1.075 (1.055-1.097) | 0.985 (0.958-1.013) | 1.162 (1.132-1.194) |
| High-middle SDI | 1942 to 1951 | 1,947.0 | 1.045 (1.025-1.066) | 0.999 (0.971-1.027) | 1.09 (1.061-1.12) |
| High-middle SDI | 1947 to 1956 | 1,952.0 | 1 (1-1) | 1 (1-1) | 1 (1-1) |
| High-middle SDI | 1952 to 1961 | 1,957.0 | 0.98 (0.959-1.002) | 1.016 (0.985-1.048) | 0.946 (0.917-0.976) |
| High-middle SDI | 1957 to 1966 | 1,962.0 | 0.945 (0.92-0.97) | 1.016 (0.979-1.053) | 0.877 (0.845-0.911) |
| High-middle SDI | 1962 to 1971 | 1,967.0 | 0.943 (0.913-0.974) | 1.072 (1.025-1.12) | 0.82 (0.782-0.86) |
| High-middle SDI | 1967 to 1976 | 1,972.0 | 0.936 (0.897-0.977) | 1.106 (1.044-1.171) | 0.774 (0.726-0.825) |
| High-middle SDI | 1972 to 1981 | 1,977.0 | 0.982 (0.927-1.041) | 1.2 (1.112-1.295) | 0.768 (0.701-0.841) |
| High-middle SDI | 1977 to 1986 | 1,982.0 | 1.02 (0.938-1.108) | 1.306 (1.173-1.454) | 0.739 (0.647-0.845) |
| High-middle SDI | 1982 to 1991 | 1,987.0 | 1.093 (0.967-1.235) | 1.489 (1.273-1.741) | 0.733 (0.601-0.894) |
| High-middle SDI | 1987 to 1996 | 1,992.0 | 1.078 (0.873-1.332) | 1.491 (1.123-1.979) | 0.741 (0.539-1.018) |
| High-middle SDI | 1992 to 2001 | 1,997.0 | 1.053 (0.707-1.57) | 1.567 (0.91-2.701) | 0.677 (0.375-1.221) |
| Middle SDI | 1892 to 1901 | 1,897.0 | 0.932 (0.655-1.326) | 0.698 (0.368-1.323) | 1.156 (0.757-1.763) |
| Middle SDI | 1897 to 1906 | 1,902.0 | 1.012 (0.89-1.151) | 0.676 (0.542-0.842) | 1.344 (1.147-1.575) |
| Middle SDI | 1902 to 1911 | 1,907.0 | 1.047 (0.981-1.117) | 0.718 (0.644-0.799) | 1.37 (1.263-1.487) |
| Middle SDI | 1907 to 1916 | 1,912.0 | 1.04 (0.996-1.086) | 0.78 (0.728-0.836) | 1.292 (1.223-1.366) |
| Middle SDI | 1912 to 1921 | 1,917.0 | 1.026 (0.993-1.06) | 0.797 (0.757-0.839) | 1.248 (1.196-1.302) |
| Middle SDI | 1917 to 1926 | 1,922.0 | 1.026 (0.998-1.054) | 0.818 (0.784-0.853) | 1.227 (1.183-1.272) |
| Middle SDI | 1922 to 1931 | 1,927.0 | 1.031 (1.007-1.056) | 0.851 (0.821-0.882) | 1.206 (1.168-1.245) |
| Middle SDI | 1927 to 1936 | 1,932.0 | 1.049 (1.026-1.071) | 0.891 (0.863-0.921) | 1.2 (1.166-1.235) |
| Middle SDI | 1932 to 1941 | 1,937.0 | 1.052 (1.031-1.073) | 0.926 (0.898-0.954) | 1.171 (1.139-1.204) |
| Middle SDI | 1937 to 1946 | 1,942.0 | 1.043 (1.023-1.064) | 0.954 (0.927-0.982) | 1.127 (1.097-1.157) |
| Middle SDI | 1942 to 1951 | 1,947.0 | 1.031 (1.011-1.051) | 0.984 (0.957-1.012) | 1.074 (1.046-1.103) |
| Middle SDI | 1947 to 1956 | 1,952.0 | 1 (1-1) | 1 (1-1) | 1 (1-1) |
| Middle SDI | 1952 to 1961 | 1,957.0 | 0.992 (0.971-1.014) | 1.031 (1-1.064) | 0.957 (0.929-0.986) |
| Middle SDI | 1957 to 1966 | 1,962.0 | 0.974 (0.95-0.998) | 1.042 (1.005-1.08) | 0.912 (0.881-0.945) |
| Middle SDI | 1962 to 1971 | 1,967.0 | 0.971 (0.942-1) | 1.099 (1.053-1.146) | 0.858 (0.823-0.896) |
| Middle SDI | 1967 to 1976 | 1,972.0 | 0.981 (0.944-1.02) | 1.133 (1.073-1.196) | 0.848 (0.802-0.896) |
| Middle SDI | 1972 to 1981 | 1,977.0 | 0.976 (0.926-1.028) | 1.154 (1.075-1.239) | 0.816 (0.756-0.881) |
| Middle SDI | 1977 to 1986 | 1,982.0 | 0.943 (0.877-1.015) | 1.152 (1.042-1.274) | 0.766 (0.688-0.853) |
| Middle SDI | 1982 to 1991 | 1,987.0 | 0.955 (0.859-1.061) | 1.231 (1.065-1.422) | 0.737 (0.632-0.86) |
| Middle SDI | 1987 to 1996 | 1,992.0 | 0.944 (0.797-1.118) | 1.237 (0.969-1.579) | 0.726 (0.574-0.918) |
| Middle SDI | 1992 to 2001 | 1,997.0 | 0.921 (0.68-1.246) | 1.242 (0.793-1.945) | 0.666 (0.439-1.01) |
| Low-middle SDI | 1892 to 1901 | 1,897.0 | 0.62 (0.367-1.048) | 0.497 (0.181-1.368) | 0.724 (0.392-1.337) |
| Low-middle SDI | 1897 to 1906 | 1,902.0 | 0.633 (0.518-0.773) | 0.574 (0.409-0.804) | 0.713 (0.557-0.912) |
| Low-middle SDI | 1902 to 1911 | 1,907.0 | 0.679 (0.614-0.752) | 0.627 (0.529-0.742) | 0.738 (0.649-0.838) |
| Low-middle SDI | 1907 to 1916 | 1,912.0 | 0.718 (0.672-0.767) | 0.683 (0.612-0.762) | 0.764 (0.703-0.83) |
| Low-middle SDI | 1912 to 1921 | 1,917.0 | 0.768 (0.731-0.807) | 0.734 (0.676-0.797) | 0.809 (0.759-0.861) |
| Low-middle SDI | 1917 to 1926 | 1,922.0 | 0.806 (0.775-0.839) | 0.784 (0.734-0.838) | 0.836 (0.795-0.879) |
| Low-middle SDI | 1922 to 1931 | 1,927.0 | 0.844 (0.815-0.873) | 0.828 (0.782-0.876) | 0.87 (0.834-0.908) |
| Low-middle SDI | 1927 to 1936 | 1,932.0 | 0.894 (0.868-0.922) | 0.879 (0.836-0.924) | 0.915 (0.881-0.95) |
| Low-middle SDI | 1932 to 1941 | 1,937.0 | 0.926 (0.9-0.952) | 0.913 (0.871-0.957) | 0.936 (0.904-0.969) |
| Low-middle SDI | 1937 to 1946 | 1,942.0 | 0.955 (0.93-0.981) | 0.951 (0.909-0.995) | 0.962 (0.931-0.995) |
| Low-middle SDI | 1942 to 1951 | 1,947.0 | 0.979 (0.954-1.005) | 0.982 (0.94-1.027) | 0.983 (0.952-1.016) |
| Low-middle SDI | 1947 to 1956 | 1,952.0 | 1 (1-1) | 1 (1-1) | 1 (1-1) |
| Low-middle SDI | 1952 to 1961 | 1,957.0 | 1.006 (0.977-1.035) | 1.028 (0.979-1.079) | 0.99 (0.955-1.026) |
| Low-middle SDI | 1957 to 1966 | 1,962.0 | 1.063 (1.028-1.099) | 1.061 (1.003-1.123) | 1.057 (1.014-1.101) |
| Low-middle SDI | 1962 to 1971 | 1,967.0 | 1.045 (1.002-1.089) | 1.067 (0.995-1.143) | 1.022 (0.971-1.076) |
| Low-middle SDI | 1967 to 1976 | 1,972.0 | 1.064 (1.009-1.121) | 1.091 (0.998-1.192) | 1.033 (0.968-1.104) |
| Low-middle SDI | 1972 to 1981 | 1,977.0 | 1.091 (1.018-1.169) | 1.133 (1.01-1.272) | 1.053 (0.966-1.148) |
| Low-middle SDI | 1977 to 1986 | 1,982.0 | 1.067 (0.972-1.172) | 1.101 (0.939-1.29) | 1.034 (0.921-1.161) |
| Low-middle SDI | 1982 to 1991 | 1,987.0 | 1.065 (0.935-1.214) | 1.122 (0.894-1.406) | 1.024 (0.873-1.201) |
| Low-middle SDI | 1987 to 1996 | 1,992.0 | 1.043 (0.864-1.26) | 1.101 (0.773-1.567) | 1.002 (0.801-1.252) |
| Low-middle SDI | 1992 to 2001 | 1,997.0 | 1.005 (0.735-1.373) | 1.108 (0.606-2.026) | 0.947 (0.657-1.365) |
| Low SDI | 1892 to 1901 | 1,897.0 | 0.553 (0.131-2.344) | 0.093 (0-47.433) | 0.418 (0.055-3.164) |
| Low SDI | 1897 to 1906 | 1,902.0 | 0.506 (0.292-0.876) | 0.523 (0.208-1.314) | 0.523 (0.264-1.037) |
| Low SDI | 1902 to 1911 | 1,907.0 | 0.604 (0.471-0.774) | 0.585 (0.386-0.887) | 0.622 (0.455-0.849) |
| Low SDI | 1907 to 1916 | 1,912.0 | 0.642 (0.552-0.746) | 0.659 (0.513-0.847) | 0.643 (0.532-0.776) |
| Low SDI | 1912 to 1921 | 1,917.0 | 0.694 (0.624-0.772) | 0.725 (0.608-0.865) | 0.696 (0.609-0.794) |
| Low SDI | 1917 to 1926 | 1,922.0 | 0.755 (0.694-0.82) | 0.781 (0.679-0.899) | 0.755 (0.681-0.837) |
| Low SDI | 1922 to 1931 | 1,927.0 | 0.821 (0.766-0.88) | 0.843 (0.749-0.949) | 0.83 (0.762-0.904) |
| Low SDI | 1927 to 1936 | 1,932.0 | 0.9 (0.847-0.957) | 0.906 (0.815-1.007) | 0.913 (0.848-0.984) |
| Low SDI | 1932 to 1941 | 1,937.0 | 0.965 (0.912-1.021) | 0.957 (0.866-1.056) | 0.976 (0.911-1.045) |
| Low SDI | 1937 to 1946 | 1,942.0 | 1.001 (0.948-1.057) | 0.99 (0.9-1.089) | 1.012 (0.948-1.081) |
| Low SDI | 1942 to 1951 | 1,947.0 | 1.022 (0.969-1.077) | 1.016 (0.926-1.115) | 1.031 (0.967-1.099) |
| Low SDI | 1947 to 1956 | 1,952.0 | 1 (1-1) | 1 (1-1) | 1 (1-1) |
| Low SDI | 1952 to 1961 | 1,957.0 | 0.965 (0.909-1.024) | 0.983 (0.885-1.092) | 0.954 (0.888-1.026) |
| Low SDI | 1957 to 1966 | 1,962.0 | 0.991 (0.924-1.062) | 0.987 (0.872-1.116) | 0.986 (0.906-1.073) |
| Low SDI | 1962 to 1971 | 1,967.0 | 0.924 (0.848-1.008) | 0.945 (0.812-1.099) | 0.909 (0.819-1.01) |
| Low SDI | 1967 to 1976 | 1,972.0 | 0.93 (0.835-1.037) | 0.964 (0.798-1.165) | 0.911 (0.799-1.04) |
| Low SDI | 1972 to 1981 | 1,977.0 | 0.937 (0.815-1.076) | 0.964 (0.757-1.227) | 0.903 (0.762-1.071) |
| Low SDI | 1977 to 1986 | 1,982.0 | 0.924 (0.77-1.109) | 0.941 (0.678-1.306) | 0.913 (0.733-1.137) |
| Low SDI | 1982 to 1991 | 1,987.0 | 0.914 (0.712-1.174) | 0.919 (0.577-1.463) | 0.907 (0.675-1.218) |
| Low SDI | 1987 to 1996 | 1,992.0 | 0.849 (0.597-1.206) | 0.961 (0.483-1.913) | 0.811 (0.541-1.216) |
| Low SDI | 1992 to 2001 | 1,997.0 | 0.825 (0.478-1.425) | 0.868 (0.274-2.749) | 0.806 (0.433-1.501) |

| Supplementary Table 8. Decomposition analysis for global and region GBTC burden from 1990 to 2021 | | | | | |
| --- | --- | --- | --- | --- | --- |
| Location | Measures | Overll difference | Population aging | Population expansion | Epidemiological change |
| Global | Deaths | 73278.06 | 41700.51 (56.91%) | 71526.81 (97.61%) | -39949.27 (-54.52%) |
|  | DALYs | 1406032.25 | 757241.54 (53.86%) | 1617706.29 (115.05%) | -968915.59 (-68.91%) |
|  | Incidence | 108970.59 | 47135.33 (43.26%) | 83543.6 (76.67%) | -21708.41 (-19.92%) |
| High SDI | Deaths | 12438.48 | 18088.98 (145.43%) | 14853.89 (119.42%) | -20504.38 (-164.85%) |
|  | DALYs | 69462.68 | 274777.8 (395.58%) | 284983.27 (410.27%) | -490298.38 (-705.84%) |
|  | Incidence | 29777.19 | 22822.42 (76.64%) | 19888.73 (66.79%) | -12933.96 (-43.44%) |
| High-middle SDI | Deaths | 13087.33 | 12732.21 (97.29%) | 12153.65 (92.87%) | -11798.53 (-90.15%) |
|  | DALYs | 227892.83 | 238204.12 (104.52%) | 275539.48 (120.91%) | -285850.78 (-125.43%) |
|  | Incidence | 25416.11 | 14238.93 (56.02%) | 14304.42 (56.28%) | -3127.24 (-12.3%) |
| Middle SDI | Deaths | 26408.36 | 14525.42 (55%) | 18214.21 (68.97%) | -6331.28 (-23.97%) |
|  | DALYs | 582368.11 | 303039.35 (52.04%) | 455047.67 (78.14%) | -175718.9 (-30.17%) |
|  | Incidence | 33026.09 | 14562.36 (44.09%) | 19130.59 (57.93%) | -666.87 (-2.02%) |
| Low-middle SDI | Deaths | 17352.19 | 3149.43 (18.15%) | 11501.61 (66.28%) | 2701.16 (15.57%) |
|  | DALYs | 426288.9 | 64733.82 (15.19%) | 302950.49 (71.07%) | 58604.59 (13.75%) |
|  | Incidence | 16905.77 | 2864.57 (16.94%) | 11102.9 (65.68%) | 2938.3 (17.38%) |
| Low SDI | Deaths | 3994.99 | -214.92 (-5.38%) | 3498.65 (87.58%) | 711.26 (17.8%) |
|  | DALYs | 100292.66 | -6394.87 (-6.38%) | 94280.42 (94.01%) | 12407.11 (12.37%) |
|  | Incidence | 3831.61 | -220.81 (-5.76%) | 3359.8 (87.69%) | 692.63 (18.08%) |
| GBTC, gallbladder and biliary tract cancer; SDI, socio-demographic index; | | | | | |

| Supplementary Table 9. Slope index for global and region GBTC-related DALYs in 1990 and 2021 | | | | |
| --- | --- | --- | --- | --- |
| Regions | Year | Slope index | 95%CI lower | 95%CI upper |
| All included | 1990 | 41.015 | 30.607 | 51.423 |
| All included | 2021 | 17.148 | 10.526 | 23.770 |
| Regions |  | | | |
| Eastern Sub-Saharan Africa | 1990 | 0.150 | -9.324 | 9.624 |
| Eastern Sub-Saharan Africa | 2021 | 1.783 | -5.018 | 8.585 |
| Western Sub-Saharan Africa | 1990 | 0.129 | -0.173 | 0.431 |
| Western Sub-Saharan Africa | 2021 | 0.407 | 0.064 | 0.749 |
| Central Sub-Saharan Africa | 1990 | 1.736 | -0.329 | 3.800 |
| Central Sub-Saharan Africa | 2021 | 1.599 | -0.021 | 3.219 |
| North Africa and Middle East | 1990 | 25.647 | 4.961 | 46.332 |
| North Africa and Middle East | 2021 | 1.828 | -18.423 | 22.079 |
| Oceania | 1990 | -0.563 | -10.430 | 9.305 |
| Oceania | 2021 | -1.737 | -15.024 | 11.549 |
| South Asia | 1990 | -9.148 | -23.770 | 5.475 |
| South Asia | 2021 | 7.196 | -11.637 | 26.029 |
| Caribbean | 1990 | 7.381 | -7.686 | 22.447 |
| Caribbean | 2021 | -7.373 | -21.576 | 6.830 |
| Southeast Asia | 1990 | 13.533 | 0.511 | 26.556 |
| Southeast Asia | 2021 | 1.563 | -9.473 | 12.599 |
| Southern Sub-Saharan Africa | 1990 | 1.423 | -16.089 | 18.935 |
| Southern Sub-Saharan Africa | 2021 | -12.736 | -42.412 | 16.940 |
| Central Latin America | 1990 | -6.134 | -44.420 | 32.151 |
| Central Latin America | 2021 | -18.521 | -42.906 | 5.864 |
| Central Asia | 1990 | 17.587 | -47.056 | 82.231 |
| Central Asia | 2021 | 16.582 | -10.582 | 43.746 |
| East Asia | 1990 | 4.650 | -66.401 | 75.701 |
| East Asia | 2021 | 12.480 | 6.483 | 18.477 |
| Andean Latin America | 1990 | -86.877 | -205.246 | 31.492 |
| Andean Latin America | 2021 | -47.189 | -221.809 | 127.431 |
| Central Europe | 1990 | 34.361 | -41.944 | 110.665 |
| Central Europe | 2021 | 23.428 | -4.853 | 51.710 |
| Eastern Europe | 1990 | 7.127 | -8.270 | 22.523 |
| Eastern Europe | 2021 | 11.086 | 0.647 | 21.525 |
| Southern Latin America | 1990 | -113.299 | -1063.018 | 836.420 |
| Southern Latin America | 2021 | 104.150 | -23.701 | 232.001 |
| Western Europe | 1990 | -5.902 | -36.404 | 24.600 |
| Western Europe | 2021 | -3.459 | -17.606 | 10.688 |
| High-income North America | 1990 | -46.289 | -135.708 | 43.130 |
| High-income North America | 2021 | -17.070 | -38.121 | 3.981 |
| High-income Asia Pacific | 1990 | 139.514 | -188.910 | 467.938 |
| High-income Asia Pacific | 2021 | 86.903 | 31.652 | 142.154 |
| Sub-Saharan Africa | 1990 | 6.678 | -3.088 | 16.443 |
| Sub-Saharan Africa | 2021 | 8.574 | -0.589 | 17.736 |
| Southeast Asia, east Asia, and Oceania | 1990 | 2.343 | -6.700 | 11.385 |
| Southeast Asia, east Asia, and Oceania | 2021 | -2.359 | -8.293 | 3.576 |
| Latin America and Caribbean | 1990 | 2.736 | -27.618 | 33.089 |
| Latin America and Caribbean | 2021 | -11.958 | -30.326 | 6.410 |
| Central Europe, eastern Europe, and central Asia | 1990 | 27.275 | -18.006 | 72.556 |
| Central Europe, eastern Europe, and central Asia | 2021 | 28.044 | 7.239 | 48.849 |
| High income | 1990 | -11.192 | -37.523 | 15.139 |
| High income | 2021 | -2.551 | -13.650 | 8.549 |
| Socio-demographic index Regions |  | | | |
| Low SDI | 1990 | 4.875 | -11.248 | 20.999 |
| Low SDI | 2021 | 10.909 | -2.481 | 24.300 |
| Low-middle SDI | 1990 | -3.367 | -21.675 | 14.940 |
| Low-middle SDI | 2021 | 4.393 | -14.066 | 22.852 |
| Middle SDI | 1990 | 22.567 | -4.200 | 49.335 |
| Middle SDI | 2021 | 8.259 | -5.105 | 21.623 |
| High-middle SDI | 1990 | 6.425 | -44.164 | 57.014 |
| High-middle SDI | 2021 | -5.525 | -28.772 | 17.723 |
| High SDI | 1990 | -14.593 | -46.507 | 17.322 |
| High SDI | 2021 | -5.424 | -19.058 | 8.210 |
| GBTC, biliary tract cancer; SDI, socio-demographic index ; | | | | |

| Supplementary Table 10. Projections of GBTC incidence up to 2040 stratified by sex | | | | | | |
| --- | --- | --- | --- | --- | --- | --- |
| Year | Total | | Male | | Female | |
|  | Predicted Number (95%UI) | Predicted ASR per 10^5^ population (95% UI) | Predicted Number (95%UI) | Predicted ASR per 10^5^ population (95% UI) | Predicted Number (95%UI) | Predicted ASR per 10^5^ population (95% UI) |
| 2022 | 224760 (215214.1-234305.9) | 2.551 (2.443-2.66) | 105078.3 (100900.5-109256.1) | 2.639 (2.589-2.688) | 119681.7 (114313.6-125049.8) | 2.484 (2.433-2.535) |
| 2023 | 230389.4 (219281.8-241497) | 2.543 (2.42-2.666) | 108079.8 (103133.6-113025.9) | 2.638 (2.561-2.715) | 122309.7 (116148.2-128471.1) | 2.469 (2.393-2.545) |
| 2024 | 236427.3 (222905.1-249949.6) | 2.534 (2.389-2.68) | 111287.9 (105154.4-117421.5) | 2.637 (2.525-2.748) | 125139.4 (117750.7-132528.1) | 2.454 (2.348-2.561) |
| 2025 | 242679.9 (225916-259443.9) | 2.526 (2.351-2.701) | 114608.7 (106885.5-122331.9) | 2.635 (2.484-2.786) | 128071.2 (119030.4-137112) | 2.439 (2.298-2.581) |
| 2026 | 249047.8 (228260.8-269834.8) | 2.518 (2.308-2.729) | 117986.3 (108294.4-127678.2) | 2.634 (2.439-2.829) | 131061.5 (119966.4-142156.5) | 2.425 (2.245-2.606) |
| 2027 | 255404.4 (229859.5-280949.2) | 2.512 (2.26-2.764) | 121364.3 (109344.1-133384.5) | 2.633 (2.39-2.876) | 134040.1 (120515.4-147564.8) | 2.412 (2.189-2.634) |
| 2028 | 261948.7 (230953.2-292944.1) | 2.506 (2.208-2.803) | 124838.8 (110148.2-139529.3) | 2.633 (2.338-2.928) | 137109.9 (120805-153414.8) | 2.398 (2.131-2.666) |
| 2029 | 268864.9 (231722.2-306007.6) | 2.5 (2.154-2.846) | 128500.4 (110792.5-146208.3) | 2.632 (2.282-2.982) | 140364.5 (120929.6-159799.4) | 2.386 (2.07-2.701) |
| 2030 | 276012.4 (232031-319993.9) | 2.495 (2.096-2.893) | 132280.7 (111208.4-153353.1) | 2.631 (2.224-3.039) | 143731.7 (120822.7-166640.8) | 2.373 (2.008-2.738) |
| 2031 | 283314.8 (231786.6-334843) | 2.49 (2.037-2.944) | 136140.6 (111346.3-160934.9) | 2.631 (2.163-3.099) | 147174.2 (120440.3-173908.1) | 2.361 (1.944-2.778) |
| 2032 | 290643.8 (230853.9-350433.8) | 2.487 (1.975-3) | 140024.9 (111144.1-168905.7) | 2.631 (2.099-3.164) | 150618.9 (119709.8-181528.1) | 2.35 (1.879-2.82) |
| 2033 | 298149.6 (229357.1-366942.1) | 2.485 (1.911-3.059) | 144001.2 (110655.4-177347.1) | 2.632 (2.033-3.231) | 154148.4 (118701.7-189595) | 2.339 (1.812-2.865) |
| 2034 | 306004.7 (227407-384602.5) | 2.483 (1.844-3.122) | 148154.8 (109932.9-186376.7) | 2.632 (1.964-3.3) | 157849.9 (117474.1-198225.7) | 2.328 (1.744-2.912) |
| 2035 | 314098.1 (224876.5-403319.7) | 2.482 (1.776-3.188) | 152436 (108916-195956.1) | 2.632 (1.892-3.372) | 161662 (115960.5-207363.6) | 2.317 (1.674-2.96) |
| 2036 | 322358.2 (221662.4-423054) | 2.482 (1.706-3.259) | 156814.5 (107555.1-206073.8) | 2.633 (1.819-3.447) | 165543.7 (114107.3-216980.2) | 2.308 (1.604-3.012) |
| 2037 | 330654.6 (217624.3-443684.9) | 2.484 (1.634-3.335) | 161236.4 (105785.3-216687.4) | 2.635 (1.744-3.526) | 169418.2 (111839-226997.5) | 2.299 (1.532-3.065) |
| 2038 | 339116.3 (212830.7-465401.9) | 2.487 (1.559-3.415) | 165752.3 (103628.2-227876.4) | 2.637 (1.666-3.607) | 173364 (109202.5-237525.5) | 2.29 (1.46-3.121) |
| 2039 | 347912.5 (207346.2-488478.8) | 2.491 (1.483-3.499) | 170443.4 (101111.3-239775.5) | 2.638 (1.586-3.69) | 177469.1 (106235-248703.3) | 2.282 (1.386-3.178) |
| 2040 | 356945.4 (201049.4-512841.4) | 2.495 (1.404-3.587) | 175269.3 (98176.7-252361.8) | 2.64 (1.504-3.775) | 181676.2 (102872.7-260479.6) | 2.274 (1.311-3.237) |
| GBTC, gallbladder and biliary tract cancer; ASR, age-standardized rate; UI, Uncertainty interval; | | | | | | |

| Supplementary Table 11. Projections of GBTC deaths up to 2040 stratified by sex | | | | | | |
| --- | --- | --- | --- | --- | --- | --- |
| Year | Total | | Male | | Female | |
|  | Predicted Number (95%UI) | Predicted ASR per 10^5^ population (95% UI) | Predicted Number (95%UI) | Predicted ASR per 10^5^ population (95% UI) | Predicted Number (95%UI) | Predicted ASR per 10^5^ population (95% UI) |
| 2022 | 178089.7 (170247.8-185931.5) | 2.029 (1.939-2.119) | 79124.2 (75910.6-82337.9) | 2.015 (1.978-2.053) | 98965.4 (94337.3-103593.6) | 2.484 (2.433-2.535) |
| 2023 | 182051.2 (173018.9-191083.5) | 2.016 (1.915-2.116) | 81172 (77427.6-84916.4) | 2.008 (1.951-2.065) | 100879.2 (95591.3-106167.1) | 2.469 (2.393-2.545) |
| 2024 | 186338.1 (175466.3-197209.9) | 2.002 (1.885-2.12) | 83382.3 (78819.3-87945.3) | 2.001 (1.919-2.082) | 102955.8 (96647-109264.6) | 2.454 (2.348-2.561) |
| 2025 | 190769.4 (177429.2-204109.5) | 1.989 (1.849-2.128) | 85665.7 (80006.3-91325.2) | 1.993 (1.883-2.102) | 105103.7 (97423-112784.4) | 2.439 (2.298-2.581) |
| 2026 | 195243.9 (178845.4-211642.5) | 1.976 (1.81-2.142) | 87962.4 (80945.6-94979.3) | 1.985 (1.844-2.125) | 107281.5 (97899.8-116663.2) | 2.425 (2.245-2.606) |
| 2027 | 199659.4 (179653.8-219665) | 1.964 (1.766-2.161) | 90223.4 (81604.7-98842) | 1.977 (1.803-2.151) | 109436.1 (98049.1-120823) | 2.412 (2.189-2.634) |
| 2028 | 204199.1 (180071.7-228326.5) | 1.952 (1.721-2.183) | 92542.8 (82090.6-102995) | 1.969 (1.759-2.179) | 111656.3 (97981-125331.5) | 2.398 (2.131-2.666) |
| 2029 | 209017.2 (180252.9-237781.5) | 1.94 (1.673-2.208) | 95000 (82479.8-107520.1) | 1.961 (1.714-2.209) | 114017.2 (97773.2-130261.3) | 2.386 (2.07-2.701) |
| 2030 | 213985.5 (180080.1-247890.9) | 1.929 (1.623-2.235) | 97529.2 (82710.1-112348.4) | 1.953 (1.666-2.24) | 116456.3 (97370-135542.5) | 2.373 (2.008-2.738) |
| 2031 | 219023.2 (179468.4-258577.9) | 1.918 (1.571-2.265) | 100083.3 (82732.7-117434) | 1.945 (1.617-2.273) | 118939.9 (96735.8-141143.9) | 2.361 (1.944-2.778) |
| 2032 | 224031.8 (178321.3-269742.2) | 1.909 (1.518-2.299) | 102615.1 (82500.7-122729.5) | 1.937 (1.566-2.308) | 121416.7 (95820.6-147012.8) | 2.35 (1.879-2.82) |
| 2033 | 229159.5 (176765.4-281553.5) | 1.899 (1.464-2.334) | 105199.3 (82078-128320.5) | 1.929 (1.513-2.344) | 123960.2 (94687.4-153233) | 2.339 (1.812-2.865) |
| 2034 | 234545.3 (174894.7-294195.8) | 1.89 (1.409-2.372) | 107909.1 (81515.2-134303) | 1.921 (1.459-2.382) | 126636.2 (93379.5-159892.9) | 2.328 (1.744-2.912) |
| 2035 | 240078 (172596.3-307559.8) | 1.882 (1.352-2.411) | 110690.9 (80757.3-140624.5) | 1.913 (1.404-2.421) | 129387.1 (91839-166935.3) | 2.317 (1.674-2.96) |
| 2036 | 245670.8 (169774.8-321566.8) | 1.874 (1.294-2.454) | 113498.4 (79755.1-147241.7) | 1.905 (1.348-2.461) | 132172.4 (90019.7-174325.1) | 2.308 (1.604-3.012) |
| 2037 | 251218.2 (166328.7-336107.7) | 1.868 (1.236-2.5) | 116281.6 (78460.2-154103) | 1.897 (1.291-2.503) | 134936.6 (87868.5-182004.8) | 2.299 (1.532-3.065) |
| 2038 | 256861.2 (162346.2-351376.2) | 1.862 (1.176-2.548) | 119107.1 (76915.3-161298.8) | 1.889 (1.232-2.545) | 137754.1 (85430.9-190077.3) | 2.29 (1.46-3.121) |
| 2039 | 262738.7 (157889.1-367588.2) | 1.856 (1.114-2.598) | 122047 (75155.2-168938.8) | 1.881 (1.173-2.589) | 140691.7 (82734-198649.4) | 2.282 (1.386-3.178) |
| 2040 | 268745.5 (152852.4-384638.6) | 1.851 (1.052-2.651) | 125052.4 (73130.1-176974.6) | 1.873 (1.112-2.633) | 143693.1 (79722.3-207664) | 2.274 (1.311-3.237) |
| GBTC, gallbladder and biliary tract cancer; ASR, age-standardized rate; UI, Uncertainty interval; | | | | | | |

| Supplementary Table 12. Projections of GBTC DALYs up to 2040 stratified by sex | | | | | | |
| --- | --- | --- | --- | --- | --- | --- |
| Year | Total | | Male | | Female | |
|  | Predicted Number (95%UI) | Predicted ASR per 10^5^ population (95% UI) | Predicted Number (95%UI) | Predicted ASR per 10^5^ population (95% UI) | Predicted Number (95%UI) | Predicted ASR per 10^5^ population (95% UI) |
| 2022 | 3841575.3 (3637486.5-4045664.2) | 42.94 (40.657-45.223) | 1745492.3 (1673852.9-1817131.6) | 41.851 (41.032-42.67) | 2096083.1 (1963633.6-2228532.6) | 44.078 (42.865-45.291) |
| 2023 | 3916317.5 (3689266.9-4143368.1) | 42.67 (40.194-45.146) | 1782517.5 (1700045.9-1864989.1) | 41.655 (40.453-42.857) | 2133799.9 (1989220.9-2278379) | 43.742 (42.139-45.345) |
| 2024 | 3993858.7 (3731343.1-4256374.2) | 42.402 (39.612-45.191) | 1820791.6 (1721380.4-1920202.9) | 41.452 (39.759-43.146) | 2173067 (2009962.8-2336171.3) | 43.411 (41.296-45.526) |
| 2025 | 4072296.5 (3761588.1-4383004.9) | 42.133 (38.916-45.35) | 1859341 (1737045.5-1981636.4) | 41.237 (38.975-43.5) | 2212955.6 (2024542.6-2401368.5) | 43.084 (40.367-45.801) |
| 2026 | 4151292.1 (3780120.9-4522463.3) | 41.877 (38.13-45.623) | 1898013.7 (1747289.7-2048737.6) | 41.023 (38.128-43.919) | 2253278.4 (2032831.1-2473725.7) | 42.772 (39.382-46.163) |
| 2027 | 4230073.2 (3786955.9-4673190.5) | 41.64 (37.275-46.005) | 1936566.9 (1752292-2120841.8) | 40.823 (37.237-44.409) | 2293506.3 (2034663.9-2552348.7) | 42.478 (38.355-46.601) |
| 2028 | 4309745.1 (3784094.5-4835395.7) | 41.415 (36.361-46.469) | 1975422.3 (1752950.9-2197893.7) | 40.627 (36.304-44.95) | 2334322.8 (2031143.6-2637502) | 42.194 (37.289-47.1) |
| 2029 | 4391232.4 (3772935.4-5009529.3) | 41.194 (35.39-46.998) | 2014948.8 (1749856.7-2280041) | 40.422 (35.323-45.522) | 2376283.5 (2023078.8-2729488.3) | 41.915 (36.185-47.645) |
| 2030 | 4473070.2 (3752453-5193687.4) | 40.975 (34.369-47.58) | 2054362.9 (1742411.8-2366313.9) | 40.205 (34.295-46.115) | 2418707.4 (2010041.3-2827373.5) | 41.639 (35.047-48.231) |
| 2031 | 4555572.2 (3722923.5-5388221) | 40.77 (33.313-48.227) | 2093862.9 (1730749.6-2456976.2) | 39.988 (33.233-46.744) | 2461709.3 (1992173.9-2931244.8) | 41.378 (33.887-48.869) |
| 2032 | 4638530.4 (3684093.9-5592967) | 40.589 (32.231-48.946) | 2133583.2 (1714895.8-2552270.5) | 39.787 (32.15-47.424) | 2504947.3 (1969198-3040696.5) | 41.134 (32.709-49.56) |
| 2033 | 4722638.3 (3636689.5-5808587.1) | 40.423 (31.121-49.725) | 2173729.6 (1695106-2652353.2) | 39.59 (31.042-48.139) | 2548908.7 (1941583.5-3156233.9) | 40.9 (31.508-50.291) |
| 2034 | 4808406.6 (3581030.1-6035783) | 40.262 (29.977-50.548) | 2214438.8 (1671463.4-2757414.1) | 39.385 (29.901-48.87) | 2593967.8 (1909566.7-3278368.8) | 40.668 (30.284-51.053) |
| 2035 | 4894422 (3515812.8-6273031.2) | 40.106 (28.8-51.412) | 2254953.7 (1643290.5-2866616.8) | 39.167 (28.726-49.608) | 2639468.3 (1872522.3-3406414.4) | 40.44 (29.036-51.843) |
| 2036 | 4981141 (3441019.7-6521262.3) | 39.966 (27.597-52.334) | 2295602.6 (1610663.6-2980541.7) | 38.949 (27.528-50.37) | 2685538.4 (1830356.2-3540720.6) | 40.223 (27.773-52.673) |
| 2037 | 5068603.8 (3356267.6-6780939.9) | 39.851 (26.375-53.328) | 2336746.3 (1573623-3099869.6) | 38.748 (26.32-51.176) | 2731857.4 (1782644.5-3681070.3) | 40.021 (26.496-53.545) |
| 2038 | 5157293.2 (3261729.6-7052856.8) | 39.753 (25.126-54.38) | 2378470.9 (1532175.5-3224766.3) | 38.55 (25.093-52.007) | 2778822.3 (1729554.2-3828090.4) | 39.825 (25.202-54.447) |
| 2039 | 5247569.1 (3157282.8-7337855.4) | 39.662 (23.844-55.479) | 2420731.4 (1486153.9-3355308.8) | 38.343 (23.842-52.843) | 2826837.7 (1671128.9-3982546.6) | 39.629 (23.888-55.37) |
| 2040 | 5337979.8 (3041569-7634390.6) | 39.574 (22.527-56.622) | 2462656.4 (1434831.5-3490481.3) | 38.118 (22.565-53.672) | 2875323.4 (1606737.5-4143909.3) | 39.435 (22.556-56.313) |
| GBTC, gallbladder and biliary tract cancer; ASR, age-standardized rate; UI, Uncertainty interval; | | | | | | |

**Supplementary Figure 1**


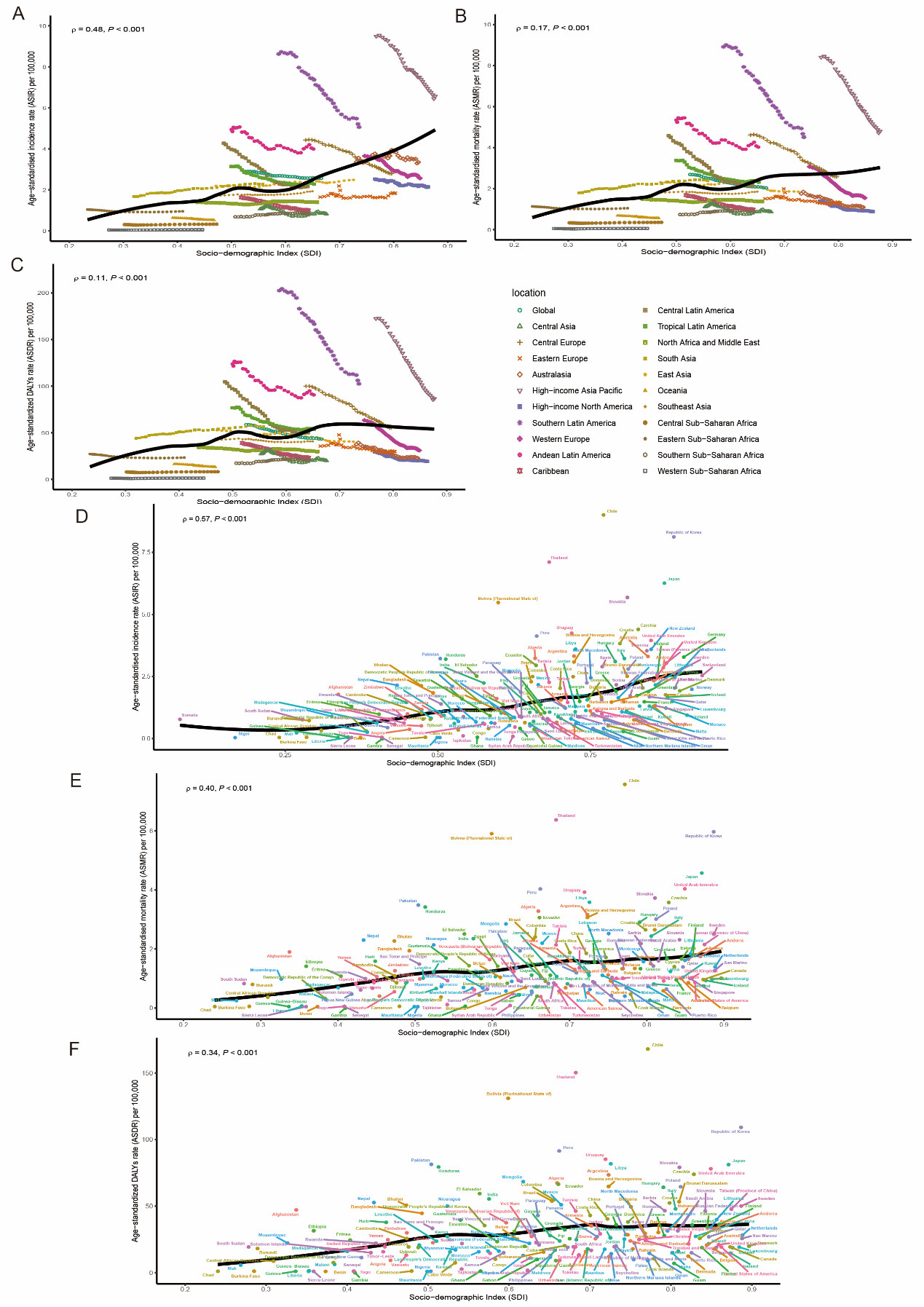


Supplementary Figure 1. The trend of ASIR, ASMR, and ASDR by SDI from 1990 to 2021 at the global, regional and national levels. A-C The trend of ASIR, ASMR, and ASDR by SDI from 1990 to 2021 at the global and regional levels, A for ASIR, B for ASMR, and C for ASDR; D-F The trend of ASIR, ASMR, and ASDR by SDI from 1990 to 2021 at national levels, D for ASIR, E for ASMR, and F for ASDR. ASIR, age-standardized incidence rate; ASMR, age-standardized mortality rate; ASDR, age-standardized DALYs rate; SDI, socio-demographic index.

**Supplementary Figure 2.**


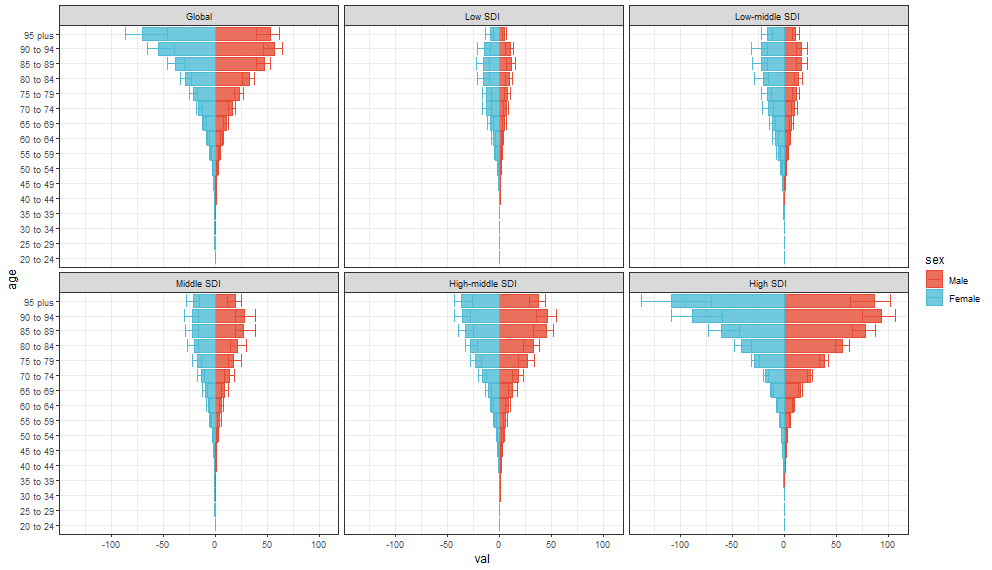


# Supplementary Figure 2. The GBTC incidence rate between genders across different SDI regions.

GBTC, gallbladder and biliary tract cancer; SDI, socio-demographic index

**Supplementary Figure 3**

**
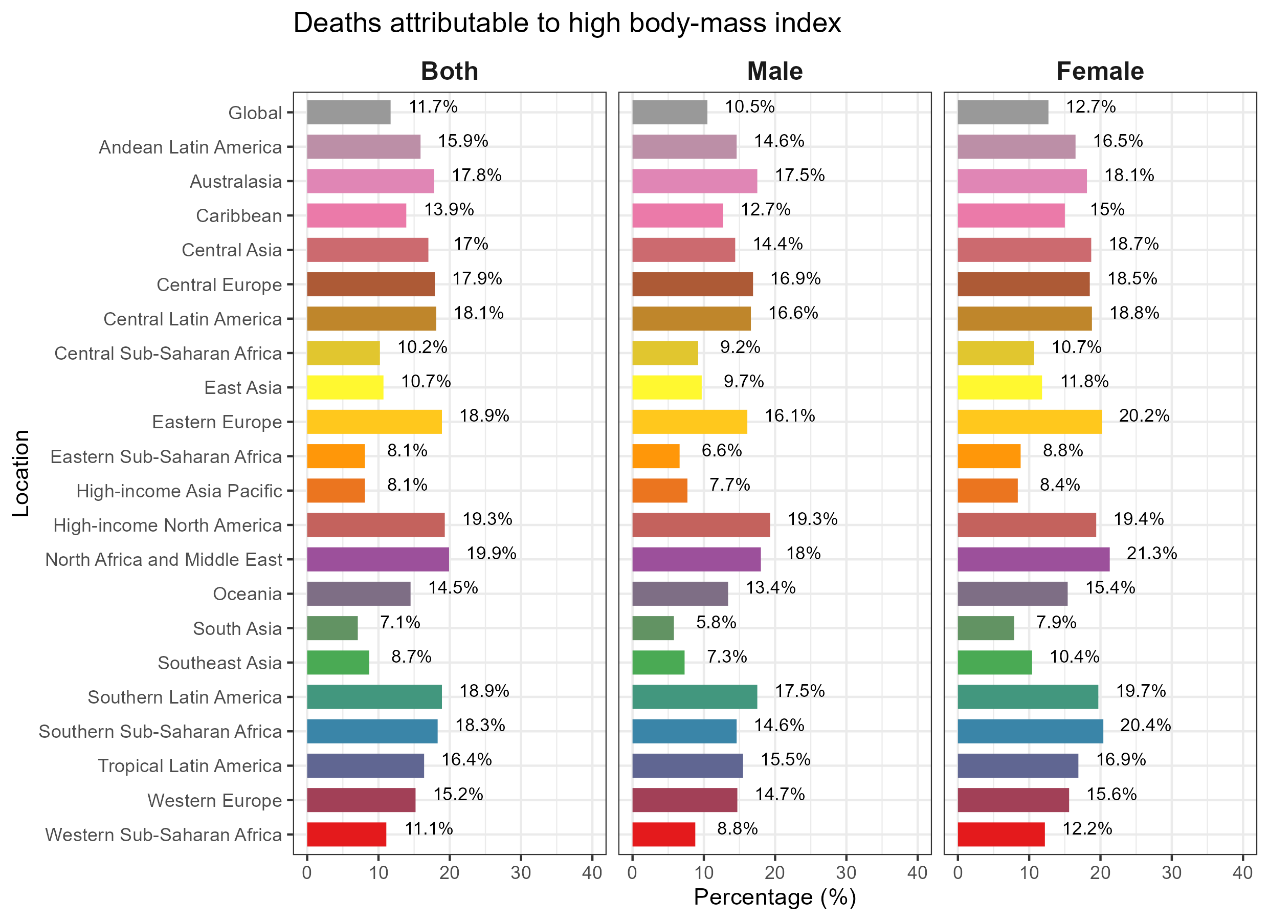
**

Supplementary Figure 3. Deaths risk factor attributable to high BMI. BMI, body mass index
